# Genome Sequence and Transcriptome Analysis of the Radioresistant Bacterium *Deinococcus gobiensis*: Insights into the Extreme Environmental Adaptations

**DOI:** 10.1371/journal.pone.0034458

**Published:** 2012-03-28

**Authors:** Menglong Yuan, Ming Chen, Wei Zhang, Wei Lu, Jin Wang, Mingkun Yang, Peng Zhao, Ran Tang, Xinna Li, Yanhua Hao, Zhengfu Zhou, Yuhua Zhan, Haiying Yu, Chao Teng, Yongliang Yan, Shuzhen Ping, Yingdian Wang, Min Lin

**Affiliations:** 1 Biotechnology Research Institute, Chinese Academy of Agricultural Sciences, Beijing, People's Republic of China; 2 College of Life Sciences, Beijing Normal University, Beijing, People's Republic of China; Louisiana State University and A & M College, United States of America

## Abstract

The desert is an excellent model for studying evolution under extreme environments. We present here the complete genome and ultraviolet (UV) radiation-induced transcriptome of *Deinococcus gobiensis* I-0, which was isolated from the cold Gobi desert and shows higher tolerance to gamma radiation and UV light than all other known microorganisms. Nearly half of the genes in the genome encode proteins of unknown function, suggesting that the extreme resistance phenotype may be attributed to unknown genes and pathways. *D. gobiensis* also contains a surprisingly large number of horizontally acquired genes and predicted mobile elements of different classes, which is indicative of adaptation to extreme environments through genomic plasticity. High-resolution RNA-Seq transcriptome analyses indicated that 30 regulatory proteins, including several well-known regulators and uncharacterized protein kinases, and 13 noncoding RNAs were induced immediately after UV irradiation. Particularly interesting is the UV irradiation induction of the *phrB* and *recB* genes involved in photoreactivation and recombinational repair, respectively. These proteins likely include key players in the immediate global transcriptional response to UV irradiation. Our results help to explain the exceptional ability of *D. gobiensis* to withstand environmental extremes of the Gobi desert, and highlight the metabolic features of this organism that have biotechnological potential.

## Introduction

The order Deinococcales contains 50 species of extremely ionizing radiation (IR) and UV tolerant bacteria (http://www.bacterio.cict.fr/) [1]. *D. radiodurans* R1, isolated from canned meat that had spoiled following exposure to X-rays, was sequenced first [2]. *D. radiodurans* has 200-fold greater resistance to ionizing radiation and 20-fold greater resistance to UV radiation than *Escherichia coli* [3], but it encodes approximately the same number and types of DNA repair proteins as *E. coli*, and no unique DNA repair system was found [3], [4]. Recently, the genome sequences of the slightly thermophilic *D. geothermalis* DSM11300, isolated from a hot spring, *D. deserti* VCD115, isolated from the Sahara desert, *Truepera radiovictrix* RQ-24, isolated from hot spring runoff on the Island of Sao Miguel, and *D. maricopensis* LB-34, isolated from the Sonoran Desert soil, were published [5]–[8]. Besides, the sequence of the complete genome of *D. proteolyticus* MRP is available under GenBank accession number CP002536. Investigation of the biology and biochemistry of *Deinococcus spp*. has benefited from the availability of genomic information and the development of genetic tools, but the extreme resistance phenotype of *Deinococcus spp*. is still not fully understood [9]. Comparative genomics combined with microarray and proteomic analysis suggest that the extreme resistance phenotype results from a combination of different molecular mechanisms [5], [6], [10]–[12].

About 10% of the Earth's terrestrial surface is covered by desert. The Gobi desert of northwestern China is a cold, arid biotope with cycles of extreme temperatures, prolonged dryness, and intense solar radiation [13]. Despite the extreme challenges of the desert, diverse microorganisms have adapted and colonized this harsh environment. Thus, the desert biosphere is an excellent venue for studying evolution under extreme conditions, and it provides a useful gene pool for genetic engineering. One of the major stresses for bacteria inhabiting the surface sands of the desert is intense solar UV radiation-induced damage. In general, the capacity of prokaryotes to withstand significant UV radiation requires a wide array of physiological responses, including transcriptional regulation and cellular repair of irradiation-induced damage [11], [14], [15]. Several studies have focused on cellular recovery following exposure to UV irradiation and have shown that induction and repression of UV radiation-responsive genes occurs in a time-dependent manner [16]–[18]. Currently, very little is known about the immediate transcriptome response to UV irradiation.

We recently characterized a new bacterial species, *Deinococcus gobiensis* I-0, that was isolated from the upper sand layers of the cold Gobi desert of the Xinjiang region in China [19]. This strain shows higher tolerance for gamma radiation and UV light than all other known *Deinococcus* strains [19]. To obtain a comprehensive understanding of the molecular mechanisms underlying the resistance phenotype of *Deinococcus*, the genome of *D. gobiensis* was sequenced and compared to those of the three most closely related sequenced bacterial strains, *D. radiodurans* R1, *D. geothermalis* DSM11300, and *D. deserti* VCD115, which were isolated from canned meat, a hot spring, and the hot Sahara desert, respectively [3]–[6]. This study also provides the first transcriptome analysis investigating the UV resistance of *Deinococcus*. In particular, we identified a subset of poorly characterized UV irradiation-induced genes that may provide clues to the adaptation of *Deinococcus* to extreme environments.

## Results

### Genome features

**Figure 1:**
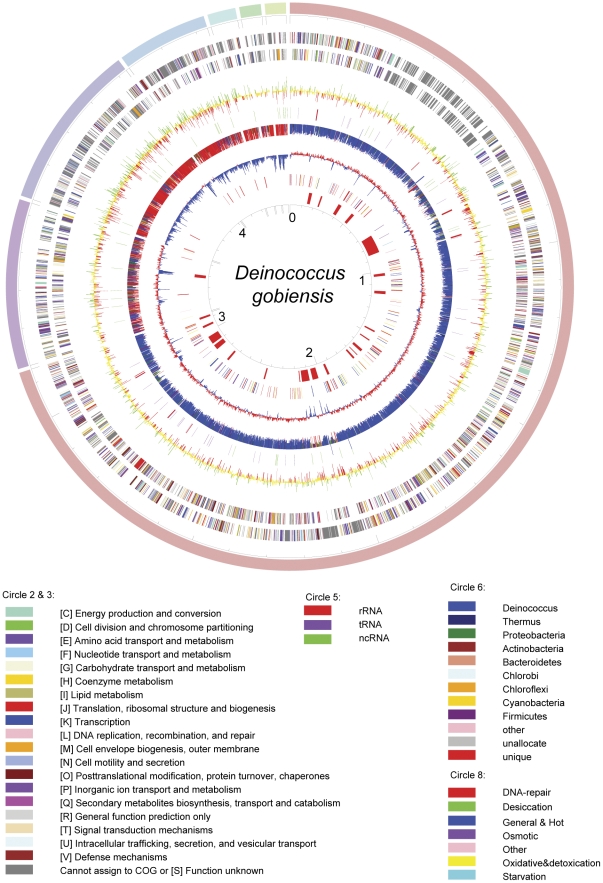
*D. gobiensis* I-0 genome structure. The seven replicons were opened at sequence position 1 and concatenated. Circle 1, red, chromosome (3.1 Mb); violet, plasmid 1 (P1, 433 kb); indigo, P2 (425 kb); blue, P3 (232 kb); light blue, P4 (72 kb); dark green, P5 (55 kb); light green, P6 (53 kb). Circles 2 and 3, predicted protein coding sequences (CDSs) clockwise and anticlockwise, respectively. Coloring is according to COG. Circle 4, Fold change in the immediate global transcriptional response to UV irradiation for each gene: green, upgulated; red, down-regulated; yellow, not changed significantly. Circle 5, red, rRNA; purple, tRNA; green, ncRNAs (noncoding). Circle 6, blue, genes with homologues in other *Deinococcus* genomes; red, genes found only in *D. gobiensis* I-0; other colors, genes with closest homologues in other phyla. Circle 7, deviation from the average 69.15% total genomic GC content: red, higher; blue, lower. Circle 8, previously reported genes that are involved in DNA repair and stress-responses. Circle 9, location of the 23 genomic islands. Circle 10, Mb scale.

The genome of *D. gobiensis* I-0 is composed of seven replicons: a 3.1 Mb main chromosome and six plasmids from 433 to 53 kb (Figure 1 and Table 1, GenBank accession numbers CP002191–CP002197 for the main Chromosome and Plasmids P1–P6, respectively). The chromosome and the 433 kb plasmid P1 have an average GC content of 71%, higher than that of the six other sequenced Deinococcales species (Table S1), and similar to that of the extreme thermophile *Thermus thermophilus* [20]. The genome of *D. gobiensis* contains 4,340 predicted coding sequences (CDSs), 46 tRNA genes, and 15 rRNA genes, and is larger than those of the six other published Deinococcales species (Table S1).

**Table 1:** General features of the *D. gobiensis* genome.

Molecule	Chromosome	Plasmids						All
		P1	P2	P3	P4	P5	P6	
Size(bp)	3,137,147	432,699	424,524	231,600	72,036	54,602	53,428	4,406,036
GC content (%)	70.8	69.81	63.89	62.97	60.44	61.87	54.72	69.15
Coding density (%)	84.8	86	83.8	83.7	80.2	74.9	74.1	85.4
Protein-coding genes	2959	383	523	282	70	79	44	4340
(Average length, nt)	(899)	(971)	(679)	(687)	(825)	(511)	(900)	(863)
Pseudo genes	19	0	0	0	0	0	0	19
tRNAs	46	-	-	-	-	-	-	46
5 S rRNA	5	-	-	-	-	-	-	5
16 S rRNA	5	-	-	-	-	-	-	5
23 S rRNA	5	-	-	-	-	-	-	5
ncRNA	19	7	7	5	1	0	6	46

**Figure 2:**
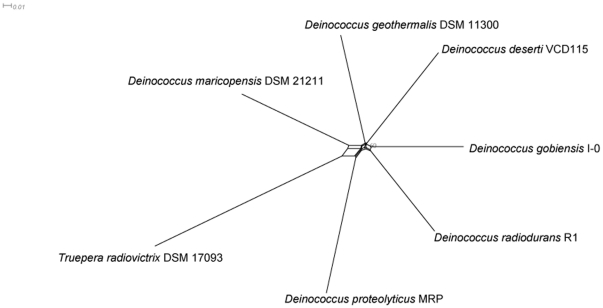
Unrooted Deinococcales neighbor-joining phylogenetic tree deduced from the nucleotide acid sequences of the orthologous proteins that occur in all 14 sequenced strains from the phylum Deinococcus-Thermus. *D. gobiensis* and *D. radiodurans* are most closely related. Numbers indicate bootstrap values below 100.

Phylogenetic analyses using only the orthologous proteins that occur in 14 sequenced strains from the phylum Deinococcus-Thermus showed that *Deinococcus* strains belong to the same branch (Figure S1). Further phylogenetic analyses showed that *D. gobiensis*, *D. radiodurans*, *D. geothermalis* and *D. deserti* belong to the same deeper branch and *D. gobiensis* was more closely related to *D. radiodurans* than to *D. deserti* and *D. geothermalis* (Figure 2). Pairwise comparisons of the *Deinococcus* genomes revealed limited synteny (Figure S2). The most striking feature was the cross patterns that indicate frequent symmetrical exchanges of genes, possibly by recombination between the bidirectional replication forks [21], [22]. There were no long stretches of synteny, indicating that the *Deinococcus* genomes exhibit remarkable plasticity involving gene rearrangements, acquisition, and loss (Figure S2). A 354-kb stretch of the main chromosome that is populated by 316 genes lacks a single functional COG prediction (Figure 1, circles 2 and 3; grey DGo_CA0296–DGo_CA0612, 306 kb–660 kb). BLAST searches revealed that 262 (83%) of the 316 genes have counterparts in other *Deinococcus* species, although they were dispersed throughout the genomes. We suggest the name “Grey Heaven of *D. gobiensis*” for this intriguing gene cluster which appears to underpin the unprecedented resistance of deinococci.

**Figure 3:**
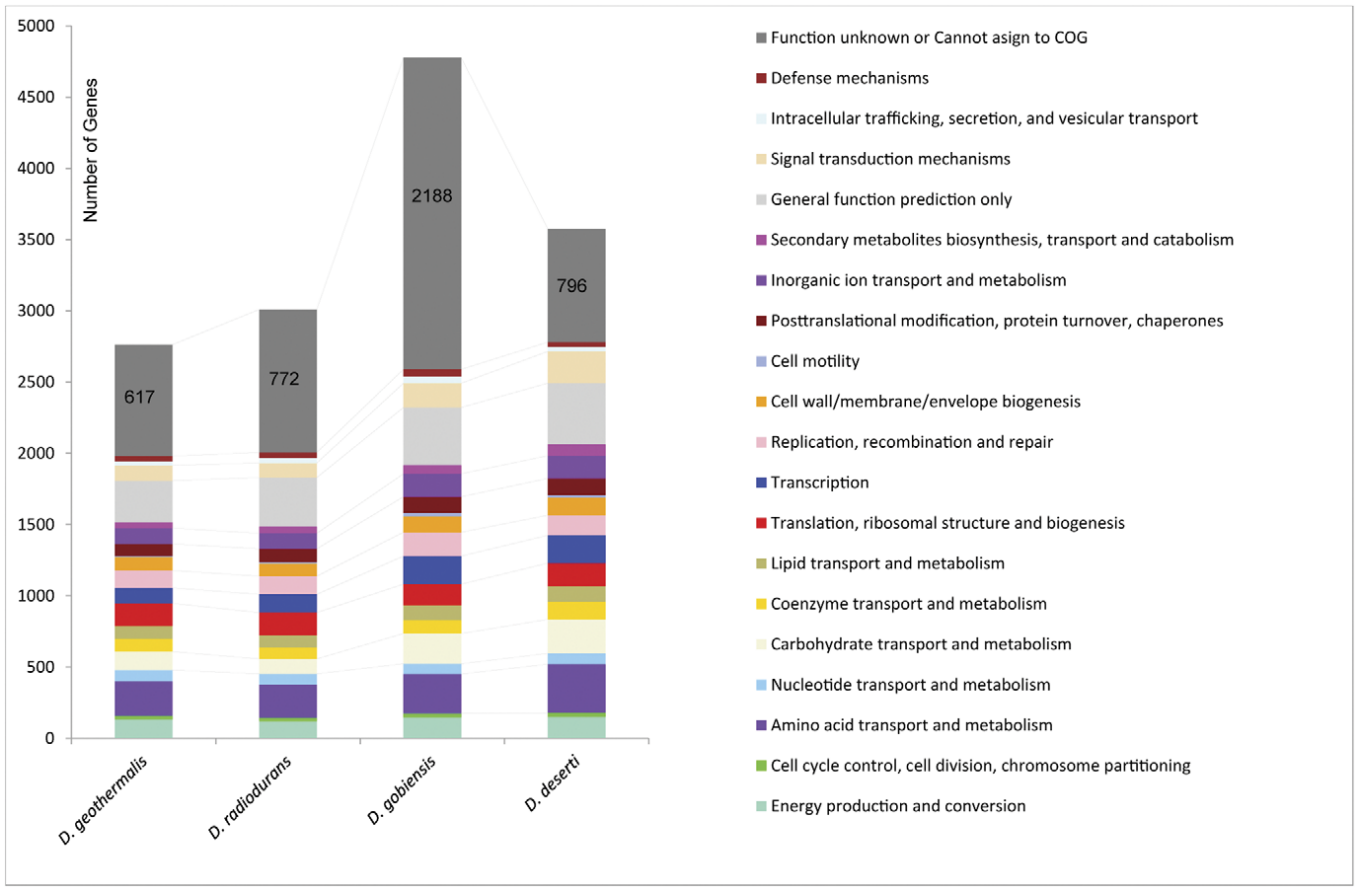
COG functional categories in the four *Deinococcus* species. Standard colors were used to indicate the COG functional categories. The numbers of unassigned and function unknown genes are indicated in the grey areas.

Of the 4,340 CDSs, 55% (2,376/4,340) were classified by Clusters of Orthologous Groups (COGs), a lower percentage than for the other deinococci (75% in *D. radiodurans*, 85% in *D. deserti* and 79% in *D. geothermalis*). 1,534 (35%) of the *D. gobiensis* CDSs were hypothetical proteins, and 834 (19%) were orphans without precedent. To identify the functional gene categories that characterize the cold desert adaptation in deinococci, we compared the genomes of *D. gobiensis* and other three related *Deinococcus* species (Figure 3). *D. gobiensis* and *D. deserti* are similar in each COG category. Notably, the two desert species have more genes than the other two published species in most categories, especially in energy production and conversion, carbohydrate transport and metabolism, transcription, cell wall/membrane/envelope biogenesis, inorganic ion transport and metabolism, signal transduction mechanisms. *D. gobiensis* also has more genes for replication, recombination and repair, cell motility, intracellular trafficking, secretion, and vesicular transport, and defense mechanisms, but fewer genes for translation and nucleotide metabolism genes. This COG characteristic is similar to functional gene categories that are associated with cold adaptation of the psychrophilic *Methanococcoides burtonii* [23].

### Transcriptome profiling

The transcriptome was analyzed using high throughput cDNA sequencing (RNA-Seq), that uses deep sequencing to assess the transcriptional activity of annotated genes, reveal previously unannotated genes and identify non-coding RNAs (ncRNAs) [24], [25]. We found that 4,325 of the 4,340 CDSs and 45 non-coding RNAs were detected under normal growth conditions (The RNA-Seq raw data of are available from the NCBI Gene Expression Omnibus (http://www.ncbi.nlm.nih.gov/geo/) under accession number GSE29088). The accuracy of the RNA-seq data was verified by quantitative real-time RT-PCR analysis (using 16 S rRNA as a control) of eight selected genes qRT-PCR (Table S2). In total, 638 plausible transcriptional start sites (TSS) were identified on the chromosome, and 362 on the six plasmids (Table S3). Furthermore, cDNAs for almost all of the CDSs of unknown function (2,029/2,041) were detected.

Bacteria generally have a global transcriptional response after UV irradiation that initiates subsequent recovery from the radiation damage. Immediately following UV irradiation, a total of 390 genes (9% of the genome) were induced, and 754 genes (17%) were repressed (Table S4). Approximately 8% of all *D. gobiensis* genes were predicted to be involved in regulation, which is the same as for *D. radiodurans* and also for *E. coli*. The regulators include six sigma factors (*rpoD*, *sigE*, *sigK*, *sig3*, *sig4* and *sig5*), 54 sensor kinases and 129 transcriptional regulators. Among those, 30 were induced, and 60 were repressed. In bacteria, noncoding RNAs often coordinate the adaptation to environmental changes, integrate environmental signals, and control target-gene expression [26]. We observed that 13 ncRNAs were upregulated, and 22 were downregulated. Moreover, a total of 61 predicted proteins were constitutively expressed at high levels (≥1*%* of total mRNA) before and after UV irradiation, including a heat shock protein (IbpA), a phage shock protein (PspA), cold shock proteins (PprM1 and PprM2), DNA damage response proteins (DdrE and DdrO), chaperones (DnaK, DnaJ, GrpE and GroEL) and CDSs of unknown function, some of which may be responsible for cold desert adaptation.

### *Deinococcus*-specific genes and horizontal gene transfer

The six sequenced *Deinococcus* strains share 1,474 genes. Among them, 39 genes of unknown function had no close orthologs in other species that don't belong to Deinococcales (Table S5). Furthermore, 10 previously predicted DNA damage-responsive genes (*ddr*) induced in response to IR and desiccation in *D. radiodurans* [14] were also found in *D. gobiensis*. Recently, *ddrB* was identified as a member of a new family of bacterial single-stranded DNA binding proteins which are induced by ionizing radiation [27], [28]. We observed that the expression of some *ddr* genes was induced significantly by UV, including *ddrA* (a double-strand break repair protein), *ddrB* (a radiation-induced single-stranded DNA binding protein), *ddrC*, *ddrE*, *ddrI* and *ddrP*. Although the precise functions of these genes are not clear, they may contribute to a novel mechanism for adaptation to the extreme desert environment.

Horizontal gene transfer has played a major role in the evolution of bacteria and archaea. The total genome of *D. gobiensis* is about 1 Mb larger than the other *Deinococcus* genomes, probably due to gene acquisition by horizontal gene transfer. *D. gobiensis* contains more DNA sequences that are indicative of mobile elements than the other deinococci, including 77 putative transposases, 17 integrases or recombinases, and 2 prophages. In addition, we found that the *D. gobiensis* plasmid P1 was similar to the *D. deserti* plasmid P2, the *D. geothermalis* plasmid pDSM11300 (DG574) and the *D. radiodurans* chromosome 2, suggesting that these similar sequences may have come from a common ancestor (Figure 1 and Figure S2). The *D. gobiensis* plasmids P2–P6 contain a high proportion of genes that were exclusive to *D. gobiensis* (Figure 1). Furthermore, most of the genes on *D. gobiensis* P3–P6 had no homologs in the NCBI database. This indicated that the *D. gobiensis* plasmids have different evolutionary histories. Furthermore, the *D. gobiensis* genome contained 23 sequences, which were classified as genetic islands (GI) and may have been acquired by horizontal gene transfer (Table S6). For example, genes for tRNAs flank GI 19, while the others contain integrase genes (GI 7–10 and 15). In addition, GIs 9 and 10 resemble prophages, and most of their genes have their closest homologs outside the genus *Deinococcus*.

The closest ortholog of the 2,728 CDSs in the *D. gobiensis* genome was in the phylum Deinococcus-Thermus. However, 1,612 CDSs were indicative of horizontal gene transfer, 691 of which seemed to originate from *Proteobacteria*, *Actinobacteria* and *Firmicutes*. These horizontally acquired genes are mostly involved in sugar transport, signal transduction, transcriptional regulation and transposition. Notably, the two desert species *D. gobiensis* and *D. deserti* contain more insertion sequence (IS) associated orphan genes without *Deinococcus* orthologs than *D. radiodurans*. This difference likely reflects the evolutionary development of the efficient adaptive strategy derived by IS-associated horizontal acquisition of additional genes. Further transcriptome analysis indicated that the expression of more than 35% of the horizontally acquired genes were changed by UV light, strongly suggesting the involvement of these genes in resistance to UV irradiation.

### Metabolic pathways and transport

As expected, the *D. gobiensis* genome possesses most of the genes for the central metabolic pathways, including glycolysis, the pentose phosphate (PP) pathway, the tricarboxylic acid (TCA) cycle, biosynthesis of amino acids, and *de novo* purine and pyrimidine biosynthesis. This set of predicted pathways is similar to that found in the other *Deinococcus* strains. Transport systems are key components for the communication of bacteria with their environments. For example, *D. gobiensis* has approximately 300 genes coding for transporters. We found that the *rbs* genes encoding ribose ABC transporters are present in two desert species but absent from *D. radiodurans* and *D. geothermalis*. In addition to having more Mn ABC transporter proteins that are similar to those found in *D. deserti*, *D. gobiensis* is also rich in ABC transporters for oligopeptides, amino acids and sugars relative to *D. radiodurans* and *D. geothermalis*. Eighteen of the 80 amino acid or peptide transporter proteins and 16 of the 54 proteins for sugar transport are specific to *D. gobiensis*.

Significant induction of most of the genes encoding metabolic pathways was not detected immediately after UV irradiation. Specifically, among the 286 transporters, 22, including two for K^+^ (TrkG and KdpC) and PO_4_^3−^ (PstA and PstC), were induced and 45, including two glycerol transporters and three ribose transporters, were repressed. In addition, we observed strong repression of the *zwf* gene (glucose 6-phosphate dehydrogenase), which is the rate-limiting enzyme of the PP pathway required for removal of reactive oxygen species (ROS). We also found that the two adjacent operons *nuoAB* and *nuoC-N* encoding NADH-quinone oxidoreductase were significantly downregulated, and NADH:quinone oxidoreductase plays a pivotal role in cellular energy production. Also among repressed genes were the *aceB* gene that encodes a malate synthase for the glyoxylic acid shunt, the genes responsible for the synthesis of amino acids (including arginine, ornithine, tryptophan, aspartic acid and lysine), and the genes *fabZ* and *fabG* that encode NADPH-dependent enzymes involved in the synthesis of fatty acids and biotin. This repression can be explained by the induction of *relA*, an inducer of the stringent response involved in ppGpp synthesis. We hypothesize that desert-dwelling bacteria could have specialized mechanisms to reduce their metabolism. This metabolic switch may be a general response for coping with solar UV radiation in a desert environment and could be the subject of further experimental work.

### DNA repair and associated systems

The extremely radiation-resistant bacteria must have highly efficient and specialized DNA repair systems [3]–[6]. *D. gobiensis* contains sets of genes encoding proteins for various DNA repair pathways (Table S7 *A*) and, like *D. radiodurans*, *D. gobiensis* has only 15 orthologs of the 31 genes that comprise the LexA-RecA-mediated SOS response to UV in *E. coli* [29]; *D. gobiensis* lacks homologs of the error-prone DNA polymerase V genes *umuC* and *umuD*, which are important for the SOS response in many bacteria [30]. We found, however, many *Deinococcus*-specific genes that are probably involved in DNA repair and extreme resistance. Unexpectedly, most of these genes were not changed in their expression immediately after UV irradiation.

UV radiation generates two major DNA damage products, the cyclobutane pyrimidine dimer and the pyrimidine pyrimidinone dimer. Photoreactivation mediated by photolyase is one of the simplest and oldest repair systems for UV-induced cyclobutane-pyrimidine dimers. The two desert species, *D. gobiensis* and *D. deserti*, contain a homolog (DGo_PA0134 and Deide_3p02150) of the *splB* gene encoding an active spore photoproduct lyase belonging to the radical S-adenosylmethionine superfamily [31], which is absent from both *D. radiodurans* and *D. geothermalis*. Additionally, *D. gobiensis* encodes another photolyase (PhrB, DGo_CA0607) whose closest homolog is from the Archaea. In contrast to photoreactivation, excision repair pathways are much more complex and can be separated into base excision repair (BER) and nucleotide excision repair (NER). The *D. gobiensis* genome contains sets of the excision repair genes, including nine genes for BER and six for NER (Table S7 *A*), which may play in important role in UV-induced DNA repair.

A recombinational repair pathway is also operative in various organisms. As a part of the recombinational DNA repair of UV-lesions, *E. coli* RecA protein has a regulatory role in lesion bypass through coprotease activity which includes stimulation of self-cleavage of the repressor LexA. In *D. gobiensis* we observed a two-fold induction of *lexA* by UV irradiation, while *recA* was unchanged. This observation was very different from the response of *E. coli* in which both genes were induced by UV irradiation [16]. RecB is critical for the enzyme activity of the multifunctional exonuclease V (RecBCD) involved in DNA degradation [32]. *D. gobiensis* contains two plasmid-encoded homologs of *recB* (DGo_PB0022 and DGo_PC0098), and the expression of DGo_PC0098 was three-fold induced by UV irradiation. In a previous study, expressing the *E. coli recB* in *D. radiodurans* increased UV resistance [33]. Because the other *Deinococcus* strains lack *recB*, UV irradiation-induced expression of *recB* may contribute to the extreme UV resistance, and it is worth noticing that *recB* is absent in the other three published *Deinococcus* genomes. The two desert species, *D. gobiensis* and *D. deserti*, have a homolog of *polB* encoding DNA polymerase II (DGo_PC0151 and Deide_1p00180), which is essential for resumption of DNA replication after UV exposure [34] and may also be involved in DNA repair. However, *D. radiodurans* and *D. geothermalis* lack *polB*. Further analysis showed that expression of *polB* increased significantly following UV irradiation, whereas *dnaE* encoding the alpha subunit of DNA polymerase III, which is required for misincorporation and bypass during UV mutagenesis, was slightly repressed.

### Reactive oxygen species detoxification

The ability to survive acute or chronic exposure to ionizing and UV irradiation and desiccation can be attributed to prevention, tolerance, and repair mechanisms. Scavenging oxygen radicals is an important component of prevention mechanism because reactive oxygen species (ROS) are key intermediates in the damage to cells caused by ionizing and UV radiation and desiccation. Several such prevention gene products are present in the *D. gobiensis* genome, including five catalases, four superoxide dismutases, and several regulatory genes, for example OxyR of the LysR family of proteins that activates the transcription of genes involved in peroxide metabolism and protection (*katG*, *ahpC*, *ahpF*, and *dps*), redox balance (*grxA*, and *trxC*). Genes involved in carotenoid biogenesis have been shown to confer a modest level of radiation resistance by scavenging electrons from ROSs [35]. *D. gobiensis* produces carotenoids, and the carotenoid biosynthetic pathway is similar to that found in *D. radiodurans* [36]. In addition, we observed that genes for secondary metabolite biosynthesis of carotenoids, vitamin B1, vitamin B12, NAD^+^ and cytochrome P450 were strongly downregulated after UV irradiation.

*D. gobiensis* possesses two of the three types of known Mn^2+^ transporters: one from the natural resistance-associated macrophage family and one from the ATP dependent ABC-type transporter family. Interestingly, we observed that *D. gobiensis* (the Mn/Fe ratio of 1.60±0.02) accumulated 1.1 times more Mn and 2.4 times less Fe than *D. radiodurans* R1 (Mn/Fe ratio of 0.60±0.04). Previous studies of stress response systems in *D. radiodurans* demonstrated that the dose-response relationship for desiccation killing in bacteria isolated from desert environments parallels the levels of protein oxidation and the Mn/Fe ratios [37]. A first line of defence against ionizing radiation might be the accumulation of manganese complexes, which can prevent the production of iron-dependent reactive oxygen species [38]. More recently, the quantitative measurement of proteome oxidation (i.e., protein carbonylation) in *D. radiodurans* exposed to ionizing radiation or UVC light has revealed a consistent correlation with cell killing [39]. A comprehensive outlook on *D. radiodurans* strategies of combating oxidative stress suggests that the level of protein damage together with the cellular ROS-scavenging capacity determine the radiation survival of bacteria [12]. Thus, it is likely that in *D. gobiensis*, accumulation of high level of Mn may contribute to the enhanced tolerance to ionizing radiation or UV light.

## Discussion

The surface sands of the desert are exposed to intense solar radiation, cycles of extreme temperatures, and desiccation. Such extreme conditions cause stress-induced damage to DNA and proteins, which is lethal to most organisms. Therefore, desert-dwelling bacteria protect DNA and proteins from damage and/or repair them efficiently. Two striking results of this work came from comparison of *D. gobiensis* with three other sequenced *Deinococcus* species isolated from canned meat, hot springs and the Sahara desert, respectively. Despite their phylogenetic differences, the two desert strains, *D. gobiensis* I-0 and *D. deserti* VCD115, have a large repertoire of similar genes. The two desert strains contain surprisingly large numbers of probably horizontally acquired genes and diverse mobile elements. Many genes shared by the two desert strains are associated with putative mobile elements that aided the parallel evolution of the two desert species. However, *D. gobiensis* and *D. deserti* were isolated from very different deserts: the cold Gobi, and the hot Sahara. Comparative analyses of the two desert strains revealed two distinct gene sets: a core of shared orthologous genes and, species-specific genes. Interestingly, *D. gobiensis* contains 1,541 genes that are missing from *D. deserti*, including genes for a glucose-6-phosphate dehydrogenase, two transketolases, four catalases, two superoxide dismutases, an alkyl hydroperoxide reductase, and a putative glutathione-S-transferase, which are probably involved in adaptation to a cold desert environment. Notably, in *D. gobiensis*, *phrB* and *recB* were induced immediately after UV irradiation, whereas the other *Deinococcus* strains lacked the *phrB* and *recB* genes. This seemed advantageous for *D. gobiensis* to make use of the repair systems associated with *phrB* and *recB*, since *phrB* encodes a photolyase that breaks pyrimidine dimers typically caused by UV exposure [40] and *recBCD* encodes a multifunctional enzyme involved in DNA degradation [32].

Depletion of the stratospheric ozone layer causes increases in UV radiation at the Earth's surface, and the molecular basis of extremely UV radiation-resistant phenotypes is one the intriguing problems of modern biology. The success of *D. gobiensis* in the cold Gobi desert is probably due to its specialized metabolism, complex regulatory mechanisms, and robust repair systems. Indeed, *D. gobiensis* was isolated from the upper sand layers of a cold desert where bacteria are frequently exposed to long-lasting solar UV irradiation, necessitating a specific regulatory response that precedes the cellular recovery after UV irradiation damage. Several studies on UV radiation resistance have been conducted on exponential phase cells recovering from ionizing and UV radiation. In one such study, transcriptome dynamics of *D. radiodurans* recovering from ionizing radiation indicated that the maximum response for most functional gene groups occurred concurrently at approximately three hours post-exposure [11]. UV radiation induces both upregulation of the *nos* gene and cellular nitric oxide (NO) production in *D. radiodurans*, and subsequently NO upregulates *obgE*, a gene for an essential GTPase involved in the regulation of many growth-related processes [18]. These studies have provided important information about cellular recovery after UV irradiation. However, more studies of the transcriptome response immediately after UV irradiation are needed to establish a detailed understanding of the regulatory networks underlying the extreme resistance of deinococci. In the present study, we showed that the expression of most of the previously characterized genes, including *nos* and *obgE*, was not induced immediately after UV irradiation. Notably, the 30 regulatory genes induced immediately after UV irradiation included well-known regulators, such as two transcriptional activators (CarD and PhoR), a glucose-inhibited division protein A (GidA), a cognate response regulator (CitB) and a tryptophan repressor (TrpR), and also two uncharacterized protein kinases and 12 ncRNAs. The *carD* gene encodes an essential regulator of rRNA transcription necessary for the mycobacterial stringent response to oxidative stress, DNA damage, and nutrient limitation [41]. PhoR activates genes of the *E. coli* phosphate regulon in response to phosphate deprivation [42]. The *gidA* gene encodes the glucose-inhibited cell division protein A that controls the posttranscriptional regulation of quorum-sensing genes via RhlR-dependent and RhlR-independent pathways in *Pseudomonas aeruginosa* [43]. These proteins are likely key players in the global transcriptional response immediately following UV irradiation, preceding the cellular recovery of UV irradiation damage (Table 2).

**Table 2:** The *D. gobiensis* genes implicated in the immediate global transcriptional response to UV irradiation.

Locus_tag	Gene	Product description	Fold Change**
**Regulation**			
DGo_CA0550	*carD*	Transcriptional regulator, CarD family	2.2
DGo_CA0552	*ccpA*	Catabolite control protein A	3.4
DGo_CA0357	*citB*	Cognate response regulator	2.0
DGo_CA1040	*ddrI*	Transcriptional regulator, Crp/Fnr family	3.5
DGo_CA0719	*deoR* *	DeoR-family transcriptional regulator	2.1
DGo_CA2738	*fliY*	ABC-type amino acid transport/signal transduction system, periplasmic component	2.2
DGo_CA2290	*gidA*	Glucose-inhibited division protein A	2.4
DGo_CA2808	*lspA*	Lipoprotein signal peptidase	4.9
DGo_PC0175	*phoR*	Sensor protein, transcriptional activators	2.7
DGo_CA0725	*sig4*	RNA polymerase sigma factor	2.0
DGo_CA0977	*str*	Streptomycin 3′-kinase	2.1
DGo_PB0002	*trpR* *	Tryptophan repressor, LysR family transcriptional regulator	3.4
**DNA repair**			
DGo_CA2046	*ddrA*	Double-strand break repair protein	1.9
DGo_CA0350	*ddrB*	Single-stranded DNA binding protein	2.2
DGo_CA0002	*dnaN*	DNA polymerase III, beta subunit	2.4
DGo_CA1041	*gyrA*	putative DNA topoisomerase subunit A	2.0
DGo_CA0873	*gyrB*	putative DNA topoisomerase subunit B	2.0
DGo_PC0001	*lexA*	Repressor LexA	1.9
DGo_CA0607	*phrB* *	Deoxyribodipyrimidine photo-lyase type II	3.2
DGo_PC0151	*polB*	DNA polymerase II	2.0
DGo_PC0098	*recB* *	Predicted nuclease, RecB family	3.0
DGo_CA0376	*yqgF*	Putative Holliday junction resolvase	2.7
**Other functions**			
DGo_CA0071	*ddrC*	Uncharacterized DNA damage response protein	2.1
DGo_CA0988	*ddrE*	Putative zinc metal lopeptidase	1.9
DGo_CA2239	*ddrP*	Uncharacterized DNA damage response protein	1.9

* Genes that are identified in D. gobiensis but not in other published Deinococcus species.

** Change values are means of values obtained from two independent experiments.

Various DNA repair and stress response-related genes previously identified and many new gene products of potential interest for biotechnological applications were also found in *D. gobiensis* (Table S7). For example, the *D. gobiensis* genome encodes a *Deinococcus*-specific global regulator (DGo_CA2805) that is similar to IrrE, a global regulator from the extremely radiation-resistant *D. radiodurans* that confers enhanced salt tolerance in both *E. coli* and in the plant *Brassica napus* [44]. *D. gobiensis* contains two similar cold shock proteins (DGo_CA1136 and DGo_PA0041), with 65–70% identity to CspA from *E. coli* and CspB from *B. subtilis* that confer abiotic stress tolerance in transgenic plants and improved grain yield in maize under water-limited conditions [45]. Furthermore, approximately 47% of the *D. gobiensis* genes encode proteins of unknown function. We identified a subset of previously uncharacterized genes induced immediately following UV irradiation, suggesting that the organism's extreme resistance phenotype may be attributable to still unknown genes and pathways. It would be intriguing to investigate which of their products are required for the extreme resistance phenotype. To our knowledge, this is the first report of a transcriptome analysis immediately following UV irradiation. Taken together, our results highlight the exceptional ability of *D. gobiensis* to withstand environmental extremes. These findings may have significant potential for biotechnological and agricultural applications. Further investigations will reveal commonalities in the genetic basis of the UV response and provide insight into the molecular mechanisms underlying the extreme resistance phenotype of the genus *Deinococcus*.

## Materials and Methods

### Bacteria and growth conditions

Cells of *Deinococcus gobiensis* I-0 ( = DSM 21396) were grown in TGY broth (1.0% peptone, 0.5% yeast extract, 0.1% glucose) at 30°C. For genome sequencing, cells were harvested at the early stationary phase (5–10×10^8^ colony-forming units (CFUs) ml^−1^). For irradiation, cells were harvested at the late-log phase (≈2×10^8^ CFUs ml^−1^) and then washed twice with equal volumes of potassium phosphate buffer (100 mM, pH 7.0) as described previously [19]. UV light (254 nm, 200 µW cm^−2^) was used to irradiate a 20 ml suspension for 5 min in a 9 cm plate with stirring. As a control, an additional non-irradiated suspension was incubated for the same duration. After treatment, cells were harvested by centrifugation at 8,000 rpm for 3 min.

To test the concentrations of Mn and Fe, cells of strains I-0 and R1 were collected from late-log phase TGY cultures and dried by low-temperature vacuum drying. Measurement was performed at the Micro Structure Analytical Lab (Beijing) using Wavelength Dispersive X-Ray Fluorescence (WDXRF).

To test the utilization of aromatic substrates, cells from the TGY 48 h culture were washed twice and subsequently inoculated into Mineral Salt medium (benzoic acid 2 mmol/L, NaNO_3_ 0.5 g/L, K_2_HPO_4_ 0.65 g/L, KH_2_PO_4_ 0.17 g/L, MgSO_4_ 0.10 g/L) at a dilution of 1∶100.

### Genomic DNA extraction and whole-genome shotgun sequencing

Total DNA was isolated from *D. gobiensis* I-0 ( = DSM 21396) according to a published method described for bacteria [46]. Genome sequencing was performed at Tianjin Research Center for Functional Genomics and Biochip (Tianjin, China) using the Sanger/pyrosequencing strategy described previously [47]. The Roche 454 FLX gene sequencer (454 Life Sciences, Branford, CT) was used to generate 329,480 reads that were assembled into 287 contigs using the Newbler assembler. Artificial 1 kb reads representing the Roche/454 assembly were generated using mktrace in the Consed package (www.phrap.org/) and assembled with 11,444 ABI3730 reads (3.2 kb library) using PhredPhrap (www.phrap.org/). Possible misassemblies were corrected according to the mate-pair relationships of ABI3730 reads; gaps between contigs were closed by editing in Consed, custom primer walks, or PCR amplification.

### Genome annotation and analysis

Glimmer3 was initially used to identify putative CoDing Sequences (CDS), and tRNAs were predicted using tRNAscan-SE, and Artemis [48] was used to collate data and facilitate annotation. Function predictions were based on BLASTp similarity searches (E-value <10^−5^) in the non-redundant GenBank protein database (www.ncbi.nlm.nih.gov/protein), the SwissProt protein database (http://www.ebi.ac.uk/swissprot/), the clusters of orthologous groups (COG) database (www.ncbi.nlm.nih.gov/COG) and KEGG database (www.genome.ad.jp/kegg).

Pairwise genome comparisons of *D. gobiensis* with three other *Deinococcus* species were made using nucmer in Mummer 3 [49]. The minimum length of a cluster of matches, break length and maximum gap distance were set to 30 bp, 3 kb and 3 kb, respectively.

To analyze the taxonomic affiliations of *D. gobiensis* proteins, the BLAST hits with at least 95% of the highest score (E-value <10^−4^) to the RefSeq database (www.ncbi.nlm.nih.gov/RefSeq) were collected for each of the *D. gobiensis* proteins. For each query, if the taxonomic affiliations of all hits at the phylum level were the same, the query was considered affiliated with this taxon; otherwise, the taxon affiliation of the query was considered unresolved. For each of the genes belonging to the phylum Deinococcus-Thermus, the query was further assigned to the order Deinococcales or Thermales according to the taxonomic affiliation of the best hit.

*D. gobiensis* and another eight publicly available Deinococcus-Thermus genomes from NCBI databases were used in the comparisons. To ensure consistency, the annotations of all genomes were verified based on the similarity with proteins in *D. gobiensis* using tBLASTn [50], [51]. The sets of orthologous protein-coding genes were defined as mutual fully transitive reciprocal BLASTp [52] hits (E-value <10^−4^) [53]. The amino acid (for the whole phylum) and nucleic acid sequence (within the order Deinococcales)of each orthologous group was aligned using the CLUSTALW program version 2.0 [54]. For each data set, the phylogenetic relationship was estimated and tested in one thousand bootstrap samples using TREE-PUZZLE version 5.3 (general time reversible (GTR) +Γ4+I model of evolution with a BIONJ starting tree) [55]. The bi-partitions with at least 70% supports in the bootstrap test for each data set were recorded as “0/1” status and used to reconstruct the consensus sequence. The phylogenetic relationship of the consensus sequence was built using SplitsTREE 4 with the BioNJ model [56].

### Isolation and enrichment of mRNA, RNA processing and transcriptome sequencing

Bacterial cells were collected and ground into a fine powder in liquid nitrogen. Total RNA was isolated using Trizol reagent (Invitrogen), subsequently purified using RNeasy MinElute Cleanup Kit (Qiagen) and eluted in RNase-free water. Bacterial ribosomal RNAs were removed via a mixed treatment using the MICROB Express kit (Ambion) and the mRNA-ONLY Prokaryotic mRNA isolation Kit (Epeicentre® Biotech.) according to the manufacturer's instructions. Heating at 94°C fragmented the mRNA. First strand cDNA was synthesized with random hexamer primers, and second strand cDNA was synthesized with DNA polymerase. Double strand cDNA was end-repaired, a single adenosine was added, and the Illumina adapters were ligated. Gel-electrophoresis was used to select DNA fragments between 200–250 bp. Libraries were amplified by PCR using Phusion polymerase. Sequencing libraries were denatured with sodium hydroxide and diluted in hybridization buffer for loading onto a single lane of an Illumina GA flow cell. Cluster formation, primer hybridization and pair-end, 101×2 cycle, sequencing were performed using proprietary reagents according to the manufacturers' recommended protocols (https://icom.illumina.com/).

### Transcriptomics analysis and qRT-PCR verification

High-throughput cDNA sequencing was performed using the Firecrest, Bustard and GERALD programs [24]. The low quality bases (Q<5) at the ends of the reads were trimmed. The reads that were longer than 20 bps were kept and aligned to the *D. gobiensis* genome using Burrows-Wheeler Aligner (BWA) [57]. The reads that were mapped into the rRNA regions were not included in further analysis. A transcript coverage map was calculated based on the alignment of whole transcript reads. For each of the genes, the 5′-end of the translation regions were defined as positions supported by at least 5 reads summarized in both of the samples.

To identify the non-coding RNA, the continuous regions (≥30 bp) with an average sequencing depth of ≥15 times/bp in the intergenic regions were extracted and compared against GenBank using blastx and blastn. Those regions that lacked similarities to known protein coding genes or were similar to known ncRNAs were classified as possible non-coding RNAs.

To compare the different samples, the fragments per kb of CDS per million mapped reads (FPKM) value were used to normalize the data and represent the overall gene expression. The differently expressed genes between the two samples were selected according to their significance in Chi-square tests (p = 0.05, with Bonferroni correction) and at least 2-fold differences.

One hundred micrograms of total RNA was then synthesized into cDNA using ProtoScript® M-MuLV First Strand cDNA Synthesis Kit (NEB). The primers were designed by Perlprimer v1.1.19 [58]. The expression levels of the selected genes were determined using the 7500 Real-Time PCR System (Applied Biosystems) according to SYBR® Premix ExTaq™ Kit's manual (Takara) using 20 microliter system.

## Supporting Information

Figure S1 Unrooted neighbor-joining phylogenetic tree deduced from the orthologous proteins that occur in all 14 sequenced strains from the phylum Deinococcus-Thermus. *D. gobiensis* and *D. radiodurans* are most closely related. Numbers indicate bootstrap values below 100.
(TIF)

Figure S2 Synteny plots comparing *D. gobiensis* I-0 and the other three *Deinococcus* genomes. The dot plots represent nucmer alignments generated by MUMMER 3 of *D. gobiensis* on the x-axis and three other *Deinococcus* species on the y-axis. Forward matches are shown in red, and reverse matches are shown in blue. *dnaA* isat the bottom left of each plot. The red marks on the horizontal line representing the *D. gobiensis* genome indicate genomic islands.
(TIF)

Table S1 General features of the genomes of Deinococcales species.
(DOC)

Table S2 The qRT-PCR verification.
(XLS)

Table S3 Transcriptional start sites (TSS). First column presents the TSS location; the TSS and its strand are listed in the next two columns.
(DOC)

Table S4 Functional description of 1144 genes induced or repressed after UV-irradiation.
(DOC)

Table S5 Deinococcales-specific genes.
(XLS)

Table S6 Genomic islands in *D. gobiensis*.
(DOC)

Table S7 DNA repair genes (A), stress response-related genes (B) and additional enzymes of possible biotechnological interest genes (C) identified in sequenced deinococci.
(DOC)
