# Supplementary figures and images for: Genome Sequence and Transcriptome Analysis of the Radioresistant Bacterium Deinococcus gobiensis: Insights into the Extreme Environmental Adaptations
Source: PLoS One. 2012 Mar 28;7(3):e34458. doi: 10.1371/journal.pone.0034458 (PMC3314630; doi:10.1371/journal.pone.0034458)

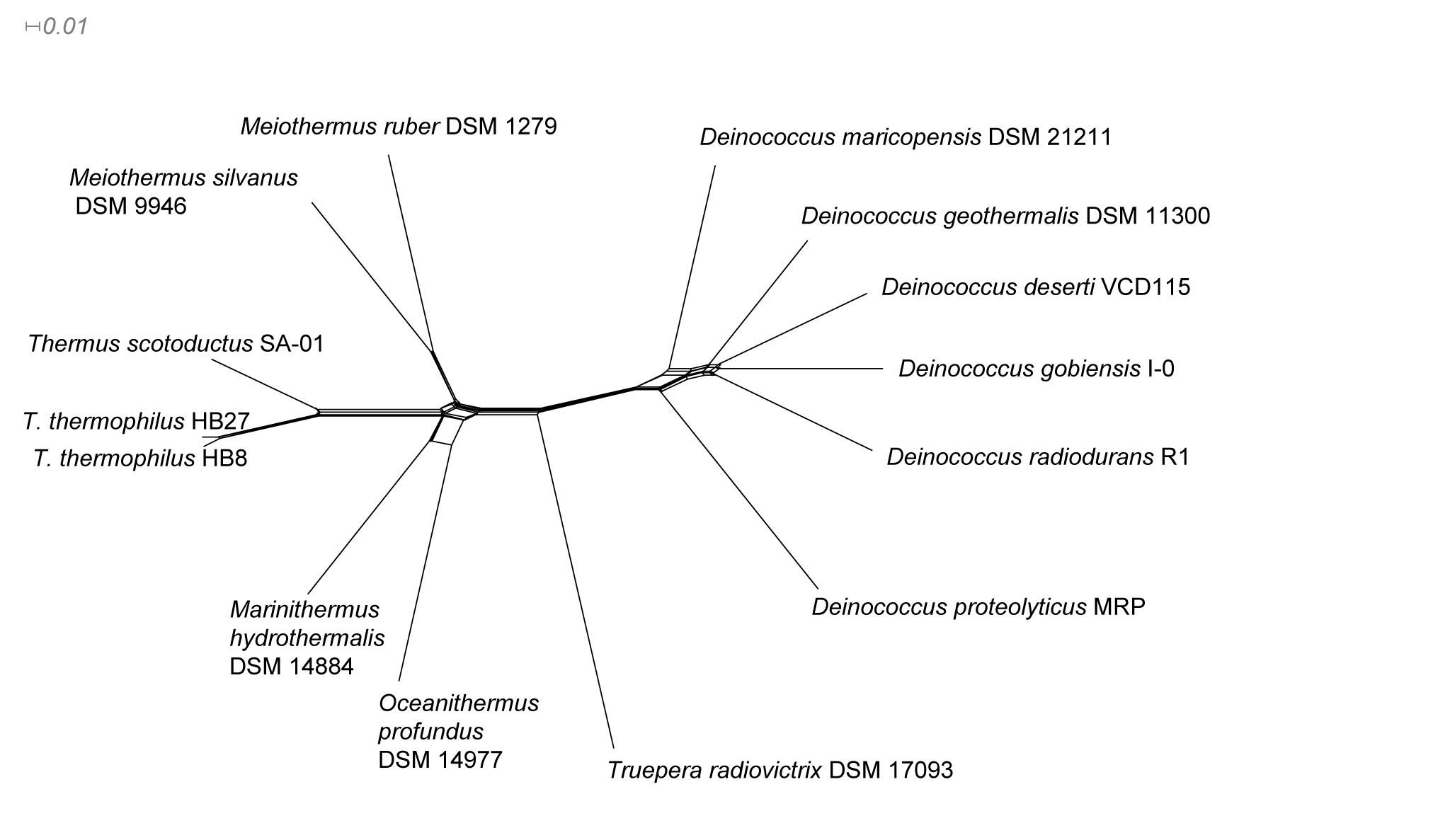

Supplement: Figure S1 — Unrooted neighbor-joining phylogenetic tree deduced from the orthologous proteins that occur in all 14 sequenced strains from the phylum Deinococcus-Thermus. D. gobiensis and D. radiodurans are most closely related. Numbers indicate bootstrap values below 100. (TIF) [file pone.0034458.s001.tif]

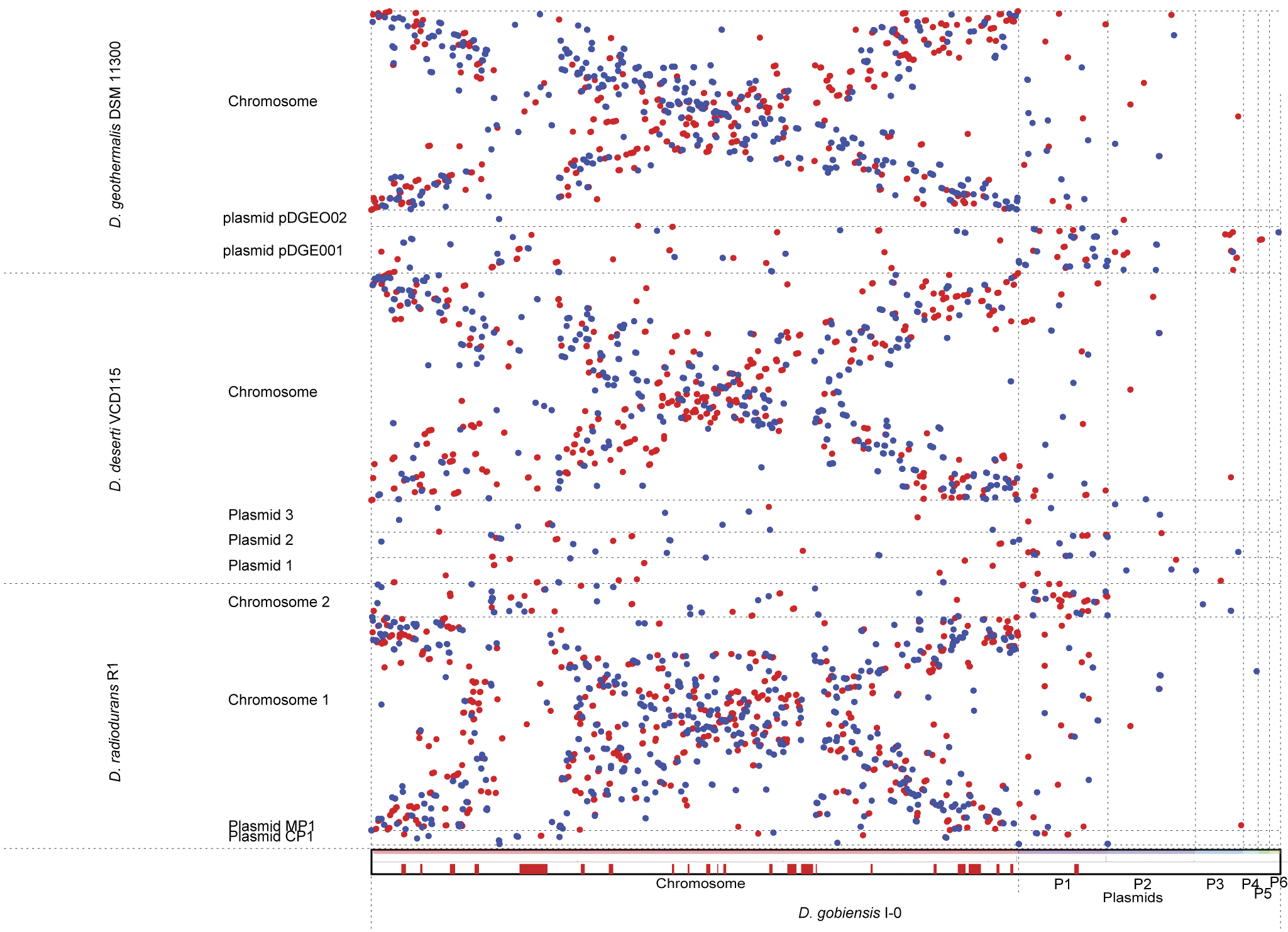

Supplement: Figure S2 — Synteny plots comparing D. gobiensis I-0 and the other three Deinococcus genomes. The dot plots represent nucmer alignments generated by MUMMER 3 of D. gobiensis on the x-axis and three other Deinococcus species on the y-axis. Forward matches are shown in red, and reverse matches are shown in blue. dnaA isat the bottom left of each plot. The red marks on the horizontal line representing the D. gobiensis genome indicate genomic islands. (TIF) [file pone.0034458.s002.tif]
