# Supplementary material for: Genome Sequence and Transcriptome Analysis of the Radioresistant Bacterium Deinococcus gobiensis: Insights into the Extreme Environmental Adaptations
Source: PLoS One. 2012 Mar 28;7(3):e34458. doi: 10.1371/journal.pone.0034458 (PMC3314630; doi:10.1371/journal.pone.0034458)
Supplement: Table S1 — General features of the genomes of Deinococcales species. (DOC) [file pone.0034458.s003.doc]

Table S1 General features of the genomes of Deinococcales species

|  | *D. gobiensis* I-0^T^ | *D. radiodurans* R1^T^ | *D. deserti* VCD 115^T^ | *D. geothermalis* DSM11300^T^ | *D. proteolyticus* MRP | *D. maricopensis* LB-34^T^ | *Truepera radiovictrix* RQ-24^T^ |
| --- | --- | --- | --- | --- | --- | --- | --- |
| Chromosome(s) | 1 | 2 | 1 | 1 | 1 | 1 | 1 |
| Plasmids | 6 | 2 | 3 | 2 | 4 | 0 | 0 |
| Size(bp) | 4,406,036 | 3,284,156 | 3,855,329 | 3,247,018 | 2,886,836 | 3,498,530 | 3,260,398 |
| GC content (%) | 69.15 | 66.6 | 62.96 | 66.47 | 65.65 | 69.83 | 68.14 |
| Coding Density (%) | 85.4 | 90.9 | 84.31 | 89.97 | (ND) | 89.38 | 87.79 |
| Protein-Coding genes | 4340 | 3187 | 3455 | 3062 | 2656 | 3,301 | 2994 |
| (Average length, nt) | (863) | (937) | (ND) | (954) | (ND) | (ND) | (ND) |
| Pseudo genes | 19 | ND | ND | ND | 83 | 37 | 49 |
| tRNAs | 46 | 49 | 48 | 48 | 47 | 54 | 46 |
| 5S rRNAs | 5 | 3 | 4 | 4 | 3 | 4 | 2 |
| 16S rRNAs | 5 | 3 | 4 | 4 | 3 | 4 | 2 |
| 23S rRNAs | 5 | 3 | 4 | 4 | 3 | 4 | 2 |
| non-coding RNAs | 45 | ND | 5 | ND | 2 | ND | ND |
