# Supplementary material for: Genome Sequence and Transcriptome Analysis of the Radioresistant Bacterium Deinococcus gobiensis: Insights into the Extreme Environmental Adaptations
Source: PLoS One. 2012 Mar 28;7(3):e34458. doi: 10.1371/journal.pone.0034458 (PMC3314630; doi:10.1371/journal.pone.0034458)
Supplement: Table S3 — Transcriptional start sites (TSS). First column presents the TSS location; the TSS and its strand are listed in the next two columns. (DOC) [file pone.0034458.s005.doc]

**Table S3. Transcriptional start sites (TSS).**

First column presents the TSS location; the TSS and its strand are listed in the next two columns.

| **Location** | **Site** | **Direction** |
| --- | --- | --- |
| **C1** | 1782 | + |
| **C1** | 3485 | + |
| **C1** | 14106 | - |
| **C1** | 15011 | - |
| **C1** | 16068 | - |
| **C1** | 17352 | - |
| **C1** | 19229 | - |
| **C1** | 19556 | + |
| **C1** | 35587 | - |
| **C1** | 43297 | - |
| **C1** | 43314 | + |
| **C1** | 44133 | + |
| **C1** | 50646 | + |
| **C1** | 51589 | + |
| **C1** | 52478 | + |
| **C1** | 70240 | - |
| **C1** | 76803 | - |
| **C1** | 76995 | + |
| **C1** | 84325 | + |
| **C1** | 88387 | + |
| **C1** | 96681 | + |
| **C1** | 98273 | - |
| **C1** | 100367 | + |
| **C1** | 363295 | + |
| **C1** | 366378 | + |
| **C1** | 114153 | + |
| **C1** | 131979 | + |
| **C1** | 138946 | - |
| **C1** | 139340 | + |
| **C1** | 146891 | + |
| **C1** | 149680 | - |
| **C1** | 151252 | - |
| **C1** | 170962 | + |
| **C1** | 180563 | + |
| **C1** | 189516 | - |
| **C1** | 189763 | + |
| **C1** | 192078 | + |
| **C1** | 202647 | - |
| **C1** | 205031 | + |
| **C1** | 208008 | - |
| **C1** | 212275 | + |
| **C1** | 212511 | + |
| **C1** | 214093 | - |
| **C1** | 216057 | - |
| **C1** | 219656 | - |
| **C1** | 220323 | - |
| **C1** | 221689 | - |
| **C1** | 230080 | + |
| **C1** | 230809 | + |
| **C1** | 231961 | - |
| **C1** | 248472 | + |
| **C1** | 250196 | - |
| **C1** | 258783 | + |
| **C1** | 274648 | + |
| **C1** | 275840 | + |
| **C1** | 279033 | - |
| **C1** | 295367 | + |
| **C1** | 298136 | + |
| **C1** | 300052 | + |
| **C1** | 310389 | - |
| **C1** | 314115 | - |
| **C1** | 315469 | + |
| **C1** | 321597 | - |
| **C1** | 321776 | + |
| **C1** | 323812 | - |
| **C1** | 325246 | - |
| **C1** | 337649 | - |
| **C1** | 338476 | - |
| **C1** | 344952 | + |
| **C1** | 345587 | + |
| **C1** | 349838 | + |
| **C1** | 350962 | + |
| **C1** | 357402 | + |
| **C1** | 361538 | + |
| **C1** | 364436 | + |
| **C1** | 365672 | + |
| **C1** | 369775 | - |
| **C1** | 375210 | + |
| **C1** | 375536 | + |
| **C1** | 376938 | + |
| **C1** | 378618 | + |
| **C1** | 394044 | - |
| **C1** | 407069 | + |
| **C1** | 407924 | + |
| **C1** | 410179 | + |
| **C1** | 412468 | + |
| **C1** | 413498 | + |
| **C1** | 416290 | + |
| **C1** | 420375 | + |
| **C1** | 423473 | + |
| **C1** | 426292 | + |
| **C1** | 431671 | - |
| **C1** | 447329 | + |
| **C1** | 450306 | - |
| **C1** | 451242 | - |
| **C1** | 452346 | - |
| **C1** | 452521 | + |
| **C1** | 456252 | - |
| **C1** | 459013 | - |
| **C1** | 461112 | + |
| **C1** | 471409 | + |
| **C1** | 473947 | - |
| **C1** | 479326 | + |
| **C1** | 484510 | + |
| **C1** | 488708 | + |
| **C1** | 490320 | + |
| **C1** | 493901 | + |
| **C1** | 495998 | + |
| **C1** | 499007 | + |
| **C1** | 499827 | + |
| **C1** | 500303 | + |
| **C1** | 506567 | - |
| **C1** | 509463 | + |
| **C1** | 511715 | - |
| **C1** | 511863 | + |
| **C1** | 512807 | - |
| **C1** | 514918 | + |
| **C1** | 529067 | + |
| **C1** | 535722 | - |
| **C1** | 536658 | - |
| **C1** | 549661 | + |
| **C1** | 550717 | - |
| **C1** | 572118 | + |
| **C1** | -460845 | - |
| **C1** | 578927 | - |
| **C1** | 583678 | - |
| **C1** | 589960 | - |
| **C1** | 626995 | - |
| **C1** | 631005 | - |
| **C1** | 633654 | - |
| **C1** | 643234 | - |
| **C1** | 643897 | - |
| **C1** | 646319 | - |
| **C1** | 646563 | + |
| **C1** | 656999 | + |
| **C1** | 662900 | + |
| **C1** | 679229 | - |
| **C1** | 682495 | + |
| **C1** | 699253 | - |
| **C1** | 703724 | - |
| **C1** | 719602 | + |
| **C1** | 727925 | + |
| **C1** | 729183 | + |
| **C1** | 732555 | + |
| **C1** | 738248 | + |
| **C1** | 752387 | - |
| **C1** | 754068 | + |
| **C1** | 754509 | - |
| **C1** | 767861 | + |
| **C1** | 796747 | - |
| **C1** | 800451 | - |
| **C1** | -460845 | + |
| **C1** | -363295 | + |
| **C1** | -366378 | + |
| **C1** | 889791 | + |
| **C1** | -366378 | - |
| **C1** | -363295 | - |
| **C1** | -460845 | - |
| **C1** | 914436 | - |
| **C1** | 914735 | - |
| **C1** | 917635 | + |
| **C1** | 918786 | + |
| **C1** | 919244 | + |
| **C1** | 921485 | + |
| **C1** | 921964 | + |
| **C1** | 924151 | + |
| **C1** | 929522 | - |
| **C1** | 930910 | - |
| **C1** | 937582 | - |
| **C1** | 940627 | - |
| **C1** | 943013 | - |
| **C1** | 945820 | - |
| **C1** | 948089 | - |
| **C1** | 948288 | + |
| **C1** | 956254 | + |
| **C1** | 957226 | - |
| **C1** | 957798 | + |
| **C1** | 970236 | - |
| **C1** | 974900 | + |
| **C1** | 981236 | + |
| **C1** | 983094 | + |
| **C1** | 986679 | + |
| **C1** | 987445 | + |
| **C1** | 992635 | + |
| **C1** | 994338 | + |
| **C1** | 1006742 | - |
| **C1** | 1009222 | + |
| **C1** | 1011744 | + |
| **C1** | 1013980 | + |
| **C1** | 1023711 | - |
| **C1** | 1024486 | - |
| **C1** | 1024997 | - |
| **C1** | 1026002 | - |
| **C1** | 1028707 | - |
| **C1** | 1028888 | + |
| **C1** | 1030233 | + |
| **C1** | 1043132 | + |
| **C1** | 1050891 | - |
| **C1** | 1070067 | + |
| **C1** | 1081224 | - |
| **C1** | 1084031 | + |
| **C1** | 1085124 | - |
| **C1** | 1088774 | - |
| **C1** | 1093141 | + |
| **C1** | 1095310 | - |
| **C1** | 1102421 | + |
| **C1** | 1104470 | - |
| **C1** | 1108749 | + |
| **C1** | 1110036 | + |
| **C1** | 1110717 | - |
| **C1** | 1115334 | + |
| **C1** | 1124282 | - |
| **C1** | 1124774 | - |
| **C1** | 1125037 | + |
| **C1** | 1138883 | + |
| **C1** | 1143026 | - |
| **C1** | 1145814 | - |
| **C1** | 1146008 | + |
| **C1** | 1149842 | + |
| **C1** | 1163094 | - |
| **C1** | 1164096 | - |
| **C1** | 1174515 | + |
| **C1** | 1181485 | + |
| **C1** | 1182799 | + |
| **C1** | 1183432 | + |
| **C1** | 1188611 | + |
| **C1** | 1195402 | + |
| **C1** | 1198361 | + |
| **C1** | 1203294 | + |
| **C1** | 1207210 | - |
| **C1** | 1214119 | - |
| **C1** | 1218890 | - |
| **C1** | 1220000 | + |
| **C1** | 1223624 | - |
| **C1** | 1225266 | + |
| **C1** | 1225762 | + |
| **C1** | 1230981 | - |
| **C1** | 1232237 | + |
| **C1** | 1244296 | - |
| **C1** | 1245663 | - |
| **C1** | 1246855 | - |
| **C1** | 1248235 | - |
| **C1** | 1248871 | - |
| **C1** | 1249103 | - |
| **C1** | 1249352 | - |
| **C1** | 1251920 | - |
| **C1** | 1265485 | - |
| **C1** | 1265589 | + |
| **C1** | 1271666 | + |
| **C1** | 1271977 | + |
| **C1** | 1286738 | - |
| **C1** | 1299392 | - |
| **C1** | 1307758 | - |
| **C1** | 1311162 | + |
| **C1** | 1313054 | + |
| **C1** | 1314393 | - |
| **C1** | 1316158 | + |
| **C1** | 1317361 | - |
| **C1** | 1331362 | - |
| **C1** | 1336959 | - |
| **C1** | 1349169 | + |
| **C1** | 1354528 | - |
| **C1** | 1357968 | + |
| **C1** | 1359008 | + |
| **C1** | 1380135 | - |
| **C1** | 1388353 | + |
| **C1** | 1390195 | - |
| **C1** | 1401032 | + |
| **C1** | 1406080 | - |
| **C1** | 1412083 | - |
| **C1** | 1418028 | + |
| **C1** | 1418938 | + |
| **C1** | 1422143 | - |
| **C1** | 1424686 | - |
| **C1** | 1428025 | + |
| **C1** | 1435199 | + |
| **C1** | 1442749 | - |
| **C1** | 1444755 | + |
| **C1** | 1452157 | - |
| **C1** | 1456004 | + |
| **C1** | 1457361 | + |
| **C1** | 1458277 | + |
| **C1** | 1470728 | + |
| **C1** | 1474359 | - |
| **C1** | 1477005 | + |
| **C1** | 1481747 | + |
| **C1** | 1491108 | + |
| **C1** | 1493034 | + |
| **C1** | 1493337 | + |
| **C1** | 1497278 | + |
| **C1** | 1501068 | + |
| **C1** | 1509501 | + |
| **C1** | 1510850 | + |
| **C1** | 1511700 | + |
| **C1** | 1514947 | - |
| **C1** | 1515783 | - |
| **C1** | 1524829 | + |
| **C1** | 1528341 | - |
| **C1** | 1529263 | + |
| **C1** | 1542402 | + |
| **C1** | 1545044 | + |
| **C1** | 1547069 | - |
| **C1** | 1549239 | - |
| **C1** | 1559879 | - |
| **C1** | 1564598 | - |
| **C1** | 1565265 | - |
| **C1** | 1570537 | - |
| **C1** | 1576372 | + |
| **C1** | 1581462 | - |
| **C1** | 1583397 | - |
| **C1** | 1583859 | - |
| **C1** | 1586336 | + |
| **C1** | 1593533 | - |
| **C1** | 1597463 | + |
| **C1** | 1608621 | - |
| **C1** | 1609440 | + |
| **C1** | 1615775 | - |
| **C1** | 1628587 | - |
| **C1** | 1634784 | - |
| **C1** | 1635236 | - |
| **C1** | 1635329 | + |
| **C1** | 1647194 | - |
| **C1** | 1648095 | - |
| **C1** | 1654224 | - |
| **C1** | 1668553 | + |
| **C1** | 1670130 | + |
| **C1** | 1671173 | - |
| **C1** | 1671777 | - |
| **C1** | 1681348 | + |
| **C1** | 1684114 | - |
| **C1** | 1687659 | - |
| **C1** | 1688787 | - |
| **C1** | 1692742 | + |
| **C1** | 1697337 | - |
| **C1** | 1705211 | + |
| **C1** | 1707306 | - |
| **C1** | 1709133 | + |
| **C1** | 1714639 | + |
| **C1** | 1725827 | - |
| **C1** | 1728058 | - |
| **C1** | 1729700 | + |
| **C1** | 1740557 | + |
| **C1** | 1751823 | - |
| **C1** | 1758070 | - |
| **C1** | 1763117 | - |
| **C1** | 1775814 | + |
| **C1** | 1780398 | - |
| **C1** | 1783585 | + |
| **C1** | 1790377 | - |
| **C1** | 1798239 | - |
| **C1** | 1820392 | - |
| **C1** | 1821173 | - |
| **C1** | 1824224 | - |
| **C1** | 1824386 | + |
| **C1** | 1831019 | - |
| **C1** | 1831657 | - |
| **C1** | 1832350 | - |
| **C1** | 1847329 | + |
| **C1** | 1848999 | + |
| **C1** | 1849964 | + |
| **C1** | 1854462 | + |
| **C1** | 1857778 | - |
| **C1** | 1858942 | + |
| **C1** | 1860483 | - |
| **C1** | 1867066 | + |
| **C1** | 1867429 | + |
| **C1** | 1871822 | - |
| **C1** | 1878412 | + |
| **C1** | 1884083 | + |
| **C1** | 1896996 | + |
| **C1** | 1898059 | - |
| **C1** | 1903596 | - |
| **C1** | 1905343 | + |
| **C1** | 1922013 | - |
| **C1** | 1932386 | - |
| **C1** | 1933883 | - |
| **C1** | 1943580 | - |
| **C1** | 1950657 | + |
| **C1** | 1953970 | - |
| **C1** | 1959739 | - |
| **C1** | 1959969 | + |
| **C1** | 1962347 | + |
| **C1** | 1974328 | + |
| **C1** | 1985054 | - |
| **C1** | 1987039 | - |
| **C1** | 1987406 | + |
| **C1** | 1988432 | + |
| **C1** | 1989350 | + |
| **C1** | 1990099 | + |
| **C1** | 1999428 | + |
| **C1** | 2001373 | - |
| **C1** | 2001629 | + |
| **C1** | 2011960 | - |
| **C1** | 2012665 | - |
| **C1** | 2012882 | + |
| **C1** | 2019846 | - |
| **C1** | 2020304 | + |
| **C1** | 2041111 | + |
| **C1** | 2071146 | - |
| **C1** | 2072608 | + |
| **C1** | 2077489 | + |
| **C1** | 2079157 | + |
| **C1** | 2080659 | + |
| **C1** | 2082167 | + |
| **C1** | 2083316 | + |
| **C1** | 2086728 | - |
| **C1** | 2099106 | - |
| **C1** | 2155850 | + |
| **C1** | 2160068 | + |
| **C1** | 2164619 | + |
| **C1** | 2166350 | - |
| **C1** | 2167916 | - |
| **C1** | 2170013 | - |
| **C1** | 2178146 | - |
| **C1** | 2178631 | - |
| **C1** | 2183403 | + |
| **C1** | 2185041 | + |
| **C1** | 2191582 | - |
| **C1** | 2194870 | + |
| **C1** | 2198071 | - |
| **C1** | 2202084 | - |
| **C1** | 2206316 | - |
| **C1** | 2207153 | - |
| **C1** | 2207366 | + |
| **C1** | 2209589 | + |
| **C1** | 2214001 | + |
| **C1** | 2214179 | + |
| **C1** | 2218459 | - |
| **C1** | 2219492 | + |
| **C1** | 2221417 | + |
| **C1** | 2222460 | + |
| **C1** | 2223477 | + |
| **C1** | 2227643 | - |
| **C1** | 2228243 | - |
| **C1** | 2229297 | + |
| **C1** | 2237082 | - |
| **C1** | 2240926 | + |
| **C1** | 2252823 | - |
| **C1** | 2261275 | - |
| **C1** | 2266230 | - |
| **C1** | 2267753 | - |
| **C1** | 2269368 | - |
| **C1** | 2270138 | - |
| **C1** | 2270361 | + |
| **C1** | 2271545 | - |
| **C1** | 2274000 | - |
| **C1** | 2281478 | + |
| **C1** | 2284355 | - |
| **C1** | 2287436 | - |
| **C1** | 2290237 | - |
| **C1** | 2292279 | - |
| **C1** | 2293807 | - |
| **C1** | 2302857 | + |
| **C1** | 2313036 | - |
| **C1** | 2320575 | + |
| **C1** | 2338154 | - |
| **C1** | 2339664 | - |
| **C1** | 2343042 | - |
| **C1** | 2344133 | - |
| **C1** | 2345549 | - |
| **C1** | 2346614 | - |
| **C1** | 2346967 | + |
| **C1** | 2348752 | + |
| **C1** | 2363009 | - |
| **C1** | 2364766 | - |
| **C1** | 2376817 | - |
| **C1** | 2381130 | + |
| **C1** | 2383793 | - |
| **C1** | 2388844 | - |
| **C1** | 2401734 | + |
| **C1** | 2405252 | - |
| **C1** | 2406757 | + |
| **C1** | 2415684 | + |
| **C1** | 2425071 | + |
| **C1** | 2428053 | - |
| **C1** | 2431633 | + |
| **C1** | 2439693 | + |
| **C1** | 2444589 | + |
| **C1** | 2446975 | - |
| **C1** | 2449101 | + |
| **C1** | 2454968 | + |
| **C1** | 2456204 | + |
| **C1** | 2456657 | - |
| **C1** | 2457934 | + |
| **C1** | 2460574 | + |
| **C1** | 2466020 | + |
| **C1** | 2468754 | + |
| **C1** | 2475654 | - |
| **C1** | 2477522 | - |
| **C1** | 2479343 | - |
| **C1** | 2486655 | - |
| **C1** | 2487624 | - |
| **C1** | 2488106 | + |
| **C1** | 2493079 | - |
| **C1** | 2494217 | - |
| **C1** | 2495047 | - |
| **C1** | 2497817 | - |
| **C1** | 2499665 | - |
| **C1** | 2500032 | + |
| **C1** | 2500363 | + |
| **C1** | 2507884 | + |
| **C1** | 2512529 | - |
| **C1** | 2515100 | - |
| **C1** | 2516819 | + |
| **C1** | 2518194 | + |
| **C1** | 2525759 | - |
| **C1** | 2527964 | - |
| **C1** | 2529868 | - |
| **C1** | 2538428 | - |
| **C1** | 2540147 | - |
| **C1** | 2540374 | + |
| **C1** | 2542850 | + |
| **C1** | 2543381 | + |
| **C1** | 2556704 | - |
| **C1** | 2560268 | - |
| **C1** | 2565451 | - |
| **C1** | 2566529 | + |
| **C1** | 2573070 | - |
| **C1** | 2579377 | - |
| **C1** | 2580023 | - |
| **C1** | 2580425 | + |
| **C1** | 2589456 | - |
| **C1** | 2589947 | - |
| **C1** | 2594722 | - |
| **C1** | 2594817 | - |
| **C1** | 2594939 | + |
| **C1** | 2599621 | + |
| **C1** | 2626224 | - |
| **C1** | 2628994 | - |
| **C1** | 2629844 | + |
| **C1** | 2634462 | - |
| **C1** | 2637552 | - |
| **C1** | 2637797 | + |
| **C1** | 2640731 | - |
| **C1** | 2641796 | - |
| **C1** | 2643220 | + |
| **C1** | 2645959 | + |
| **C1** | 2646995 | - |
| **C1** | 2647119 | + |
| **C1** | 2648215 | + |
| **C1** | 2648920 | + |
| **C1** | 2650769 | + |
| **C1** | 2659887 | + |
| **C1** | 2662244 | - |
| **C1** | 2669473 | - |
| **C1** | 2676302 | + |
| **C1** | 2680279 | + |
| **C1** | 2682298 | + |
| **C1** | 2686797 | + |
| **C1** | 2693444 | - |
| **C1** | 2693628 | + |
| **C1** | 2694186 | + |
| **C1** | 2705152 | - |
| **C1** | 2712698 | - |
| **C1** | 2718240 | - |
| **C1** | 2718611 | - |
| **C1** | 2718727 | + |
| **C1** | 2720048 | + |
| **C1** | 2724231 | - |
| **C1** | 2727150 | - |
| **C1** | 2730817 | - |
| **C1** | 2731525 | - |
| **C1** | 2732993 | - |
| **C1** | 2746457 | - |
| **C1** | 2747209 | - |
| **C1** | 2747729 | - |
| **C1** | 2750441 | + |
| **C1** | 2759243 | - |
| **C1** | 2760544 | - |
| **C1** | 2761554 | - |
| **C1** | 2761673 | + |
| **C1** | 2764876 | - |
| **C1** | 2766497 | + |
| **C1** | 2769169 | + |
| **C1** | 2770282 | - |
| **C1** | 2770769 | - |
| **C1** | 2773852 | - |
| **C1** | 2775720 | - |
| **C1** | 2777951 | - |
| **C1** | 2779212 | - |
| **C1** | 2784376 | + |
| **C1** | 2792205 | - |
| **C1** | 2792453 | + |
| **C1** | 2793695 | - |
| **C1** | 2798236 | - |
| **C1** | 2804057 | + |
| **C1** | 2806061 | + |
| **C1** | 2814016 | - |
| **C1** | 2816052 | + |
| **C1** | 2816807 | - |
| **C1** | 2821462 | - |
| **C1** | 2821916 | - |
| **C1** | 2841470 | + |
| **C1** | 2848575 | + |
| **C1** | 2866154 | + |
| **C1** | 2867826 | - |
| **C1** | 2869950 | - |
| **C1** | 2878678 | + |
| **C1** | 2882129 | - |
| **C1** | 2895823 | + |
| **C1** | 2901001 | - |
| **C1** | 2902676 | - |
| **C1** | 2903638 | - |
| **C1** | 2917767 | - |
| **C1** | 2917856 | + |
| **C1** | 2956089 | + |
| **C1** | 2957327 | + |
| **C1** | 2958208 | + |
| **C1** | 2963019 | + |
| **C1** | 2974507 | + |
| **C1** | 2770769 | - |
| **C1** | 2773852 | - |
| **C1** | 2978352 | + |
| **C1** | 2676302 | - |
| **C1** | 2984898 | - |
| **C1** | 2985800 | - |
| **C1** | 2986362 | - |
| **C1** | 2992177 | + |
| **C1** | 3009084 | + |
| **C1** | 3014028 | + |
| **C1** | 3015312 | + |
| **C1** | 3016313 | + |
| **C1** | 3017339 | + |
| **C1** | 3023989 | - |
| **C1** | 3030988 | + |
| **C1** | 3046825 | - |
| **C1** | 3060313 | - |
| **C1** | 3065000 | + |
| **C1** | 3070963 | + |
| **C1** | 3112846 | + |
| **C1** | 3118389 | - |
| **C1** | 3120844 | - |
| **C1** | 3122873 | - |
| **C1** | 3124261 | + |
| **C1** | 3132458 | + |
| **C1** | 3133031 | + |
| **C1** | 3133766 | + |
| **C1** | 3136635 | - |
| **P1** | 2968 | - |
| **P1** | 3580 | - |
| **P1** | 3801 | + |
| **P1** | 7230 | - |
| **P1** | 7315 | + |
| **P1** | 8374 | + |
| **P1** | 9491 | + |
| **P1** | 13183 | + |
| **P1** | 18023 | + |
| **P1** | 23543 | + |
| **P1** | 25120 | + |
| **P1** | 46523 | + |
| **P1** | 47508 | + |
| **P1** | 48547 | + |
| **P1** | 61102 | - |
| **P1** | 61528 | + |
| **P1** | 68041 | + |
| **P1** | 69232 | + |
| **P1** | 69648 | + |
| **P1** | 75242 | - |
| **P1** | 76266 | + |
| **P1** | 80388 | + |
| **P1** | 82804 | + |
| **P1** | 87977 | + |
| **P1** | 90533 | - |
| **P1** | 93442 | - |
| **P1** | 93632 | + |
| **P1** | 94815 | + |
| **P1** | 97201 | + |
| **P1** | 100061 | + |
| **P1** | 100642 | + |
| **P1** | 101744 | + |
| **P1** | 112352 | - |
| **P1** | 114166 | - |
| **P1** | 119257 | - |
| **P1** | 119908 | + |
| **P1** | 123857 | - |
| **P1** | 131454 | - |
| **P1** | 131738 | + |
| **P1** | 133444 | + |
| **P1** | 137394 | + |
| **P1** | 140596 | + |
| **P1** | 142105 | - |
| **P1** | 148268 | - |
| **P1** | 148570 | + |
| **P1** | 148793 | + |
| **P1** | 149725 | + |
| **P1** | 157245 | + |
| **P1** | 168605 | - |
| **P1** | 171405 | + |
| **P1** | 173827 | + |
| **P1** | 177026 | - |
| **P1** | 184254 | - |
| **P1** | 191620 | + |
| **P1** | 199109 | - |
| **P1** | 201057 | - |
| **P1** | 204081 | - |
| **P1** | 205448 | + |
| **P1** | 206517 | + |
| **P1** | 208773 | - |
| **P1** | 208775 | + |
| **P1** | 214440 | - |
| **P1** | 216611 | - |
| **P1** | 226386 | - |
| **P1** | 229872 | - |
| **P1** | 231323 | - |
| **P1** | 232455 | + |
| **P1** | 236649 | + |
| **P1** | 237274 | + |
| **P1** | 239537 | + |
| **P1** | 241643 | - |
| **P1** | 243576 | - |
| **P1** | 246222 | - |
| **P1** | 246282 | + |
| **P1** | 249532 | - |
| **P1** | 251829 | + |
| **P1** | 252944 | + |
| **P1** | 253706 | + |
| **P1** | 255933 | + |
| **P1** | 257577 | + |
| **P1** | 258029 | + |
| **P1** | 261202 | + |
| **P1** | 261828 | + |
| **P1** | 262967 | + |
| **P1** | 264820 | + |
| **P1** | 266708 | + |
| **P1** | 268394 | + |
| **P1** | 275746 | - |
| **P1** | 276606 | - |
| **P1** | 279225 | - |
| **P1** | 281800 | - |
| **P1** | 285036 | + |
| **P1** | 286769 | + |
| **P1** | 292505 | - |
| **P1** | 297598 | - |
| **P1** | 300384 | - |
| **P1** | 300637 | + |
| **P1** | 303543 | + |
| **P1** | 308076 | - |
| **P1** | 308332 | + |
| **P1** | 311693 | + |
| **P1** | 315319 | - |
| **P1** | 320023 | - |
| **P1** | 323997 | - |
| **P1** | 327317 | - |
| **P1** | 330550 | - |
| **P1** | 331638 | - |
| **P1** | 333358 | - |
| **P1** | 334623 | - |
| **P1** | 337171 | - |
| **P1** | 339167 | - |
| **P1** | 341544 | - |
| **P1** | 341943 | + |
| **P1** | 344217 | - |
| **P1** | 346455 | + |
| **P1** | 355800 | + |
| **P1** | 358020 | - |
| **P1** | 358629 | - |
| **P1** | 360305 | - |
| **P1** | 364628 | + |
| **P1** | 366107 | + |
| **P1** | 367125 | + |
| **P1** | 368248 | + |
| **P1** | 369023 | + |
| **P1** | 369783 | + |
| **P1** | 371333 | - |
| **P1** | 373226 | + |
| **P1** | 380326 | - |
| **P1** | 381173 | - |
| **P1** | 387652 | - |
| **P1** | 388248 | - |
| **P1** | 389520 | - |
| **P1** | 392365 | - |
| **P1** | 393417 | - |
| **P1** | 394273 | - |
| **P1** | 394683 | - |
| **P1** | 396819 | - |
| **P1** | 396827 | + |
| **P1** | 399398 | + |
| **P1** | 405088 | + |
| **P1** | 420059 | - |
| **P1** | 420302 | + |
| **P1** | 422753 | - |
| **P1** | 424724 | - |
| **P1** | 424772 | + |
| **P1** | 426423 | + |
| **P2** | 3719 | + |
| **P2** | 4118 | + |
| **P2** | 19868 | + |
| **P2** | 20471 | - |
| **P2** | 28402 | - |
| **P2** | 30314 | - |
| **P2** | 35964 | + |
| **P2** | 41271 | - |
| **P2** | 47129 | + |
| **P2** | 47961 | + |
| **P2** | 50866 | - |
| **P2** | 51228 | + |
| **P2** | 56334 | + |
| **P2** | 82028 | + |
| **P2** | 83214 | + |
| **P2** | 85152 | - |
| **P2** | 87917 | - |
| **P2** | 90091 | + |
| **P2** | 90878 | + |
| **P2** | 91328 | + |
| **P2** | 96467 | - |
| **P2** | 102335 | - |
| **P2** | 112047 | - |
| **P2** | 114949 | + |
| **P2** | 121898 | + |
| **P2** | 143419 | - |
| **P2** | 143862 | + |
| **P2** | 147527 | - |
| **P2** | 150897 | - |
| **P2** | 153479 | - |
| **P2** | 153693 | + |
| **P2** | 159473 | - |
| **P2** | 162351 | + |
| **P2** | 164532 | - |
| **P2** | 166138 | + |
| **P2** | 168126 | - |
| **P2** | 174783 | + |
| **P2** | 183581 | + |
| **P2** | 220074 | + |
| **P2** | 220587 | + |
| **P2** | 226131 | - |
| **P2** | 238337 | + |
| **P2** | 242035 | - |
| **P2** | 245808 | + |
| **P2** | 252129 | - |
| **P2** | 254743 | - |
| **P2** | 259205 | + |
| **P2** | 267390 | + |
| **P2** | 277279 | - |
| **P2** | 278075 | - |
| **P2** | 281688 | - |
| **P2** | 285150 | + |
| **P2** | 286225 | - |
| **P2** | 286546 | - |
| **P2** | 286975 | - |
| **P2** | 288627 | - |
| **P2** | 300386 | + |
| **P2** | 301342 | + |
| **P2** | 312251 | - |
| **P2** | 316853 | + |
| **P2** | 321006 | + |
| **P2** | 321764 | + |
| **P2** | 326710 | - |
| **P2** | 327688 | - |
| **P2** | 329386 | - |
| **P2** | 329529 | + |
| **P2** | 334526 | + |
| **P2** | 336202 | - |
| **P2** | 340882 | + |
| **P2** | 341720 | + |
| **P2** | 352933 | + |
| **P2** | 353379 | + |
| **P2** | 359561 | + |
| **P2** | 360702 | - |
| **P2** | 360841 | + |
| **P2** | 379771 | - |
| **P2** | 395451 | - |
| **P2** | 405246 | + |
| **P2** | 407265 | + |
| **P2** | 408769 | + |
| **P2** | 410020 | - |
| **P2** | 410394 | - |
| **P2** | 410767 | - |
| **P2** | 410885 | + |
| **P2** | 413015 | + |
| **P2** | 414236 | + |
| **P2** | 415395 | - |
| **P2** | 424110 | - |
| **P3** | 9961 | + |
| **P3** | 15206 | - |
| **P3** | 22580 | + |
| **P3** | 36855 | - |
| **P3** | 45538 | - |
| **P3** | 48244 | + |
| **P3** | 49016 | + |
| **P3** | 52987 | - |
| **P3** | 53603 | - |
| **P3** | 53844 | + |
| **P3** | 57179 | - |
| **P3** | 62541 | + |
| **P3** | 89372 | + |
| **P3** | 90856 | - |
| **P3** | 91507 | - |
| **P3** | 94843 | + |
| **P3** | 104204 | + |
| **P3** | 115684 | + |
| **P3** | 123821 | - |
| **P3** | 127880 | + |
| **P3** | 136918 | + |
| **P3** | 141625 | + |
| **P3** | 146407 | + |
| **P3** | 149058 | - |
| **P3** | 151486 | - |
| **P3** | 152415 | - |
| **P3** | 153539 | + |
| **P3** | 169867 | - |
| **P3** | 175240 | + |
| **P3** | 175921 | + |
| **P3** | 178187 | + |
| **P3** | 178615 | + |
| **P3** | 196395 | + |
| **P3** | 197713 | - |
| **P3** | 197779 | + |
| **P3** | 200229 | + |
| **P3** | 204099 | - |
| **P3** | 204893 | + |
| **P3** | 206348 | - |
| **P3** | 206604 | + |
| **P3** | 207141 | + |
| **P3** | 208691 | - |
| **P3** | 209649 | - |
| **P3** | 212922 | - |
| **P3** | 215714 | + |
| **P3** | 217328 | + |
| **P3** | 222519 | - |
| **P4** | 1070 | + |
| **P4** | 1891 | + |
| **P4** | 2870 | - |
| **P4** | 3331 | + |
| **P4** | 4219 | + |
| **P4** | 5020 | + |
| **P4** | 6760 | - |
| **P4** | 12792 | + |
| **P4** | 20894 | - |
| **P4** | 22410 | - |
| **P4** | 22564 | + |
| **P4** | 24275 | + |
| **P4** | 24774 | - |
| **P4** | 25373 | - |
| **P4** | 30387 | - |
| **P4** | 32174 | + |
| **P4** | 33096 | + |
| **P4** | 39833 | + |
| **P4** | 42689 | + |
| **P4** | 45417 | - |
| **P4** | 45670 | + |
| **P4** | 49464 | - |
| **P4** | 50003 | - |
| **P4** | 52898 | - |
| **P4** | 55988 | - |
| **P4** | 62203 | + |
| **P4** | 64211 | + |
| **P4** | 69555 | - |
| **P5** | 1772 | - |
| **P5** | 2958 | + |
| **P5** | 3717 | + |
| **P5** | 4090 | + |
| **P5** | 4447 | + |
| **P5** | 5435 | + |
| **P5** | 14894 | - |
| **P5** | 16205 | - |
| **P5** | 17172 | - |
| **P5** | 17279 | + |
| **P5** | 22368 | - |
| **P5** | 23221 | - |
| **P5** | 25375 | - |
| **P5** | 26351 | + |
| **P5** | 28326 | + |
| **P5** | 30284 | - |
| **P5** | 30423 | + |
| **P5** | 30841 | + |
| **P5** | 31703 | - |
| **P5** | 31710 | + |
| **P5** | 32956 | - |
| **P5** | 33709 | - |
| **P5** | 34514 | - |
| **P5** | 36186 | - |
| **P5** | 36455 | + |
| **P5** | 36762 | + |
| **P5** | 39330 | - |
| **P5** | 39441 | + |
| **P5** | 40887 | + |
| **P5** | 43256 | + |
| **P5** | 43964 | + |
| **P5** | 45228 | - |
| **P5** | 45585 | + |
| **P5** | 46975 | + |
| **P5** | 47458 | + |
| **P5** | 50498 | - |
| **P5** | 51383 | - |
| **P5** | 51437 | + |
| **P5** | 53440 | + |
| **P6** | 12801 | - |
| **P6** | 22209 | + |
| **P6** | 34362 | + |
| **P6** | 38612 | - |
| **P6** | 40336 | - |
| **P6** | 44673 | - |
| **P6** | 44815 | + |
| **P6** | 47526 | - |
| **P6** | 48974 | + |
| **P6** | 50272 | - |
