# Supplementary material for: Genome Sequence and Transcriptome Analysis of the Radioresistant Bacterium Deinococcus gobiensis: Insights into the Extreme Environmental Adaptations
Source: PLoS One. 2012 Mar 28;7(3):e34458. doi: 10.1371/journal.pone.0034458 (PMC3314630; doi:10.1371/journal.pone.0034458)
Supplement: Table S4 — Functional description of 1144 genes induced or repressed after UV-irradiation. (DOC) [file pone.0034458.s006.doc]

**Table S4** Functional description of 1144 genes induced or repressed after UV-irradiation

| **A. Functional description of 390 genes induced after UV-irradiation** | | | | | |
| --- | --- | --- | --- | --- | --- |
| **Locus_tag** | **Type** | | **Name** | **Description** | **Fold** |
| **DGo_PFnc06** | ncRNA | |  | Hypothetical non-coding RNA | >100.0 |
| **DGo_CAnc05** | ncRNA | |  | Hypothetical non-coding RNA | 150.2 |
| **DGo_CAnc12** | ncRNA | |  | Hypothetical non-coding RNA | 57.3 |
| **DGo_CAnc03** | ncRNA | |  | Hypothetical non-coding RNA | 20.1 |
| **DGo_PBnc03** | ncRNA | |  | Hypothetical non-coding RNA | 13.4 |
| **DGo_CAnc04** | ncRNA | |  | Hypothetical non-coding RNA | 12.4 |
| **DGo_CAnc13** | ncRNA | |  | Hypothetical non-coding RNA | 10.5 |
| **DGo_PAnc07** | ncRNA | |  | Hypothetical non-coding RNA | 6.9 |
| **DGo_PFnc05** | ncRNA | |  | Hypothetical non-coding RNA | 5.6 |
| **DGo_PDnc01** | ncRNA | |  | Hypothetical non-coding RNA | 4.1 |
| **DGo_PAnc01** | ncRNA | |  | Hypothetical non-coding RNA | 3.8 |
| **DGo_PAnc05** | ncRNA | |  | Hypothetical non-coding RNA | 3.0 |
| **DGo_CAnc15** | ncRNA | |  | Hypothetical non-coding RNA | 2.2 |
| **DGo_CA1805** | CDS | |  | Hypothetical protein | >100.0 |
| **DGo_PB0291** | CDS | |  | Hypothetical protein | >100.0 |
| **DGo_PB0445** | CDS | |  | Hypothetical protein | 25.6 |
| **DGo_CA0814** | CDS | |  | Hypothetical protein | 22.5 |
| **DGo_CA2675** | CDS | |  | Hypothetical protein | 20.4 |
| **DGo_PB0147** | CDS | |  | Hypothetical protein | 14.3 |
| **DGo_PB0313** | CDS | |  | Hypothetical protein | 14.3 |
| **DGo_PB0065** | CDS | |  | Hypothetical protein | 13.7 |
| **DGo_PB0263** | CDS | |  | Hypothetical protein | 13.4 |
| **DGo_CA0175** | CDS | |  | Hypothetical protein | 13.3 |
| **DGo_PC0048** | CDS | |  | Hypothetical protein | 11.5 |
| **DGo_PB0290** | CDS | |  | Hypothetical protein | 11.0 |
| **DGo_CA2520** | CDS | |  | Acetyltransferase, putative | 10.2 |
| **DGo_PC0075** | CDS | |  | Methyltransferase type 11 | 8.7 |
| **DGo_PB0155** | CDS | |  | Hypothetical protein | 8.4 |
| **DGo_PB0406** | CDS | |  | Transposase, IS5 family | 8.3 |
| **DGo_PB0487** | CDS | |  | Hypothetical protein | 8.3 |
| **DGo_PA0146** | CDS | |  | Hypothetical protein | 8.0 |
| **DGo_PB0359** | CDS | |  | Hypothetical protein | 8.0 |
| **DGo_PB0151** | CDS | |  | Hypothetical protein | 8.0 |
| **DGo_PD0014** | CDS | |  | Hypothetical protein | 7.9 |
| **DGo_PD0052** | CDS | |  | Hypothetical protein | 7.9 |
| **DGo_PF0041** | CDS | |  | Hypothetical protein | 7.8 |
| **DGo_PC0253** | CDS | |  | Transposase, IS4 | 7.6 |
| **DGo_PD0062** | CDS | | *vapB* | SpoVT/AbrB-like | 7.5 |
| **DGo_PB0405** | CDS | |  | IS5 family transposase, orfA | 7.5 |
| **DGo_CA1616** | CDS | |  | Hypothetical protein | 7.4 |
| **DGo_CA0594** | CDS | |  | Hypothetical protein | 7.4 |
| **DGo_PC0035** | CDS | |  | Hypothetical protein | 7.4 |
| **DGo_PD0024** | CDS | |  | Hypothetical protein | 7.1 |
| **DGo_PD0018** | CDS | |  | Hypothetical protein | 7.1 |
| **DGo_CA0929** | CDS | |  | Hypothetical protein | 6.8 |
| **DGo_PB0171** | CDS | |  | Hypothetical protein | 6.8 |
| **DGo_CA1360** | CDS | |  | Hypothetical protein | 6.7 |
| **DGo_CA2084** | CDS | |  | Uracil-DNA glycosylase related enzyme | 6.5 |
| **DGo_PF0018** | CDS | |  | Hypothetical protein | 6.5 |
| **DGo_PB0479** | CDS | |  | Hypothetical protein | 6.4 |
| **DGo_CA2285** | CDS | |  | Glyoxalase/bleomycin resistance protein/dioxygenase | 6.4 |
| **DGo_PC0059** | CDS | |  | Hypothetical protein | 6.3 |
| **DGo_PC0222** | CDS | |  | Hypothetical protein | 6.3 |
| **DGo_PC0072** | CDS | |  | Hypothetical protein | 6.0 |
| **DGo_CA0381** | CDS | | *recG* | ATP-dependent DNA helicase RecG | 6.0 |
| **DGo_CA0897** | CDS | |  | Acetyltransferase, GNAT family protein | 5.9 |
| **DGo_CA1755** | CDS | |  | Allophanate hydrolase subunit 1 | 5.7 |
| **DGo_CA1378** | CDS | |  | Hypothetical protein | 5.6 |
| **DGo_CA1217** | CDS | |  | Hypothetical protein | 5.6 |
| **DGo_CA2720** | CDS | |  | Hypothetical protein | 5.6 |
| **DGo_CA2576** | CDS | | *rpsH* | 30S ribosomal protein S8 | 5.4 |
| **DGo_PC0028** | CDS | |  | Hypothetical protein | 5.4 |
| **DGo_PB0337** | CDS | |  | Transposase, putative | 5.3 |
| **DGo_CA1284** | CDS | |  | Hypothetical protein | 5.2 |
| **DGo_CA1240** | CDS | |  | Acylphosphatase | 5.1 |
| **DGo_PD0003** | CDS | |  | Hypothetical Membrane Spanning Protein | 5.1 |
| **DGo_CA0841** | CDS | |  | Hypothetical protein | 5.0 |
| **DGo_PC0063** | CDS | | *degP* | peptidase S1 and S6, chymotrypsin/Hap | 4.9 |
| **DGo_PB0279** | CDS | |  | Hypothetical protein | 4.9 |
| **DGo_PC0003** | CDS | |  | Transposase, IS4 | 4.9 |
| **DGo_CA2808** | CDS | | *lspA* | Lipoprotein signal peptidase | 4.9 |
| **DGo_CA1382** | CDS | |  | putative penicillin-binding protein 1B McrB, precursor | 4.9 |
| **DGo_PA0223** | CDS | |  | Permease of the drug/metabolite transporter superfamily | 4.8 |
| **DGo_CA1944** | CDS | |  | Hypothetical protein | 4.8 |
| **DGo_PA0159** | CDS | | *ssuC* | taurine transport system permease protein | 4.8 |
| **DGo_CA0154** | CDS | |  | ABC-type sugar transport system, permease component | 4.8 |
| **DGo_PB0504** | CDS | |  | Hypothetical protein | 4.7 |
| **DGo_PA0350** | CDS | |  | Permease of the drug/metabolite transporter superfamily | 4.7 |
| **DGo_CA2360** | CDS | |  | Hypothetical protein | 4.7 |
| **DGo_CA0585** | CDS | |  | alkaline phosphatase | 4.7 |
| **DGo_CA2869** | CDS | |  | Hypothetical protein | 4.6 |
| **DGo_CA1876** | CDS | |  | Hypothetical protein | 4.6 |
| **DGo_PC0269** | CDS | |  | Hypothetical protein | 4.6 |
| **DGo_PA0303** | CDS | | *frcA* | ABC transporter, nucleotide binding/ATPase protein | 4.5 |
| **DGo_PC0158** | CDS | |  | Hypothetical protein | 4.5 |
| **DGo_PC0238** | CDS | |  | Phage integrase family protein | 4.5 |
| **DGo_CA2529** | CDS | |  | Hypothetical protein | 4.4 |
| **DGo_PB0017** | CDS | |  | Hypothetical protein | 4.4 |
| **DGo_PC0031** | CDS | |  | Hypothetical protein | 4.4 |
| **DGo_CA0228** | CDS | |  | Serine/threonine protein kinase, putative | 4.1 |
| **DGo_CA0703** | CDS | |  | Hypothetical protein | 4.1 |
| **DGo_PC0183** | CDS | |  | Thioredoxin reductase | 4.1 |
| **DGo_PB0518** | CDS | |  | Hypothetical protein | 4.1 |
| **DGo_CA1154** | CDS | | *pstA* | ABC-type phosphate transport system, permease component | 4.1 |
| **DGo_CA0444** | CDS | |  | Phosphoesterase, PA-phosphatase related | 4.0 |
| **DGo_PB0288** | CDS | |  | Hypothetical protein | 4.0 |
| **DGo_PC0095** | CDS | |  | Hypothetical protein | 4.0 |
| **DGo_PA0133** | CDS | |  | Hypothetical protein | 4.0 |
| **DGo_PC0205** | CDS | |  | Transposase, IS4 | 4.0 |
| **DGo_PC0114** | CDS | |  | Hypothetical protein | 3.9 |
| **DGo_CA2802** | CDS | | *folK* | 2-amino-4-hydroxy-6-hydroxymethyldihydropteridine pyrophosphokinase | 3.9 |
| **DGo_CA0447** | CDS | | *proS* | Prolyl-tRNA synthetase, bacterial | 3.9 |
| **DGo_CA0583** | CDS | |  | ABC transporter, ATP-binding protein, putative | 3.8 |
| **DGo_PE0033** | CDS | |  | Hypothetical protein | 3.8 |
| **DGo_PB0495** | CDS | |  | Histidine kinase | 3.8 |
| **DGo_PC0115** | CDS | |  | Hypothetical protein | 3.8 |
| **DGo_CA0205** | CDS | | *cel* | M42 glutamyl aminopeptidase superfamily | 3.8 |
| **DGo_CA0687** | CDS | |  | Hypothetical protein | 3.7 |
| **DGo_CA0640** | CDS | | *gltB* | Glutamate synthase small subunit protein | 3.7 |
| **DGo_CA2419** | CDS | |  | Hypothetical protein | 3.7 |
| **DGo_PB0056** | CDS | |  | Hypothetical protein | 3.6 |
| **DGo_PC0036** | CDS | |  | Hypothetical protein | 3.6 |
| **DGo_PA0351** | CDS | |  | probable transcription regulator | 3.6 |
| **DGo_CA1233** | CDS | | *trkG* | K+ transporter Trk | 3.5 |
| **DGo_CA2390** | CDS | |  | Hypothetical protein | 3.5 |
| **DGo_CA1434** | CDS | |  | Hypothetical protein | 3.5 |
| **DGo_CA1040** | CDS | | *ddrI* | DdrI, transcriptional regulator, Crp/Fnr family | 3.5 |
| **DGo_CA0616** | CDS | |  | ABC transporter, permease protein, putative | 3.5 |
| **DGo_CA1441** | CDS | |  | Hypothetical protein | 3.5 |
| **DGo_PB0018** | CDS | |  | Hypothetical protein | 3.5 |
| **DGo_PC0274** | CDS | |  | Hypothetical protein | 3.5 |
| **DGo_PA0251** | CDS | | *bceK* | Glycosyl transferase group 1 | 3.5 |
| **DGo_PB0329** | CDS | | *potC* | ABC transporter, membrane spanning protein | 3.5 |
| **DGo_CA1548** | CDS | |  | Hypothetical protein | 3.5 |
| **DGo_CA0217** | CDS | |  | Hypothetical protein | 3.5 |
| **DGo_CA1652** | CDS | |  | Hypothetical protein | 3.4 |
| **DGo_CA0551** | CDS | | *iolE* | Xylose isomerase domain protein TIM barrel | 3.4 |
| **DGo_PB0522** | CDS | |  | Hypothetical protein | 3.4 |
| **DGo_PB0237** | CDS | |  | Hypothetical protein | 3.4 |
| **DGo_PC0138** | CDS | |  | Hypothetical protein | 3.4 |
| **DGo_CA0552** | CDS | | *ccpA* | Catabolite control protein A, glucose-resistance amylase regulator | 3.4 |
| **DGo_CA0952** | CDS | |  | Hypothetical protein | 3.4 |
| **DGo_CA1627** | CDS | |  | ATP phosphoribosyltransferase | 3.4 |
| **DGo_PB0002** | CDS | | *trpR* | LysR family transcriptional regulator | 3.4 |
| **DGo_PC0279** | CDS | |  | Hypothetical protein | 3.3 |
| **DGo_CA1021** | CDS | | *ytxJ* | Thioredoxin family protein, general stress protein ytxJ-like protein | 3.3 |
| **DGo_CA0308** | CDS | | *folC2* | FolC bifunctional protein | 3.3 |
| **DGo_PB0338** | CDS | |  | Hypothetical protein | 3.3 |
| **DGo_CA1056** | CDS | |  | Beta-lactamase | 3.3 |
| **DGo_CA1482** | CDS | |  | Hypothetical protein | 3.3 |
| **DGo_CA2179** | CDS | |  | Ankyrin domain protein | 3.3 |
| **DGo_CA0137** | CDS | |  | Endoribonuclease L-PSP | 3.3 |
| **DGo_PB0220** | CDS | |  | Peptidase C39 | 3.3 |
| **DGo_PB0175** | CDS | |  | Hypothetical protein | 3.3 |
| **DGo_PA0349** | CDS | |  | Hypothetical protein | 3.3 |
| **DGo_CA2402** | CDS | |  | ABC glutamate/glutamine/aspartate/asparagine transporter, ATPase subunit bztD | 3.3 |
| **DGo_PB0164** | CDS | |  | Hypothetical protein | 3.2 |
| **DGo_CA1552** | CDS | |  | Hypothetical protein | 3.2 |
| **DGo_PB0177** | CDS | | *pilT* | Tfp pilus assembly protein, pilus retraction ATPase PilT | 3.2 |
| **DGo_CA2018** | CDS | | *nrfF* | Formate-dependent nitrite reductase complex, nrfF subunit | 3.2 |
| **DGo_CA1377** | CDS | |  | IG Hypothetical 18565 | 3.2 |
| **DGo_PD0021** | CDS | |  | Hypothetical protein | 3.2 |
| **DGo_PA0053** | CDS | |  | Probable transcriptional regulator, MarR family protein | 3.2 |
| **DGo_PB0384** | CDS | |  | Hypothetical protein | 3.2 |
| **DGo_CA0607** | CDS | | *phrB* | Deoxyribodipyrimidine photo-lyase type II | 3.2 |
| **DGo_CA1981** | CDS | |  | Hypothetical protein | 3.2 |
| **DGo_CA2718** | CDS | |  | Hypothetical protein | 3.2 |
| **DGo_PF0026** | CDS | |  | Hypothetical protein | 3.1 |
| **DGo_CA0695** | CDS | |  | Glycoside hydrolase, family 43 | 3.1 |
| **DGo_CA0073** | CDS | | *arsR* | Transcriptional regulator, ArsR family | 3.1 |
| **DGo_CA1832** | CDS | |  | Sugar transporter, putative | 3.1 |
| **DGo_CA2609** | CDS | |  | Trypsin-like peptidase and PDZ domain | 3.1 |
| **DGo_PC0237** | CDS | |  | Phage integrase family protein | 3.1 |
| **DGo_PD0002** | CDS | |  | Hypothetical protein | 3.1 |
| **DGo_CA0690** | CDS | |  | Hypothetical protein | 3.1 |
| **DGo_CA0548** | CDS | | *moeZ* | UBA/THIF-type NAD/FAD binding fold | 3.1 |
| **DGo_CA2789** | CDS | |  | Hypothetical protein | 3.1 |
| **DGo_CA2118** | CDS | |  | Hypothetical protein | 3.1 |
| **DGo_CA0039** | CDS | |  | Hypothetical protein | 3.0 |
| **DGo_PB0420** | CDS | |  | Hypothetical protein | 3.0 |
| **DGo_CA2694** | CDS | |  | Hypothetical protein | 3.0 |
| **DGo_PC0273** | CDS | |  | Hypothetical protein | 3.0 |
| **DGo_PC0098** | CDS | | *recB2* | Predicted nuclease, RecB family | 3.0 |
| **DGo_CA1872** | CDS | | *tauA2* | ABC transporter, periplasmic substrate-binding protein, putative | 3.0 |
| **DGo_PB0284** | CDS | |  | Hypothetical protein | 2.9 |
| **DGo_CA1849** | CDS | |  | Hypothetical protein | 2.9 |
| **DGo_PB0397** | CDS | |  | Hypothetical protein | 2.9 |
| **DGo_CA1019** | CDS | |  | Hypothetical protein | 2.9 |
| **DGo_PB0412** | CDS | |  | Hypothetical protein | 2.9 |
| **DGo_PB0421** | CDS | |  | Acetyltransferase, GNAT family | 2.9 |
| **DGo_CA1531** | CDS | |  | Hypothetical protein | 2.9 |
| **DGo_CA2839** | CDS | |  | Hypothetical protein | 2.9 |
| **DGo_PA0046** | CDS | |  | Putative phosphatase protein | 2.9 |
| **DGo_CA2057** | CDS | |  | DinB/YfiT family metal-binding protein | 2.9 |
| **DGo_PB0355** | CDS | |  | Hypothetical protein | 2.9 |
| **DGo_PB0139** | CDS | |  | Hypothetical protein | 2.9 |
| **DGo_CA0090** | CDS | |  | Mg2+ transporter protein, CorA-like protein | 2.9 |
| **DGo_PC0143** | CDS | |  | Transcriptional regulator with ATPase activity | 2.8 |
| **DGo_CA1111** | CDS | | *pepB* | Leucyl aminopeptidase, putative | 2.8 |
| **DGo_PC0120** | CDS | |  | Hypothetical protein | 2.8 |
| **DGo_CA2860** | CDS | | *purC* | Phosphoribosylaminoimidazole-succinocarboxamide synthase | 2.8 |
| **DGo_CA1540** | CDS | |  | Hypothetical protein | 2.8 |
| **DGo_CA1318** | CDS | |  | Short-chain dehydrogenase/reductase SDR | 2.8 |
| **DGo_CA0791** | CDS | |  | dTDP-4-dehydrorhamnose reductase and Glycosyl hydrolase family 1 | 2.8 |
| **DGo_CA1653** | CDS | |  | Hypothetical protein | 2.8 |
| **DGo_PF0003** | CDS | |  | Hypothetical protein | 2.8 |
| **DGo_PA0116** | CDS | |  | Integrase/recombinase, putative | 2.8 |
| **DGo_PB0247** | CDS | |  | Hypothetical protein | 2.8 |
| **DGo_CA2761** | CDS | | *folC* | Folylpolyglutamate synthetase | 2.8 |
| **DGo_CA0555** | CDS | |  | Yellow-related protein | 2.8 |
| **DGo_CA1850** | CDS | |  | Hypothetical protein | 2.8 |
| **DGo_PB0507** | CDS | |  | Hypothetical protein | 2.8 |
| **DGo_CA0795** | CDS | |  | Hypothetical protein | 2.8 |
| **DGo_CA1589** | CDS | |  | Dipeptidase M19 | 2.7 |
| **DGo_CA1804** | CDS | |  | Transcriptional regulator, TetR family | 2.7 |
| **DGo_PC0175** | CDS | | *phoR* | Sensor protein | 2.7 |
| **DGo_PC0191** | CDS | |  | Hypothetical protein | 2.7 |
| **DGo_PA0125** | CDS | |  | FRG domain protein | 2.7 |
| **DGo_PB0395** | CDS | |  | Hypothetical protein | 2.7 |
| **DGo_CA0277** | CDS | |  | Hypothetical protein | 2.7 |
| **DGo_CA1165** | CDS | | *pyrD* | dihydroorotate dehydrogenase 2 | 2.7 |
| **DGo_CA2944** | CDS | |  | Hypothetical protein | 2.7 |
| **DGo_PB0330** | CDS | |  | Hypothetical protein | 2.7 |
| **DGo_CA2242** | CDS | | *ftsW* | Cell cycle protein | 2.7 |
| **DGo_CA1782** | CDS | |  | HAD superfamily hydrolase | 2.7 |
| **DGo_CA2366** | CDS | |  | Hypothetical protein | 2.7 |
| **DGo_PB0515** | CDS | |  | Hypothetical protein | 2.7 |
| **DGo_CA0712** | CDS | |  | Hypothetical protein | 2.7 |
| **DGo_PB0264** | CDS | |  | Hypothetical protein | 2.7 |
| **DGo_CA0376** | CDS | | *yqgF* | Holliday junction resolvase YqgF | 2.7 |
| **DGo_CA1759** | CDS | |  | Hypothetical protein | 2.7 |
| **DGo_CA2391** | CDS | |  | Peptidase S58, DmpA | 2.7 |
| **DGo_PB0170** | CDS | |  | Hypothetical protein | 2.7 |
| **DGo_PB0292** | CDS | |  | Hypothetical protein | 2.7 |
| **DGo_PB0148** | CDS | |  | Hypothetical protein | 2.6 |
| **DGo_CA2425** | CDS | |  | Nitrilase/cyanide hydratase and apolipoprotein N-acyltransferase | 2.6 |
| **DGo_PB0437** | CDS | |  | Hypothetical protein | 2.6 |
| **DGo_CA0682** | CDS | |  | Hypothetical protein | 2.6 |
| **DGo_CA0005** | CDS | |  | Mg2+ transporter protein, CorA-like protein | 2.6 |
| **DGo_PB0106** | CDS | |  | Diguanylate cyclase/phosphodiesterase with PAS/PAC and GAF sensor | 2.6 |
| **DGo_PC0215** | CDS | |  | Hypothetical protein | 2.6 |
| **DGo_CA1142** | CDS | | *secE* | Preprotein translocase, SecE subunit | 2.6 |
| **DGo_CA2771** | CDS | |  | Hypothetical protein | 2.6 |
| **DGo_PF0014** | CDS | |  | Hypothetical protein | 2.6 |
| **DGo_CA1005** | CDS | | *pfkB* | Fructokinase, ScrK | 2.6 |
| **DGo_CA2438** | CDS | |  | Hypothetical protein | 2.6 |
| **DGo_CA0441** | CDS | |  | Hypothetical protein | 2.6 |
| **DGo_CA2736** | CDS | |  | Hypothetical transmembrane protein | 2.6 |
| **DGo_CA0049** | CDS | |  | Hypothetical protein | 2.6 |
| **DGo_CA2543** | CDS | |  | ADP-ribosylglycohydrolase, putative | 2.6 |
| **DGo_CA0996** | CDS | | *potA* | Spermidine/putrescine ABC transporter, ATP-binding protein | 2.6 |
| **DGo_PB0353** | CDS | |  | Hypothetical protein | 2.6 |
| **DGo_PC0044** | CDS | |  | Hypothetical protein | 2.6 |
| **DGo_PA0091** | CDS | |  | Menaquinol-cytochrome c reductase iron-sulfur subunit | 2.5 |
| **DGo_CA1745** | CDS | |  | putative phosphatase | 2.5 |
| **DGo_CA1659** | CDS | |  | Glycosyl transferase, family 4 | 2.5 |
| **DGo_PB0215** | CDS | |  | Hypothetical protein | 2.5 |
| **DGo_PD0004** | CDS | |  | SMI1 / KNR4 family | 2.5 |
| **DGo_PC0226** | CDS | |  | Protein tyrosine phosphatase | 2.5 |
| **DGo_CA2050** | CDS | |  | Hypothetical protein | 2.5 |
| **DGo_CA0019** | CDS | |  | transcriptional regulator | 2.5 |
| **DGo_PB0198** | CDS | |  | Hypothetical protein | 2.5 |
| **DGo_PF0010** | CDS | |  | putative transposase | 2.5 |
| **DGo_PC0181** | CDS | |  | Hypothetical protein | 2.5 |
| **DGo_CA1591** | CDS | | *ykfB* | Mandelate racemase-like protein/ muconate lactonizing enzyme-like protein | 2.5 |
| **DGo_PB0387** | CDS | |  | Pentapeptide repeat protein | 2.5 |
| **DGo_PB0032** | CDS | |  | Hypothetical protein | 2.5 |
| **DGo_CA2038** | CDS | |  | Hypothetical protein | 2.5 |
| **DGo_CA1955** | CDS | |  | Hypothetical protein | 2.5 |
| **DGo_CA1178** | CDS | |  | Hypothetical protein | 2.5 |
| **DGo_CA1542** | CDS | |  | Hypothetical protein | 2.5 |
| **DGo_CA1957** | CDS | |  | Hypothetical protein | 2.5 |
| **DGo_PB0519** | CDS | |  | DEAD/DEAH box helicase domain protein | 2.5 |
| **DGo_CA0794** | CDS | |  | YceI-like protein, ycel3 | 2.4 |
| **DGo_PA0246** | CDS | |  | Hypothetical protein | 2.4 |
| **DGo_PB0072** | CDS | |  | Hypothetical protein | 2.4 |
| **DGo_CA2290** | CDS | | *gidA* | GidA-related protein | 2.4 |
| **DGo_CA0943** | CDS | | *relA* | RelA/SpoT domain protein | 2.4 |
| **DGo_CA2306** | CDS | |  | Probable transcriptional regulator | 2.4 |
| **DGo_PB0189** | CDS | |  | Hypothetical protein | 2.4 |
| **DGo_CA2558** | CDS | |  | Hypothetical protein | 2.4 |
| **DGo_CA0235** | CDS | |  | Hypothetical protein | 2.4 |
| **DGo_CA0186** | CDS | |  | Hypothetical protein | 2.4 |
| **DGo_PB0095** | CDS | |  | DNA polymerase | 2.4 |
| **DGo_PA0373** | CDS | |  | Hypothetical protein | 2.4 |
| **DGo_CA0367** | CDS | | *ade* | Adenine deaminase | 2.4 |
| **DGo_CA2479** | CDS | |  | 50S ribosomal protein L25 | 2.4 |
| **DGo_PC0061** | CDS | |  | Hypothetical protein | 2.4 |
| **DGo_PD0033** | CDS | |  | DNA mismatch endonuclease vsr | 2.4 |
| **DGo_PA0139** | CDS | |  | WD40-like repeat/amidohydrolase domain protein | 2.4 |
| **DGo_CA0017** | CDS | |  | Hypothetical protein | 2.4 |
| **DGo_CA2857** | CDS | |  | Low molecular weight protein-tyrosine-phosphatase | 2.4 |
| **DGo_PF0025** | CDS | |  | Phage integrase | 2.4 |
| **DGo_CA1533** | CDS | |  | Putative cation efflux family protein, CzcD-like protein | 2.4 |
| **DGo_PB0138** | CDS | |  | Hypothetical protein | 2.4 |
| **DGo_CA2821** | CDS | |  | Beta-lactamase domain protein | 2.4 |
| **DGo_CA2079** | CDS | |  | GCN5-related N-acetyltransferase | 2.4 |
| **DGo_CA1628** | CDS | |  | Intradiol ring-cleavage dioxygenase | 2.4 |
| **DGo_CA0002** | CDS | | *dnaN* | DNA polymerase III, beta subunit | 2.4 |
| **DGo_CA2172** | CDS | |  | Hypothetical protein | 2.3 |
| **DGo_CA1536** | CDS | |  | Zinc finger, DHP-type | 2.3 |
| **DGo_PC0030** | CDS | |  | Integrase/recombinase, putative | 2.3 |
| **DGo_CA0820** | CDS | |  | Hypothetical protein | 2.3 |
| **DGo_PA0185** | CDS | |  | Hypothetical protein | 2.3 |
| **DGo_PD0031** | CDS | |  | Transposase, IS4 | 2.3 |
| **DGo_PB0367** | CDS | |  | Transcriptional regulator-like protein | 2.3 |
| **DGo_CA2114** | CDS | |  | Hypothetical protein | 2.3 |
| **DGo_CA1844** | CDS | |  | Hypothetical protein | 2.3 |
| **DGo_PD0023** | CDS | |  | Sulfotransferase domain superfamily | 2.3 |
| **DGo_PA0136** | CDS | |  | DUF790-containing protein | 2.3 |
| **DGo_CA1334** | CDS | | *argJ* | Arginine biosynthesis bifunctional protein argJ beta chain | 2.3 |
| **DGo_CA0534** | CDS | |  | 3-deoxy-7-phosphoheptulonate synthase | 2.3 |
| **DGo_CA1181** | CDS | |  | Predicted RNA-binding protein associated with RNAse G/E | 2.3 |
| **DGo_PC0121** | CDS | |  | Hypothetical protein | 2.3 |
| **DGo_CA1335** | CDS | |  | HTH transcriptional regulator | 2.3 |
| **DGo_CA0420** | CDS | |  | ABC-type polyamine transport system, ATPase component | 2.3 |
| **DGo_CA1725** | CDS | | *miaA* | isopentenylpyrophosphate transferase | 2.3 |
| **DGo_PA0333** | CDS | |  | Probable macrolide-efflux transmembrane protein | 2.3 |
| **DGo_PB0347** | CDS | |  | Hypothetical protein | 2.3 |
| **DGo_CA2726** | CDS | |  | Hypothetical protein | 2.3 |
| **DGo_PA0192** | CDS | |  | Branched-chain amino acid ABC-type transport system, ATPase component | 2.2 |
| **DGo_PC0102** | CDS | |  | Hypothetical protein | 2.2 |
| **DGo_CA2032** | CDS | |  | ABC transporter, ATP-binding protein | 2.2 |
| **DGo_PE0004** | CDS | |  | Hypothetical protein | 2.2 |
| **DGo_CA0263** | CDS | |  | Hypothetical protein | 2.2 |
| **DGo_CA0550** | CDS | | *carD* | Transcriptional regulator, CarD family | 2.2 |
| **DGo_CA2738** | CDS | | *fliY* | ABC-type amino acid transport/signal transduction system, periplasmic component | 2.2 |
| **DGo_PB0433** | CDS | | *parB* | ParB-like nuclease domain family | 2.2 |
| **DGo_PE0065** | CDS | |  | Hypothetical protein | 2.2 |
| **DGo_CA1678** | CDS | |  | Alcohol dehydrogenase, zinc-binding | 2.2 |
| **DGo_PB0228** | CDS | |  | Bifunctional DNA primase/polymerase | 2.2 |
| **DGo_CA2819** | CDS | |  | Hypothetical protein | 2.2 |
| **DGo_PA0162** | CDS | |  | Putative Permease of the major facilitator superfamily | 2.2 |
| **DGo_PC0236** | CDS | |  | Phage integrase family protein | 2.2 |
| **DGo_PA0309** | CDS | | *lamB* | LamB/YcsF family protein | 2.2 |
| **DGo_CA2007** | CDS | |  | Hypothetical protein | 2.2 |
| **DGo_CA0928** | CDS | |  | Hypothetical protein | 2.2 |
| **DGo_PB0212** | CDS | |  | Hypothetical protein | 2.2 |
| **DGo_CA1866** | CDS | |  | Transcriptional regulator, TetR family | 2.2 |
| **DGo_CA0237** | CDS | |  | Pilin, type IV, putative | 2.2 |
| **DGo_PA0115** | CDS | |  | Hypothetical protein | 2.2 |
| **DGo_CA0426** | CDS | |  | Epoxide Hydrolase | 2.2 |
| **DGo_CA0350** | CDS | | *ddrB* | DdrB, radiation induced single-stranded DNA binding protein | 2.2 |
| **DGo_CA2354** | CDS | | *tatC* | Sec-independent protein translocase protein TatC, putative | 2.2 |
| **DGo_PA0338** | CDS | | *metI* | ABC transporter, permease protein | 2.2 |
| **DGo_PB0045** | CDS | |  | Hypothetical protein | 2.2 |
| **DGo_CA1917** | CDS | |  | TetR-family transcriptional regulator | 2.2 |
| **DGo_PC0073** | CDS | |  | Hypothetical protein | 2.2 |
| **DGo_CA1241** | CDS | | *osmC* | OsmC-like protein | 2.1 |
| **DGo_CA0647** | CDS | | *kdpC* | Potassium-transporting ATPase C chain | 2.1 |
| **DGo_CA1027** | CDS | |  | Uncharacterized peroxidase-related | 2.1 |
| **DGo_CA2029** | CDS | |  | Hypothetical protein | 2.1 |
| **DGo_CA0071** | CDS | | *ddrC* | DdrC | 2.1 |
| **DGo_CA0977** | CDS | | *str* | Streptomycin 3''-kinase | 2.1 |
| **DGo_PB0267** | CDS | |  | Hypothetical protein | 2.1 |
| **DGo_CA0636** | CDS | |  | ABC transporter, permease protein | 2.1 |
| **DGo_PC0019** | CDS | |  | Hypothetical protein | 2.1 |
| **DGo_CA0920** | CDS | |  | SCP/PR1 domain | 2.1 |
| **DGo_CA1579** | CDS | |  | Hydrolase, alpha/beta hydrolase fold family | 2.1 |
| **DGo_CA2773** | CDS | |  | Hypothetical protein | 2.1 |
| **DGo_CA0956** | CDS | |  | Hypothetical protein | 2.1 |
| **DGo_PB0205** | CDS | |  | Hypothetical protein | 2.1 |
| **DGo_CA0688** | CDS | |  | Hypothetical protein | 2.1 |
| **DGo_PC0240** | CDS | |  | Transcriptional regulator, LysR family | 2.1 |
| **DGo_PA0379** | CDS | | *manA* | Mannose-6-phosphate isomerase, putative | 2.1 |
| **DGo_CA1873** | CDS | |  | chelatase family protein | 2.1 |
| **DGo_CA2180** | CDS | |  | Luciferase-like protein | 2.1 |
| **DGo_PB0249** | CDS | |  | AAA ATPase | 2.1 |
| **DGo_CA1763** | CDS | | *yhfA* | OsmC-like protein | 2.1 |
| **DGo_CA1726** | CDS | | *hsp* | Heat shock protein Hsp20 | 2.1 |
| **DGo_CA2102** | CDS | |  | Hypothetical protein | 2.1 |
| **DGo_CA0131** | CDS | |  | Phenylacetic acid degradation protein PaaD | 2.1 |
| **DGo_PE0006** | CDS | |  | Hypothetical protein | 2.1 |
| **DGo_PC0011** | CDS | | *tniB* | Transposon, transposition helper protein C, putative | 2.1 |
| **DGo_CA2193** | CDS | | *alr* | Alanine racemase | 2.1 |
| **DGo_CA2666** | CDS | |  | Hypothetical protein | 2.1 |
| **DGo_CA2614** | CDS | |  | Peptidase M20 | 2.1 |
| **DGo_CA0719** | CDS | | *deoR* | DeoR-family transcriptional regulator | 2.1 |
| **DGo_CA0653** | CDS | |  | Transcriptional regulator, IclR family | 2.1 |
| **DGo_CA0312** | CDS | | *moaC* | Molybdenum cofactor biosynthesis protein C | 2.1 |
| **DGo_PA0014** | CDS | |  | Hypothetical protein | 2.1 |
| **DGo_CA0335** | CDS | | *ctaB* | Protoheme IX farnesyltransferase | 2.1 |
| **DGo_PB0182** | CDS | |  | Hypothetical protein | 2.0 |
| **DGo_PB0081** | CDS | |  | Hypothetical protein | 2.0 |
| **DGo_CA0873** | CDS | | *gyrB* | putative DNA topoisomerase | 2.0 |
| **DGo_CA0633** | CDS | | *argE* | Amidase, hydantoinase/carbamoylase family | 2.0 |
| **DGo_CA2308** | CDS | |  | Hypothetical protein | 2.0 |
| **DGo_CA2014** | CDS | |  | Hypothetical protein | 2.0 |
| **DGo_CA0725** | CDS | | *sig4* | RNA polymerase sigma factor | 2.0 |
| **DGo_CA0843** | CDS | |  | HAD-superfamily hydrolase subfamily IA | 2.0 |
| **DGo_PB0239** | CDS | |  | Hypothetical protein | 2.0 |
| **DGo_PB0413** | CDS | |  | Phage protein | 2.0 |
| **DGo_CA0332** | CDS | | *panB* | 3-methyl-2-oxobutanoate hydroxymethyltransferase | 2.0 |
| **DGo_PE0066** | CDS | |  | Hypothetical protein | 2.0 |
| **DGo_CA2368** | CDS | |  | Lipase, putative | 2.0 |
| **DGo_CA0357** | CDS | | *citB* | response regulator receiver/unknown domain-containing protein | 2.0 |
| **DGo_CA2138** | CDS | |  | Aminotransferase, class V | 2.0 |
| **DGo_PC0032** | CDS | |  | Hypothetical protein | 2.0 |
| **DGo_PB0396** | CDS | |  | Hypothetical protein | 2.0 |
| **DGo_CA1259** | CDS | |  | GGDEF family protein | 2.0 |
| **B. Functional description of 754 genes that were repressed after UV-irradiation** | | | | | |
| **Locus_tag** | | **Type** | **Name** | **Description** | **Fold** |
| **DGo_CAnc10** | | ncRNA |  | Hypothetical non-coding RNA | <-100.0 |
| **DGo_PBnc01** | | ncRNA |  | Hypothetical non-coding RNA | <-100.0 |
| **DGo_CAnc16** | | ncRNA |  | Hypothetical non-coding RNA | -109.5 |
| **DGo_PFnc04** | | ncRNA |  | Hypothetical non-coding RNA | -45.9 |
| **DGo_PBnc02** | | ncRNA |  | Hypothetical non-coding RNA | -32.6 |
| **DGo_PAnc04** | | ncRNA |  | Hypothetical non-coding RNA | -26.5 |
| **DGo_PBnc04** | | ncRNA |  | Hypothetical non-coding RNA | -23.3 |
| **DGo_PCnc05** | | ncRNA |  | Hypothetical non-coding RNA | -20.3 |
| **DGo_CAnc06** | | ncRNA |  | Hypothetical non-coding RNA | -19.3 |
| **DGo_PAnc02** | | ncRNA |  | Hypothetical non-coding RNA | -17.9 |
| **DGo_PAnc03** | | ncRNA |  | Hypothetical non-coding RNA | -17.6 |
| **DGo_PFnc01** | | ncRNA |  | Hypothetical non-coding RNA | -14.3 |
| **DGo_PCnc01** | | ncRNA |  | Hypothetical non-coding RNA | -6.4 |
| **DGo_PFnc03** | | ncRNA |  | Hypothetical non-coding RNA | -6.0 |
| **DGo_CAnc02** | | ncRNA |  | Hypothetical non-coding RNA | -5.5 |
| **DGo_CAnc01** | | ncRNA |  | Hypothetical non-coding RNA | -5.5 |
| **DGo_PCnc03** | | ncRNA |  | Hypothetical non-coding RNA | -3.5 |
| **DGo_PCnc02** | | ncRNA |  | Hypothetical non-coding RNA | -3.5 |
| **DGo_PCnc04** | | ncRNA |  | Hypothetical non-coding RNA | -2.9 |
| **DGo_PBnc06** | | ncRNA |  | Hypothetical non-coding RNA | -2.9 |
| **DGo_CAnc07** | | ncRNA |  | Hypothetical non-coding RNA | -2.7 |
| **DGo_PFnc02** | | ncRNA |  | Hypothetical non-coding RNA | -2.2 |
| **DGo_CA1065** | | CDS |  | Hypothetical protein | <-100.0 |
| **DGo_CA1511** | | CDS |  | Yfit/DinB family protein | <-100.0 |
| **DGo_CA2661** | | CDS | *nth* | putative endonuclease III | <-100.0 |
| **DGo_PB0060** | | CDS |  | Hypothetical protein | <-100.0 |
| **DGo_PB0425** | | CDS |  | Hypothetical protein | <-100.0 |
| **DGo_PC0090** | | CDS |  | Hypothetical protein | <-100.0 |
| **DGo_PC0149** | | CDS |  | Hypothetical protein | <-100.0 |
| **DGo_PC0178** | | CDS |  | Hypothetical protein | <-100.0 |
| **DGo_CA0923** | | CDS |  | Hypothetical protein | -124.8 |
| **DGo_PD0013** | | CDS |  | Hypothetical protein | -53.4 |
| **DGo_PB0054** | | CDS | *arsR* | Transcriptional regulator, ArsR family | -51.3 |
| **DGo_CA1925** | | CDS |  | Signal peptidase I | -45.1 |
| **DGo_PB0370** | | CDS |  | Transposase, ISMyma01_aa2-like protein | -32.5 |
| **DGo_CA1593** | | CDS | *degV* | DegV family protein | -19.2 |
| **DGo_PB0028** | | CDS |  | Hypothetical protein | -17.3 |
| **DGo_PB0254** | | CDS |  | Hypothetical protein | -16.8 |
| **DGo_PB0082** | | CDS |  | Hypothetical protein | -16.4 |
| **DGo_PB0456** | | CDS |  | Hypothetical protein | -15.2 |
| **DGo_CA1964** | | CDS |  | Hypothetical protein | -14.5 |
| **DGo_PC0124** | | CDS |  | Hypothetical protein | -14.2 |
| **DGo_CA1357** | | CDS |  | Hypothetical protein | -13.7 |
| **DGo_PB0265** | | CDS |  | Hypothetical protein | -13.6 |
| **DGo_CA0268** | | CDS |  | ABC-type branched-chain amino acid transport system, ATPase component | -12.9 |
| **DGo_PB0253** | | CDS |  | Hypothetical protein | -12.2 |
| **DGo_PB0262** | | CDS |  | Hypothetical protein | -12.1 |
| **DGo_PC0268** | | CDS |  | Hypothetical protein | -12.0 |
| **DGo_PB0414** | | CDS |  | Hypothetical protein | -11.8 |
| **DGo_CA1778** | | CDS |  | Transcriptional regulator, MarR family | -11.4 |
| **DGo_CA1899** | | CDS |  | Hypothetical protein | -11.0 |
| **DGo_CA2803** | | CDS | *folB* | Dihydroneopterin aldolase | -10.8 |
| **DGo_PB0273** | | CDS |  | Hypothetical protein | -10.7 |
| **DGo_CA1149** | | CDS | *rbsC* | Ribose/xylose/arabinose/galactoside ABC-type transport systems, permease components | -10.1 |
| **DGo_CA2449** | | CDS |  | Glyoxalase/bleomycin resistance protein/dioxygenase | -8.9 |
| **DGo_PC0079** | | CDS |  | Helicase-like protein | -8.6 |
| **DGo_PB0475** | | CDS |  | Hypothetical protein | -8.5 |
| **DGo_PC0045** | | CDS |  | Hypothetical protein | -8.5 |
| **DGo_CA0173** | | CDS |  | Hypothetical protein | -8.3 |
| **DGo_PC0232** | | CDS |  | Hypothetical protein | -8.2 |
| **DGo_PB0158** | | CDS | *hofG* | Tfp pilus assembly protein major pilin PilA-like protein | -8.0 |
| **DGo_CA1545** | | CDS | *ccmA* | ABC-type transport system | -7.8 |
| **DGo_CA0419** | | CDS |  | Acetyltransferase, putative | -7.7 |
| **DGo_CA1379** | | CDS |  | Putative acetylglutamate kinase-like protein | -7.7 |
| **DGo_CA1900** | | CDS |  | Hypothetical protein | -7.7 |
| **DGo_PC0235** | | CDS |  | Hypothetical protein | -7.6 |
| **DGo_PB0073** | | CDS |  | Transposase IS4 family protein | -7.5 |
| **DGo_PB0362** | | CDS |  | Hypothetical protein | -7.5 |
| **DGo_CA2022** | | CDS |  | Hypothetical protein | -7.4 |
| **DGo_CA2719** | | CDS |  | putative acyltransferase | -7.4 |
| **DGo_CA2060** | | CDS |  | Sec-C motif containing protein | -7.0 |
| **DGo_CA1244** | | CDS |  | Transcriptional regulator, AraC family | -7.0 |
| **DGo_CA2358** | | CDS | *crtI3* | Phytoene dehydrogenase, CrtI | -7.0 |
| **DGo_CA1906** | | CDS |  | Hypothetical protein | -7.0 |
| **DGo_PA0347** | | CDS |  | Hypothetical protein | -6.9 |
| **DGo_PB0453** | | CDS |  | Hypothetical protein | -6.9 |
| **DGo_CA1702** | | CDS |  | Predicted RNA-binding protein | -6.9 |
| **DGo_CA1570** | | CDS |  | YceI like family protein | -6.8 |
| **DGo_CA0906** | | CDS |  | Endoribonuclease L-PSP superfamily | -6.8 |
| **DGo_PA0359** | | CDS | *rbsB2* | Ribose ABC transporter, periplasmic ribose-binding protein | -6.8 |
| **DGo_PC0018** | | CDS |  | Hypothetical protein | -6.8 |
| **DGo_CA0105** | | CDS |  | Hypothetical protein | -6.8 |
| **DGo_PB0092** | | CDS |  | Transposase IS4 family protein | -6.8 |
| **DGo_CA1602** | | CDS | *argC* | N-acetyl-gamma-glutamyl-phosphate reductase | -6.7 |
| **DGo_PB0033** | | CDS |  | Hypothetical protein | -6.7 |
| **DGo_PB0084** | | CDS |  | Oxidoreductase domain protein | -6.7 |
| **DGo_PC0184** | | CDS |  | Hypothetical protein | -6.7 |
| **DGo_PB0351** | | CDS |  | Hypothetical protein | -6.6 |
| **DGo_PB0207** | | CDS |  | Transposase, IS4 | -6.5 |
| **DGo_CA0772** | | CDS |  | Peptide ABC transporter, permease protein | -6.4 |
| **DGo_PB0099** | | CDS |  | Hypothetical protein | -6.2 |
| **DGo_PC0099** | | CDS |  | Hypothetical protein | -6.2 |
| **DGo_CA1965** | | CDS |  | Terminase small subunit | -6.2 |
| **DGo_CA1278** | | CDS | *clpS* | ATP-dependent Clp protease adaptor protein ClpS | -6.2 |
| **DGo_CA1890** | | CDS |  | Putative gene transfer agent portal protein | -6.1 |
| **DGo_CA1688** | | CDS |  | Hypothetical protein | -6.1 |
| **DGo_PB0484** | | CDS |  | Hypothetical protein | -6.1 |
| **DGo_PB0048** | | CDS |  | GCN5-related N-acetyltransferase | -6.1 |
| **DGo_CA1239** | | CDS |  | putative octaprenyl-diphosphate synthase | -6.0 |
| **DGo_PC0066** | | CDS |  | Hypothetical protein | -6.0 |
| **DGo_CA1305** | | CDS |  | Response regulator | -5.9 |
| **DGo_CA1299** | | CDS |  | Maltose ABC transporter, periplasmic maltose-binding protein | -5.9 |
| **DGo_PA0106** | | CDS |  | Hypothetical protein | -5.9 |
| **DGo_CA1654** | | CDS |  | Hypothetical protein | -5.8 |
| **DGo_CA1989** | | CDS |  | Hypothetical protein | -5.8 |
| **DGo_PA0178** | | CDS |  | Hypothetical protein | -5.8 |
| **DGo_CA0745** | | CDS |  | Hypothetical protein | -5.7 |
| **DGo_CA2031** | | CDS | *csaA* | T-RNA-binding region | -5.6 |
| **DGo_CA0098** | | CDS |  | Hypothetical protein | -5.6 |
| **DGo_PF0009** | | CDS |  | Hypothetical protein | -5.6 |
| **DGo_CA1476** | | CDS | *fabZ* | dehydratase | -5.5 |
| **DGo_CA1294** | | CDS |  | MutT/nudix family protein | -5.5 |
| **DGo_CA2199** | | CDS |  | Hypothetical protein | -5.5 |
| **DGo_PB0376** | | CDS |  | Transposase, putative | -5.5 |
| **DGo_PC0146** | | CDS |  | Hypothetical protein | -5.4 |
| **DGo_PB0071** | | CDS |  | Hypothetical protein | -5.4 |
| **DGo_CA2389** | | CDS |  | Uroporphyrin-III methyltransferase and synthase domains | -5.4 |
| **DGo_CA2036** | | CDS | *alkA* | Putative 3-methyladenine DNA glycosylase | -5.4 |
| **DGo_CA0446** | | CDS |  | Hypothetical protein | -5.4 |
| **DGo_CA1625** | | CDS |  | Hypothetical protein | -5.4 |
| **DGo_PA0105** | | CDS |  | Probable RNA 2'-phosphotransferase | -5.3 |
| **DGo_PC0164** | | CDS |  | Hypothetical protein | -5.3 |
| **DGo_CA2907** | | CDS | *xseB* | Exonuclease VII small subunit | -5.3 |
| **DGo_PB0438** | | CDS |  | Hypothetical protein | -5.2 |
| **DGo_CA2183** | | CDS |  | Isoprenylcysteine carboxyl methyltransferase | -5.2 |
| **DGo_CA1728** | | CDS |  | Hypothetical protein | -5.1 |
| **DGo_CA2530** | | CDS |  | Hypothetical protein | -5.1 |
| **DGo_CA2399** | | CDS |  | amino acid ABC transporter, periplasmic amino acid-binding prote in | -5.1 |
| **DGo_CA0750** | | CDS |  | Hypothetical protein | -5.0 |
| **DGo_CA2807** | | CDS | *luxS* | S-ribosylhomocysteine lyase | -5.0 |
| **DGo_CA1987** | | CDS |  | Hypothetical protein | -5.0 |
| **DGo_CA2470** | | CDS | *tolQ* | Biopolymer transport protein, putative | -5.0 |
| **DGo_CA1648** | | CDS |  | Hydrolase, putative | -4.9 |
| **DGo_CA2522** | | CDS |  | Cytidine/deoxycytidylate deaminase/nudix/methyltransferase domains protein | -4.9 |
| **DGo_PB0156** | | CDS |  | Hypothetical protein | -4.8 |
| **DGo_CA0478** | | CDS |  | putative ATP-binding protein | -4.8 |
| **DGo_CA0994** | | CDS | *potC* | Spermidine/putrescine ABC transporter, permease protein | -4.8 |
| **DGo_CA1985** | | CDS |  | Hypothetical protein | -4.8 |
| **DGo_CA2911** | | CDS |  | Ferredoxin | -4.8 |
| **DGo_CA2341** | | CDS |  | Diguanylate cyclase | -4.8 |
| **DGo_CA1973** | | CDS |  | Hypothetical protein | -4.8 |
| **DGo_PF0032** | | CDS |  | Arsenate reductase, ArsC-like protein | -4.7 |
| **DGo_CA2428** | | CDS |  | Arginine repressor | -4.7 |
| **DGo_PA0093** | | CDS |  | Hypothetical protein | -4.7 |
| **DGo_CA2323** | | CDS |  | HAD-superfamily phosphatase subfamily IIIA | -4.6 |
| **DGo_CA0979** | | CDS |  | Guanyl-specific ribonuclease Sa-like protein | -4.6 |
| **DGo_CA0407** | | CDS | *nrdH* | Glutaredoxin 2 | -4.6 |
| **DGo_PC0182** | | CDS |  | Hypothetical protein | -4.6 |
| **DGo_PB0316** | | CDS |  | A Chain A, Crystal Structure Of A Protein With Unknown Function From Duf162 Family | -4.6 |
| **DGo_PC0067** | | CDS |  | Hypothetical protein | -4.6 |
| **DGo_CA0740** | | CDS |  | Two component transcriptional regulator, winged helix family | -4.6 |
| **DGo_CA2173** | | CDS |  | Signal transduction diguanylate cyclase | -4.6 |
| **DGo_PB0476** | | CDS |  | Hypothetical protein | -4.5 |
| **DGo_CA1904** | | CDS |  | Hypothetical protein | -4.5 |
| **DGo_CA2135** | | CDS | *ompH* | Outer membrane chaperone Skp | -4.5 |
| **DGo_PB0310** | | CDS |  | Predicted transporter, permease component | -4.4 |
| **DGo_CA0196** | | CDS |  | RelE/ParE family protein | -4.4 |
| **DGo_PB0077** | | CDS |  | Hypothetical protein | -4.4 |
| **DGo_PB0174** | | CDS |  | Hypothetical protein | -4.4 |
| **DGo_CA1558** | | CDS | *ylmE* | UPF0001 protein | -4.4 |
| **DGo_CA2322** | | CDS |  | UPF0124 protein | -4.4 |
| **DGo_PB0398** | | CDS |  | Transcriptional regulator, PadR-like family | -4.4 |
| **DGo_PB0266** | | CDS |  | Hypothetical protein | -4.3 |
| **DGo_PA0210** | | CDS |  | Acetyltransferase, GNAT family | -4.3 |
| **DGo_PB0483** | | CDS |  | Hypothetical protein | -4.3 |
| **DGo_PA0362** | | CDS |  | Oxidoreductase, short chain dehydrogenase/reductase family | -4.3 |
| **DGo_CA2528** | | CDS |  | Putative kinase protein | -4.3 |
| **DGo_CA2291** | | CDS |  | Pseudouridine synthase, RluA family | -4.3 |
| **DGo_CA1909** | | CDS |  | peptidase domain protein | -4.2 |
| **DGo_CA1031** | | CDS |  | Putative transcriptional regulator | -4.2 |
| **DGo_PA0132** | | CDS |  | Transcriptional regulator, PadR-like family | -4.2 |
| **DGo_CA1255** | | CDS | *nuoK* | NADH dehydrogenase I subunit K | -4.2 |
| **DGo_CA2919** | | CDS |  | Acetyltransferase, gnat family | -4.2 |
| **DGo_PC0270** | | CDS |  | Hypothetical protein | -4.2 |
| **DGo_CA0905** | | CDS |  | Competence protein ComEC/Rec2, putative | -4.1 |
| **DGo_PC0078** | | CDS |  | Hypothetical protein | -4.1 |
| **DGo_CA2554** | | CDS |  | synthase | -4.1 |
| **DGo_CA0744** | | CDS |  | Transcriptional regulator IclR-like protein | -4.1 |
| **DGo_CA1970** | | CDS |  | Hypothetical protein | -4.1 |
| **DGo_CA1254** | | CDS | *nuoJ* | NADH-quinone oxidoreductase, J subunit | -4.1 |
| **DGo_CA1830** | | CDS | *cyp* | Cytochrome P450 | -4.1 |
| **DGo_CA0790** | | CDS |  | Diguanylate cyclase/phosphodiesterase with PAS/PAC sensor | -4.1 |
| **DGo_CA2404** | | CDS |  | FMN-dependent dehydrogenase superfamily | -4.1 |
| **DGo_CA1617** | | CDS |  | NADH-dependent flavin oxidoreductase, putative | -4.1 |
| **DGo_CA0314** | | CDS |  | Putative thiol-specific antioxidant related protein | -4.1 |
| **DGo_PB0473** | | CDS | *terL* | Terminase large subunit | -4.0 |
| **DGo_CA1082** | | CDS |  | Hypothetical protein | -4.0 |
| **DGo_PC0223** | | CDS | *arsR* | Transcriptional regulator, ArsR family | -4.0 |
| **DGo_CA1469** | | CDS |  | Predicted membrane-bound metal-dependent hydrolase | -4.0 |
| **DGo_CA1898** | | CDS |  | Hypothetical protein | -4.0 |
| **DGo_CA0410** | | CDS |  | Hypothetical protein | -4.0 |
| **DGo_PA0126** | | CDS |  | Hypothetical protein | -3.9 |
| **DGo_CA2088** | | CDS | *recX* | Regulatory protein recX | -3.9 |
| **DGo_PC0015** | | CDS |  | Hypothetical protein | -3.9 |
| **DGo_CA0431** | | CDS |  | Hypothetical protein | -3.9 |
| **DGo_PA0118** | | CDS |  | Probable two-component sensor histidine kinase protein | -3.9 |
| **DGo_CA1096** | | CDS |  | Methanol dehydrogenase regulatory protein | -3.9 |
| **DGo_PA0244** | | CDS |  | Hypothetical protein | -3.9 |
| **DGo_PB0336** | | CDS |  | Hypothetical protein | -3.9 |
| **DGo_PA0322** | | CDS |  | Hypothetical protein | -3.8 |
| **DGo_PC0026** | | CDS |  | Hypothetical protein | -3.8 |
| **DGo_CA0762** | | CDS | *ureD* | Urease accessory protein ureD | -3.8 |
| **DGo_PD0010** | | CDS |  | Hypothetical protein | -3.8 |
| **DGo_CA0853** | | CDS |  | Hypothetical protein | -3.8 |
| **DGo_CA0783** | | CDS |  | IclR family transcriptional regulator | -3.7 |
| **DGo_CA2281** | | CDS | *dcd* | dCTP deaminase | -3.7 |
| **DGo_CA1066** | | CDS |  | Chromosome partitioning protein, ParA family | -3.7 |
| **DGo_CA1419** | | CDS |  | Transcriptional regulator, Crp/Fnr family | -3.7 |
| **DGo_PC0188** | | CDS |  | Cell wall hydrolase/autolysin | -3.7 |
| **DGo_PF0001** | | CDS |  | Hypothetical protein | -3.7 |
| **DGo_CA1406** | | CDS | *deoC* | Deoxyribose-phosphate aldolase | -3.7 |
| **DGo_PB0311** | | CDS |  | Hypothetical protein | -3.7 |
| **DGo_CA2847** | | CDS |  | Hypothetical protein | -3.7 |
| **DGo_PA0122** | | CDS |  | Polyphosphate glucokinase | -3.6 |
| **DGo_CA0370** | | CDS |  | Zn-finger containing protein | -3.6 |
| **DGo_CA1095** | | CDS |  | Hypothetical protein | -3.6 |
| **DGo_PC0002** | | CDS |  | Hypothetical protein | -3.6 |
| **DGo_PA0339** | | CDS |  | ABC transporter, ATP-binding protein | -3.6 |
| **DGo_PF0043** | | CDS |  | Hypothetical protein | -3.6 |
| **DGo_CA2502** | | CDS | *nos* | Nitric oxide synthase oxygenase | -3.6 |
| **DGo_CA1374** | | CDS | *minC* | Probable septum site-determining protein minC | -3.6 |
| **DGo_CA1908** | | CDS |  | Hypothetical protein | -3.5 |
| **DGo_CA2217** | | CDS |  | Polysaccharide deacetylase, putative | -3.5 |
| **DGo_PB0168** | | CDS |  | Hypothetical protein | -3.5 |
| **DGo_PA0243** | | CDS |  | Transposase IS-4 | -3.5 |
| **DGo_CA0692** | | CDS |  | Response regulator receiver:Metal-dependent phosphohydrolase, HD subdomain | -3.5 |
| **DGo_PC0193** | | CDS |  | Redoxin | -3.5 |
| **DGo_PA0166** | | CDS |  | ABC-type maltose transport system, periplasmic component, MalE | -3.5 |
| **DGo_CA0807** | | CDS | *cbiG* | Precorrin methylase protein | -3.5 |
| **DGo_PA0190** | | CDS |  | Hypothetical protein | -3.5 |
| **DGo_CA0724** | | CDS |  | Hypothetical protein | -3.5 |
| **DGo_CA2417** | | CDS |  | Hypothetical protein | -3.5 |
| **DGo_CA2171** | | CDS |  | predicted regulor of amylopullulanase | -3.5 |
| **DGo_CA0424** | | CDS | *lmbE* | LmbE-like protein protein | -3.5 |
| **DGo_PA0299** | | CDS |  | Hypothetical protein | -3.4 |
| **DGo_CA0200** | | CDS |  | Thioesterase superfamily | -3.4 |
| **DGo_CA1895** | | CDS |  | Hypothetical protein | -3.4 |
| **DGo_CA1527** | | CDS |  | Hypothetical protein | -3.4 |
| **DGo_CA1062** | | CDS |  | Hypothetical protein | -3.4 |
| **DGo_CA0584** | | CDS |  | Hypothetical protein | -3.4 |
| **DGo_PB0325** | | CDS |  | Transcriptional regulator, LacI family | -3.4 |
| **DGo_PB0146** | | CDS |  | Hypothetical protein | -3.4 |
| **DGo_PC0260** | | CDS |  | Transposase, IS4 | -3.4 |
| **DGo_CA0292** | | CDS |  | Predicted HD phosphohydrolase family protein | -3.4 |
| **DGo_CA1801** | | CDS |  | Hypothetical protein | -3.4 |
| **DGo_CA1888** | | CDS |  | Hypothetical protein | -3.4 |
| **DGo_CA1889** | | CDS |  | Predicted protein | -3.4 |
| **DGo_CA0575** | | CDS |  | Hypothetical protein | -3.4 |
| **DGo_CA0706** | | CDS |  | Putative transcriptional regulator | -3.3 |
| **DGo_PB0436** | | CDS |  | Hypothetical protein | -3.3 |
| **DGo_CA0403** | | CDS | *moeA* | MoeA-like protein, domain I and II | -3.3 |
| **DGo_PB0204** | | CDS |  | Hypothetical protein | -3.3 |
| **DGo_PB0085** | | CDS | *mviM* | Oxidoreductase domain protein | -3.3 |
| **DGo_CA1807** | | CDS | *nth* | Endonuclease III, putative | -3.3 |
| **DGo_CA0472** | | CDS |  | Hypothetical protein | -3.3 |
| **DGo_PB0055** | | CDS |  | Hypothetical protein | -3.3 |
| **DGo_PB0312** | | CDS | *cyp* | Cytochrome P450 51 | -3.3 |
| **DGo_CA0747** | | CDS | *appB* | Peptide ABC transporter, permease protein | -3.3 |
| **DGo_CA0954** | | CDS | *kdtB* | Phosphopantetheine adenylyltransferase | -3.3 |
| **DGo_PC0022** | | CDS |  | Hypothetical protein | -3.3 |
| **DGo_CA1680** | | CDS | *murM* | Peptidoglycan branched peptide synthesis, doubled GNAT-acetyltrasferase fold | -3.3 |
| **DGo_PC0125** | | CDS |  | Hypothetical protein | -3.3 |
| **DGo_CA1201** | | CDS |  | Hypothetical protein | -3.3 |
| **DGo_CA1282** | | CDS |  | putative acetyltransferase | -3.3 |
| **DGo_PB0369** | | CDS |  | Probable insertion sequence transposase protein | -3.3 |
| **DGo_PA0089** | | CDS |  | Hypothetical protein | -3.3 |
| **DGo_PB0447** | | CDS |  | Hypothetical protein | -3.3 |
| **DGo_PB0257** | | CDS |  | Hypothetical protein | -3.3 |
| **DGo_CA2282** | | CDS |  | Hypothetical protein | -3.3 |
| **DGo_CA0702** | | CDS |  | Lea76/Lea29-like desiccation resistance protein | -3.2 |
| **DGo_CA1980** | | CDS |  | Hypothetical protein | -3.2 |
| **DGo_PB0052** | | CDS |  | Arsenite transmembrane pump-like protein | -3.2 |
| **DGo_PC0153** | | CDS |  | Hypothetical protein | -3.2 |
| **DGo_CA1044** | | CDS |  | Roadblock/LC7 | -3.2 |
| **DGo_PB0214** | | CDS |  | putative IS1648 transposase | -3.2 |
| **DGo_PA0027** | | CDS | *cynS* | Cyanate lyase, CynS | -3.2 |
| **DGo_CA0135** | | CDS | *mgsA* | Methylglyoxal synthase protein | -3.2 |
| **DGo_CA2494** | | CDS |  | Serine cycle enzyme, putative | -3.2 |
| **DGo_PB0462** | | CDS |  | Hypothetical protein | -3.2 |
| **DGo_CA1183** | | CDS |  | Alpha/beta superfamily hydrolase | -3.2 |
| **DGo_PB0417** | | CDS |  | Hypothetical protein | -3.2 |
| **DGo_CA0978** | | CDS | *upk* | Undecaprenyl-diphosphatase | -3.2 |
| **DGo_PA0165** | | CDS |  | Glycosyl hydrolase, family 53-likely arabinogalactan 1,4-beta-galactosidase | -3.2 |
| **DGo_CA0227** | | CDS |  | Hypothetical protein | -3.2 |
| **DGo_PA0150** | | CDS | *rbsA* | Ribose ABC transporter permease protein RbsD | -3.2 |
| **DGo_PC0105** | | CDS |  | Hypothetical protein | -3.2 |
| **DGo_PA0081** | | CDS |  | Hypothetical protein | -3.1 |
| **DGo_CA1133** | | CDS |  | Hypothetical protein | -3.1 |
| **DGo_CA1894** | | CDS |  | Hypothetical protein | -3.1 |
| **DGo_PA0256** | | CDS |  | UDP-galactose-lipid carrier transferase | -3.1 |
| **DGo_PB0235** | | CDS |  | Hypothetical protein | -3.1 |
| **DGo_CA1690** | | CDS |  | Hypothetical protein | -3.1 |
| **DGo_CA1547** | | CDS |  | Alpha/beta hydrolase fold-3 | -3.1 |
| **DGo_CA1258** | | CDS | *nuoN* | NADH-quinone oxidoreductase, N subunit | -3.1 |
| **DGo_CA2946** | | CDS |  | Hypothetical protein | -3.1 |
| **DGo_CA2699** | | CDS |  | Phenol hydroxylase | -3.1 |
| **DGo_CA0742** | | CDS |  | Hypothetical protein | -3.1 |
| **DGo_PB0190** | | CDS |  | Hypothetical protein | -3.1 |
| **DGo_CA2090** | | CDS |  | Hypothetical protein | -3.1 |
| **DGo_CA1363** | | CDS |  | ComA like protein, thioesterase superfamily | -3.1 |
| **DGo_CA1534** | | CDS |  | putative Lipoprotein-releasing system ATP-binding protein lolD; putative ABC transporter, ATP-binding component | -3.1 |
| **DGo_PC0108** | | CDS |  | Hypothetical protein | -3.1 |
| **DGo_CA0015** | | CDS |  | Peptidase C39 | -3.1 |
| **DGo_CA1984** | | CDS |  | Hypothetical protein | -3.1 |
| **DGo_PC0020** | | CDS |  | Hypothetical protein | -3.1 |
| **DGo_CA0893** | | CDS |  | Hypothetical protein | -3.1 |
| **DGo_CA0646** | | CDS | *kdpD* | Potassium-transporting ATPase, D subunit | -3.1 |
| **DGo_CA0300** | | CDS |  | Hypothetical protein | -3.0 |
| **DGo_CA2853** | | CDS | *trmH* | RNA methyltransferase, putative | -3.0 |
| **DGo_CA2070** | | CDS |  | Hypothetical protein | -3.0 |
| **DGo_CA0757** | | CDS |  | Hydrogenase expression/formation HypB-related protein | -3.0 |
| **DGo_CA2044** | | CDS |  | Hypothetical protein | -3.0 |
| **DGo_PB0008** | | CDS |  | Hypothetical protein | -3.0 |
| **DGo_CA2613** | | CDS |  | Carbonic anhydrase | -3.0 |
| **DGo_CA0573** | | CDS |  | Response regulator receiver modulated diguanylate cyclase/phosphodiesterase with PAS/PAC sensor | -3.0 |
| **DGo_CA0913** | | CDS | *crt* | Enoyl-CoA hydratase/isomerase | -3.0 |
| **DGo_CA2388** | | CDS | *cysG* | Siroheme synthase | -3.0 |
| **DGo_CA1037** | | CDS | *aacC* | Aminoglycoside N3`-acetyltransferase, type IV | -3.0 |
| **DGo_CA0754** | | CDS |  | Peptidase M29, aminopeptidase II | -3.0 |
| **DGo_CA2450** | | CDS |  | putative VanZ like family protein; putative membrane protein | -3.0 |
| **DGo_PB0469** | | CDS |  | Hypothetical protein | -3.0 |
| **DGo_CA0547** | | CDS |  | UPF0078 membrane protein | -3.0 |
| **DGo_PC0272** | | CDS |  | Hypothetical protein | -3.0 |
| **DGo_PB0272** | | CDS |  | Hypothetical protein | -3.0 |
| **DGo_PB0297** | | CDS |  | Hypothetical protein | -3.0 |
| **DGo_PD0061** | | CDS |  | Hypothetical protein | -3.0 |
| **DGo_CA1859** | | CDS |  | Hypothetical protein | -3.0 |
| **DGo_PB0069** | | CDS |  | Hypothetical protein | -3.0 |
| **DGo_CA2938** | | CDS |  | UPF0189 protein | -3.0 |
| **DGo_PA0140** | | CDS |  | Amidohydrolase family enzyme | -3.0 |
| **DGo_CA1516** | | CDS |  | Hypothetical protein | -2.9 |
| **DGo_PC0192** | | CDS |  | Secreted protein | -2.9 |
| **DGo_CA1771** | | CDS | *pcaC* | 4-carboxymuconolactone decarboxylase | -2.9 |
| **DGo_CA1237** | | CDS |  | N-acetylmuramoyl-L-alanine amidase, family 2 | -2.9 |
| **DGo_CA0025** | | CDS |  | Hypothetical protein | -2.9 |
| **DGo_CA2734** | | CDS |  | putative membrane protein | -2.9 |
| **DGo_PC0038** | | CDS |  | Diguanylate cyclase and metal dependent phosphohydrolase | -2.9 |
| **DGo_CA1251** | | CDS | *nuoG* | NADH-quinone oxidoreductase | -2.9 |
| **DGo_CA0067** | | CDS |  | Metallophosphoesterase | -2.9 |
| **DGo_CA0119** | | CDS |  | Carbohydrate kinase FGGY | -2.9 |
| **DGo_CA0253** | | CDS |  | Hypothetical protein | -2.9 |
| **DGo_CA2752** | | CDS |  | Carbohydrate-binding CenC domain protein | -2.9 |
| **DGo_CA1975** | | CDS |  | Hypothetical protein | -2.9 |
| **DGo_CA1795** | | CDS |  | Hypothetical protein | -2.9 |
| **DGo_CA2249** | | CDS | *aroC* | Chorismate synthase | -2.9 |
| **DGo_PC0278** | | CDS |  | Hypothetical protein | -2.9 |
| **DGo_CA0100** | | CDS |  | Hypothetical protein | -2.9 |
| **DGo_CA1067** | | CDS |  | Hypothetical protein | -2.9 |
| **DGo_PB0418** | | CDS |  | Hypothetical protein | -2.9 |
| **DGo_CA1993** | | CDS |  | Hypothetical protein | -2.9 |
| **DGo_PB0187** | | CDS |  | Hypothetical protein | -2.9 |
| **DGo_CA0013** | | CDS |  | Hypothetical protein | -2.8 |
| **DGo_CA1286** | | CDS |  | Peptidase M29, aminopeptidase II | -2.8 |
| **DGo_PB0185** | | CDS |  | Hypothetical protein | -2.8 |
| **DGo_PB0145** | | CDS |  | Hypothetical protein | -2.8 |
| **DGo_PC0161** | | CDS |  | ParB-like partition protein | -2.8 |
| **DGo_PE0049** | | CDS |  | Hypothetical protein | -2.8 |
| **DGo_CA1574** | | CDS | *mae* | C4-dicarboxylate transporter/malic acid transport protein | -2.8 |
| **DGo_PB0173** | | CDS |  | Predicted membrane-bound metal-dependent hydrolase | -2.8 |
| **DGo_CA1509** | | CDS |  | Hypothetical protein | -2.8 |
| **DGo_CA1494** | | CDS |  | Metallophosphoesterase | -2.8 |
| **DGo_CA1257** | | CDS | *nuoM* | NADH-quinone oxidoreductase, M subunit | -2.8 |
| **DGo_CA2105** | | CDS |  | Hypothetical protein | -2.8 |
| **DGo_CA0369** | | CDS | *marR* | Transcriptional regulator, MarR family | -2.8 |
| **DGo_CA0855** | | CDS |  | Arginase | -2.8 |
| **DGo_PC0111** | | CDS |  | Hypothetical protein | -2.8 |
| **DGo_CA1017** | | CDS |  | NAD-dependent aldehyde dehydrogenase | -2.8 |
| **DGo_CA2611** | | CDS | *rpsO* | 30S ribosomal protein S15 | -2.8 |
| **DGo_CA2682** | | CDS |  | Hypothetical protein | -2.8 |
| **DGo_PA0173** | | CDS |  | Hypothetical protein | -2.8 |
| **DGo_PA0035** | | CDS |  | ABC transporter, permease protein | -2.8 |
| **DGo_PB0038** | | CDS |  | Hypothetical protein | -2.8 |
| **DGo_CA1221** | | CDS |  | Putative DNA-binding protein | -2.8 |
| **DGo_CA0618** | | CDS |  | Glutamine-fructose-6-phosphate transaminase | -2.8 |
| **DGo_CA2541** | | CDS |  | Thioredoxin | -2.8 |
| **DGo_CA2330** | | CDS |  | NADH dehydrogenase | -2.7 |
| **DGo_CA1703** | | CDS | *truD* | tRNA pseudouridine synthase D | -2.7 |
| **DGo_CA1670** | | CDS |  | Hypothetical protein | -2.7 |
| **DGo_PB0236** | | CDS |  | Hypothetical protein | -2.7 |
| **DGo_CA1963** | | CDS |  | Hypothetical protein | -2.7 |
| **DGo_PC0211** | | CDS |  | Glutaredoxin | -2.7 |
| **DGo_CA1796** | | CDS |  | PBP1 protein; transglycosylase, transpeptidase domain | -2.7 |
| **DGo_CA1657** | | CDS | *bmrU* | Diacylglycerol kinase, catalytic region | -2.7 |
| **DGo_CA2862** | | CDS | *purQ* | Phosphoribosylformylglycinamidine synthase, glutamine amidotransferase domain | -2.7 |
| **DGo_CA0465** | | CDS |  | Polyferredoxin, putative | -2.7 |
| **DGo_PB0268** | | CDS |  | Hypothetical protein | -2.7 |
| **DGo_CA1256** | | CDS | *nuoL* | NADH-quinone oxidoreductase, L subunit | -2.7 |
| **DGo_PA0012** | | CDS |  | Hypothetical protein | -2.7 |
| **DGo_CA1464** | | CDS | *cyp* | Cytochrome P450 | -2.7 |
| **DGo_CA1626** | | CDS | *hisZ* | ATP phosphoribosyltransferase, Histidyl-tRNA synthetase-like protein | -2.7 |
| **DGo_CA0667** | | CDS |  | Miro-like protein | -2.7 |
| **DGo_CA1487** | | CDS |  | Spermidine synthase | -2.7 |
| **DGo_CA0026** | | CDS |  | Hypothetical protein | -2.7 |
| **DGo_CA0587** | | CDS |  | Magnesium chelatase, ChlI subunit | -2.7 |
| **DGo_CA1569** | | CDS | *pdxT* | Glutamine amidotransferase subunit pdxT | -2.7 |
| **DGo_CA2141** | | CDS |  | Putative transmembrane protein | -2.7 |
| **DGo_PB0466** | | CDS |  | Hypothetical protein | -2.7 |
| **DGo_CA1447** | | CDS | *trpG* | Glutamine amidotransferase of anthranilate synthase or para-aminobenzoate synthase | -2.7 |
| **DGo_CA2495** | | CDS |  | Glycosyl transferase, group 1 | -2.7 |
| **DGo_PB0501** | | CDS |  | Hypothetical protein | -2.7 |
| **DGo_CA2937** | | CDS |  | Hypothetical protein | -2.7 |
| **DGo_CA0635** | | CDS |  | Permease protein, ABC-type nitrate/sulfonate/taurine/bicarbonate transporter | -2.7 |
| **DGo_PA0186** | | CDS |  | ABC-type transport system for cytochrome bd biosynthesis, ATPase and permease component | -2.7 |
| **DGo_CA0150** | | CDS |  | Hypothetical protein | -2.7 |
| **DGo_PA0181** | | CDS |  | Alpha/beta hydrolase fold | -2.7 |
| **DGo_CA0809** | | CDS | *cobW* | Cobalamin synthesis protein, GTPase of G3E family | -2.7 |
| **DGo_CA1741** | | CDS | *nadE* | NAD synthetase, NH3/glutamine-dependent | -2.7 |
| **DGo_CA1760** | | CDS |  | TRAP transporter solute receptor, TAXI family | -2.7 |
| **DGo_PB0113** | | CDS |  | Transposase, IS4 | -2.7 |
| **DGo_CA0324** | | CDS |  | Aminodeoxychorismate lyase | -2.6 |
| **DGo_CA1827** | | CDS | *sbcC* | SbcC, ATPase involved in DNA repair | -2.6 |
| **DGo_PA0307** | | CDS | *ams* | Glycoside hydrolase family 38 | -2.6 |
| **DGo_CA1267** | | CDS |  | multi-sensor signal transduction histidine kinase | -2.6 |
| **DGo_PC0247** | | CDS |  | Hypothetical protein | -2.6 |
| **DGo_PB0192** | | CDS | *cas2* | Crispr-associated protein cas2 | -2.6 |
| **DGo_PC0136** | | CDS |  | Hypothetical protein | -2.6 |
| **DGo_PB0430** | | CDS |  | Hypothetical protein | -2.6 |
| **DGo_CA1016** | | CDS | *yqjF* | YqjF | -2.6 |
| **DGo_PB0498** | | CDS |  | Hypothetical protein | -2.6 |
| **DGo_PC0007** | | CDS |  | Hypothetical protein | -2.6 |
| **DGo_CA0940** | | CDS |  | Hypothetical protein | -2.6 |
| **DGo_PE0054** | | CDS |  | Hypothetical protein | -2.6 |
| **DGo_CA0525** | | CDS |  | Cation transport system protein, putative | -2.6 |
| **DGo_PA0234** | | CDS |  | Hypothetical protein | -2.6 |
| **DGo_PC0145** | | CDS |  | Hypothetical protein | -2.6 |
| **DGo_PB0130** | | CDS |  | Hypothetical protein | -2.6 |
| **DGo_PB0287** | | CDS |  | Hypothetical protein | -2.6 |
| **DGo_CA0043** | | CDS |  | Hypothetical protein | -2.6 |
| **DGo_CA0115** | | CDS |  | Hypothetical protein | -2.6 |
| **DGo_PF0028** | | CDS |  | Hypothetical protein | -2.6 |
| **DGo_CA1179** | | CDS |  | NADH-quinone oxidoreductase subunit 15 | -2.6 |
| **DGo_CA0612** | | CDS |  | TetR family protein transcriptional regulator | -2.6 |
| **DGo_PB0304** | | CDS |  | Arsenate reductase, ArsC-like protein | -2.6 |
| **DGo_CA0637** | | CDS |  | ABC transporter, nucleotide binding/ATPase protein | -2.6 |
| **DGo_CA0718** | | CDS | *rbsC* | Ribose/xylose/arabinose/galactoside ABC-type transport systems, permease components | -2.6 |
| **DGo_PC0227** | | CDS |  | Transposase, IS4 family protein | -2.6 |
| **DGo_CA0801** | | CDS |  | Hypothetical protein | -2.6 |
| **DGo_CA2780** | | CDS |  | Hypothetical protein | -2.6 |
| **DGo_CA1783** | | CDS |  | Hypothetical protein | -2.6 |
| **DGo_PA0278** | | CDS | *pdxY* | Pyridoxamine kinase | -2.6 |
| **DGo_CA1236** | | CDS |  | Ribonuclease P protein component | -2.5 |
| **DGo_PC0156** | | CDS |  | Hypothetical protein | -2.5 |
| **DGo_PA0318** | | CDS |  | Xanthine/uracil/vitamin C permease | -2.5 |
| **DGo_CA0743** | | CDS |  | cobalt dependent X-Pro dipeptidase | -2.5 |
| **DGo_CA1992** | | CDS |  | Hypothetical protein | -2.5 |
| **DGo_PA0280** | | CDS |  | Glycoside hydrolase family 16 | -2.5 |
| **DGo_PB0015** | | CDS | *uvrD3* | UvrD/REP helicase | -2.5 |
| **DGo_CA0155** | | CDS | *ugpQ* | Glycerophosphoryl diester phosphodiesterase | -2.5 |
| **DGo_CA2672** | | CDS | *hemC* | Porphobilinogen deaminase | -2.5 |
| **DGo_PA0128** | | CDS |  | Hypothetical protein | -2.5 |
| **DGo_CA2086** | | CDS |  | Peptidase M55, D-aminopeptidase | -2.5 |
| **DGo_CA0777** | | CDS |  | Creatininase subfamily protein | -2.5 |
| **DGo_CA2492** | | CDS |  | Hypothetical protein | -2.5 |
| **DGo_CA1252** | | CDS | *nuoH* | NADH-quinone oxidoreductase subunit H | -2.5 |
| **DGo_PA0156** | | CDS |  | Hypothetical protein | -2.5 |
| **DGo_PB0454** | | CDS |  | Hypothetical protein | -2.5 |
| **DGo_PA0134** | | CDS | *splB* | putative spore photoproduct lyase | -2.5 |
| **DGo_CA2584** | | CDS | *rpsC* | 30S ribosomal protein S3 | -2.5 |
| **DGo_CA2778** | | CDS |  | Restriction endonuclease S subunits | -2.5 |
| **DGo_CA1775** | | CDS |  | Threonyl/alanyl tRNA synthetase, SAD | -2.5 |
| **DGo_CA2420** | | CDS |  | Peptidase S1 and S6, chymotrypsin/Hap | -2.5 |
| **DGo_PB0499** | | CDS |  | Histone acetyltransferase HPA10 | -2.5 |
| **DGo_PB0184** | | CDS |  | Hypothetical protein | -2.5 |
| **DGo_CA2845** | | CDS |  | methyltransferase | -2.5 |
| **DGo_PB0153** | | CDS |  | Hypothetical protein | -2.5 |
| **DGo_PB0160** | | CDS |  | Hypothetical protein | -2.5 |
| **DGo_PB0136** | | CDS |  | Hypothetical protein | -2.5 |
| **DGo_CA2849** | | CDS |  | Hypothetical protein | -2.5 |
| **DGo_CA1869** | | CDS |  | Hypothetical protein | -2.5 |
| **DGo_CA2276** | | CDS |  | Thioesterase superfamily | -2.5 |
| **DGo_CA1328** | | CDS |  | Hypothetical protein | -2.5 |
| **DGo_CA2678** | | CDS | *purK* | Phosphoribosylaminoimidazole carboxylase, ATPase subunit | -2.5 |
| **DGo_CA0746** | | CDS |  | ABC transporter, substrate binding protein | -2.5 |
| **DGo_PB0161** | | CDS |  | Hypothetical protein | -2.5 |
| **DGo_CA2945** | | CDS |  | Hypothetical protein | -2.5 |
| **DGo_CA0698** | | CDS | *ugpB* | ABC-type sugar transport system, periplasmic component | -2.5 |
| **DGo_PB0209** | | CDS |  | Hypothetical protein | -2.5 |
| **DGo_PA0183** | | CDS | *yjhC* | Oxidoreductase domain protein | -2.5 |
| **DGo_CA1897** | | CDS |  | Hypothetical protein | -2.5 |
| **DGo_PA0022** | | CDS |  | Hypothetical protein | -2.5 |
| **DGo_PA0114** | | CDS |  | Hypothetical protein | -2.5 |
| **DGo_PA0059** | | CDS |  | ABC transporter, ATP-binding protein, MsbA family | -2.5 |
| **DGo_CA2690** | | CDS |  | FOG: CheY-like receiver | -2.5 |
| **DGo_PD0055** | | CDS |  | Resolvase | -2.5 |
| **DGo_PA0254** | | CDS |  | Hypothetical protein | -2.4 |
| **DGo_CA0176** | | CDS |  | Hypothetical protein | -2.4 |
| **DGo_CA2136** | | CDS | *ompH* | Cationic outer membrane protein OmpH, putative | -2.4 |
| **DGo_CA1332** | | CDS |  | Lincosamide nucleotidyltransferase | -2.4 |
| **DGo_PB0480** | | CDS |  | Hypothetical protein | -2.4 |
| **DGo_PA0365** | | CDS |  | Alpha/beta hydrolase fold-3 domain protein | -2.4 |
| **DGo_PB0486** | | CDS |  | Hypothetical protein | -2.4 |
| **DGo_CA0141** | | CDS |  | Dihydrouridine synthase, DuS | -2.4 |
| **DGo_PC0068** | | CDS |  | transcriptional regulator | -2.4 |
| **DGo_PB0011** | | CDS |  | Hypothetical protein | -2.4 |
| **DGo_CA2237** | | CDS |  | TRAP-T family transporter, periplasmic binding protein | -2.4 |
| **DGo_CA1886** | | CDS |  | Hypothetical protein | -2.4 |
| **DGo_CA2514** | | CDS |  | Hypothetical protein | -2.4 |
| **DGo_PB0423** | | CDS |  | Hypothetical protein | -2.4 |
| **DGo_CA0247** | | CDS |  | Transcriptional regulator, GntR family/aminotransferase family protein | -2.4 |
| **DGo_CA1291** | | CDS | *yqfG* | Putative metalloprotease | -2.4 |
| **DGo_CA1863** | | CDS |  | Pyrroline-5-carboxylate reductase | -2.4 |
| **DGo_CA0677** | | CDS |  | transcriptional regulator, MarR family | -2.4 |
| **DGo_PA0006** | | CDS |  | Hypothetical protein | -2.4 |
| **DGo_CA1563** | | CDS |  | Hypothetical protein | -2.4 |
| **DGo_PC0271** | | CDS |  | Hypothetical protein | -2.4 |
| **DGo_CA0672** | | CDS |  | Hypothetical protein | -2.4 |
| **DGo_PA0267** | | CDS |  | Probable amino acid ABC transporter, permease protein | -2.4 |
| **DGo_PA0269** | | CDS |  | Probable amino acid ABC transporter, substrate-binding protein | -2.4 |
| **DGo_CA0372** | | CDS |  | Ferric enterobactin esterase-related protein, alpha-beta hydrolase superfamily | -2.4 |
| **DGo_CA2581** | | CDS | *rpsQ* | 30S ribosomal protein S17 | -2.4 |
| **DGo_CA1633** | | CDS |  | Glycosyl hydrolase, family 13 | -2.4 |
| **DGo_PA0340** | | CDS |  | Acetyltransferase, GNAT family | -2.4 |
| **DGo_PA0219** | | CDS |  | Hypothetical protein | -2.4 |
| **DGo_PC0220** | | CDS | *arsR* | Transcriptional regulator, ArsR family | -2.4 |
| **DGo_CA2113** | | CDS |  | Hypothetical protein | -2.4 |
| **DGo_CA1689** | | CDS |  | Hypothetical protein | -2.4 |
| **DGo_CA1277** | | CDS |  | HpaII-like repair protein | -2.4 |
| **DGo_CA1580** | | CDS |  | Aminotransferase, class V | -2.4 |
| **DGo_PB0465** | | CDS |  | Hypothetical protein | -2.4 |
| **DGo_CA1835** | | CDS |  | Protein kinase, ArgK family | -2.4 |
| **DGo_CA2441** | | CDS |  | Roadblock/LC7 | -2.4 |
| **DGo_CA2506** | | CDS |  | Amylo-alpha-1,6-glucosidase | -2.4 |
| **DGo_CA0711** | | CDS |  | multi-sensor signal transduction histidine kinase | -2.3 |
| **DGo_PA0324** | | CDS |  | Xylose isomerase domain protein TIM barrel | -2.3 |
| **DGo_PC0263** | | CDS | *recQ4* | ATP-dependent DNA helicase RecQ | -2.3 |
| **DGo_CA1362** | | CDS |  | Peptidase M23B | -2.3 |
| **DGo_PC0186** | | CDS |  | Integrase, catalytic region | -2.3 |
| **DGo_PB0424** | | CDS |  | Hypothetical protein | -2.3 |
| **DGo_PA0336** | | CDS |  | Outer membrane protein | -2.3 |
| **DGo_CA1273** | | CDS |  | Phosphoesterase, RecJ-like protein | -2.3 |
| **DGo_CA0393** | | CDS | *hit* | Histidine triad protein | -2.3 |
| **DGo_PC0197** | | CDS |  | O-methyltransferase domain protein | -2.3 |
| **DGo_CA2109** | | CDS |  | transcriptional regulator | -2.3 |
| **DGo_PB0451** | | CDS |  | Glycoside hydrolase, family 19 | -2.3 |
| **DGo_CA1821** | | CDS | *phnP* | Beta-lactamase-like protein | -2.3 |
| **DGo_PB0133** | | CDS |  | Hypothetical protein | -2.3 |
| **DGo_CA2730** | | CDS |  | E3 binding | -2.3 |
| **DGo_CA1250** | | CDS | *nuoF* | NADH-quinone oxidoreductase, F subunit | -2.3 |
| **DGo_CA2645** | | CDS | *fabG* | Short-chain dehydrogenase/reductase SDR | -2.3 |
| **DGo_CA0261** | | CDS |  | Zinc metallohydrolase, glyoxalase II family | -2.3 |
| **DGo_PB0179** | | CDS |  | Hypothetical protein | -2.3 |
| **DGo_CA1194** | | CDS |  | Peptidase C39 | -2.3 |
| **DGo_PC0262** | | CDS |  | Hypothetical protein | -2.3 |
| **DGo_PB0468** | | CDS |  | Hypothetical protein | -2.3 |
| **DGo_CA0758** | | CDS | *hypA* | Probable hydrogenase nickel incorporation protein hypA | -2.3 |
| **DGo_PC0085** | | CDS |  | Hypothetical protein | -2.3 |
| **DGo_CA0418** | | CDS | *thiN* | Thiamine pyrophosphokinase | -2.3 |
| **DGo_PA0062** | | CDS |  | Transcriptional regulator, GntR family | -2.3 |
| **DGo_PC0173** | | CDS |  | Heavy metal translocating P-type ATPase | -2.3 |
| **DGo_CA0704** | | CDS | *aqpZ* | Glycerol uptake facilitator GlpF, MIP/aquaporin family of transporters | -2.3 |
| **DGo_CA0739** | | CDS |  | sensor histidine kinase, copper metabolism, putative | -2.3 |
| **DGo_CA1572** | | CDS | *lysC* | Aspartokinase | -2.3 |
| **DGo_CA1060** | | CDS |  | Hypothetical protein | -2.3 |
| **DGo_PC0058** | | CDS |  | Hypothetical protein | -2.3 |
| **DGo_CA1202** | | CDS | *moaB* | Molybdenum cofactor biosynthesis protein B | -2.3 |
| **DGo_PC0023** | | CDS |  | UBA/THIF-type NAD/FAD binding protein | -2.3 |
| **DGo_PA0229** | | CDS | *aceB* | Malate synthase | -2.3 |
| **DGo_PE0028** | | CDS |  | Hypothetical protein | -2.3 |
| **DGo_PE0051** | | CDS |  | Hypothetical protein | -2.3 |
| **DGo_PB0461** | | CDS |  | Hypothetical protein | -2.3 |
| **DGo_CA1097** | | CDS |  | Hypothetical protein | -2.3 |
| **DGo_CA2351** | | CDS | *sigK* | RNA polymerase, sigma-24 subunit, ECF subfamily | -2.3 |
| **DGo_CA1701** | | CDS | *rimM* | Ribosome maturation factor rimM | -2.3 |
| **DGo_CA1426** | | CDS |  | Hypothetical protein | -2.3 |
| **DGo_CA2882** | | CDS |  | Hypothetical protein | -2.3 |
| **DGo_CA0821** | | CDS |  | Hypothetical protein | -2.3 |
| **DGo_CA0723** | | CDS |  | Lipoprotein | -2.3 |
| **DGo_PB0343** | | CDS |  | ATPase, histidine kinase-, DNA gyrase B-, and HSP90-like domain protein | -2.3 |
| **DGo_CA1562** | | CDS | *ileS* | Isoleucyl-tRNA synthetase | -2.3 |
| **DGo_CA2362** | | CDS |  | Hypothetical protein | -2.3 |
| **DGo_CA0047** | | CDS |  | Hypothetical protein | -2.3 |
| **DGo_PB0434** | | CDS |  | Zn-dependent peptidase | -2.3 |
| **DGo_CA1253** | | CDS | *nuoI* | NADH-quinone oxidoreductase subunit I | -2.3 |
| **DGo_CA1195** | | CDS |  | Putative membrane protein | -2.3 |
| **DGo_PB0295** | | CDS |  | Hypothetical protein | -2.3 |
| **DGo_PD0069** | | CDS |  | Hypothetical protein | -2.2 |
| **DGo_CA2680** | | CDS |  | Lipid A disaccharide synthase related enzyme | -2.2 |
| **DGo_CA2931** | | CDS |  | PPC, peptidase containing PKD repeats | -2.2 |
| **DGo_CA2852** | | CDS | *ispF* | 2-C-methyl-D-erythritol 2,4-cyclodiphosphate synthase | -2.2 |
| **DGo_CA1789** | | CDS |  | Amino acid ABC transporter, permease protein | -2.2 |
| **DGo_PB0306** | | CDS |  | Transposase IS4 | -2.2 |
| **DGo_CA2951** | | CDS |  | Hypothetical protein | -2.2 |
| **DGo_PD0017** | | CDS |  | Phage integrase family protein | -2.2 |
| **DGo_CA1341** | | CDS |  | Extracellular solute-binding protein family 1 | -2.2 |
| **DGo_CA0778** | | CDS | *apbA* | Ketopantoate reductase ApbA/PanE | -2.2 |
| **DGo_CA1032** | | CDS | *minD* | MinD family ATPase, Mrp | -2.2 |
| **DGo_CA0114** | | CDS |  | Hypothetical protein | -2.2 |
| **DGo_CA0720** | | CDS | *lacI* | Sugar binding transcriptional regulator, LacI family | -2.2 |
| **DGo_CA1926** | | CDS |  | Hypothetical protein | -2.2 |
| **DGo_PB0354** | | CDS |  | Hypothetical protein | -2.2 |
| **DGo_CA0674** | | CDS |  | Serine phosphatase RsbU, regulator of sigma subunit | -2.2 |
| **DGo_PB0058** | | CDS |  | Hypothetical protein | -2.2 |
| **DGo_CA1186** | | CDS |  | Peptidase S1 and S6, chymotrypsin/Hap | -2.2 |
| **DGo_PC0133** | | CDS |  | Hypothetical protein | -2.2 |
| **DGo_CA2455** | | CDS |  | Hypothetical protein | -2.2 |
| **DGo_PB0517** | | CDS |  | Hypothetical protein | -2.2 |
| **DGo_PD0070** | | CDS |  | Hypothetical protein | -2.2 |
| **DGo_CA1230** | | CDS |  | Glycerol-3-phosphate ABC transporter, periplasmic glycerol-3-phosphate-binding protein | -2.2 |
| **DGo_PB0242** | | CDS |  | Type IV secretory pathway VirB4 component, ATPase TRAC | -2.2 |
| **DGo_CA2633** | | CDS | *pilT* | Tfp pilus assembly protein, pilus retraction ATPase PilT | -2.2 |
| **DGo_CA2788** | | CDS |  | Hypothetical protein | -2.2 |
| **DGo_PB0410** | | CDS |  | Hypothetical protein | -2.2 |
| **DGo_CA0206** | | CDS |  | Hypothetical protein | -2.2 |
| **DGo_PB0096** | | CDS |  | Hypothetical protein | -2.2 |
| **DGo_CA2435** | | CDS |  | Hypothetical protein | -2.2 |
| **DGo_CA1523** | | CDS | *deoD* | Purine nucleoside phosphorylase deoD-type | -2.2 |
| **DGo_PA0019** | | CDS |  | Hypothetical protein | -2.2 |
| **DGo_CA2443** | | CDS |  | Hypothetical protein | -2.2 |
| **DGo_PB0037** | | CDS |  | Integrase, catalytic region | -2.2 |
| **DGo_CA0761** | | CDS | *ureG* | Urease accessory protein ureG | -2.2 |
| **DGo_CA0458** | | CDS |  | Glucose-6-phosphate 1-dehydrogenase | -2.2 |
| **DGo_PB0285** | | CDS |  | Hypothetical protein | -2.2 |
| **DGo_PA0382** | | CDS | *cobP* | Adenosyl cobinamide kinase/adenosyl cobinamide phosphate guanylyltransferase, CobU | -2.2 |
| **DGo_PB0364** | | CDS |  | Hypothetical protein | -2.2 |
| **DGo_CA1260** | | CDS |  | Hypothetical protein | -2.2 |
| **DGo_CA2697** | | CDS |  | ABC-type multidrug transport system, permease component | -2.2 |
| **DGo_CA0589** | | CDS | *cobN* | Cobaltochelatase, CobN subunit | -2.2 |
| **DGo_PA0141** | | CDS |  | Trap-t family transporter, periplasmic binding protein | -2.2 |
| **DGo_CA2248** | | CDS |  | Shikimate kinase | -2.2 |
| **DGo_CA1185** | | CDS |  | Transcriptional regulator, LuxR family | -2.2 |
| **DGo_CA0613** | | CDS | *rpiR* | Transcriptional regulator, RpiR family | -2.2 |
| **DGo_PC0130** | | CDS |  | Hypothetical protein | -2.2 |
| **DGo_CA2460** | | CDS |  | Hypothetical protein | -2.2 |
| **DGo_PB0375** | | CDS |  | Transposase IS4 family protein | -2.2 |
| **DGo_CA2696** | | CDS |  | Putative signal transduction histidine kinase | -2.2 |
| **DGo_CA1549** | | CDS |  | Beta-lactamase-like protein | -2.2 |
| **DGo_PB0086** | | CDS |  | Xylose isomerase domain protein TIM barrel | -2.2 |
| **DGo_PB0383** | | CDS |  | Hypothetical protein | -2.2 |
| **DGo_PA0321** | | CDS |  | ParB-like partition protein | -2.2 |
| **DGo_CA2182** | | CDS |  | Orotate phosphoribosyltransferase related protein | -2.2 |
| **DGo_PC0021** | | CDS |  | Hypothetical protein | -2.2 |
| **DGo_CA0539** | | CDS | *oxyR* | Transcriptional regulator, LysR family | -2.2 |
| **DGo_PC0162** | | CDS |  | Hypothetical protein | -2.2 |
| **DGo_PE0027** | | CDS |  | Hypothetical protein | -2.2 |
| **DGo_CA2572** | | CDS | *rpmD* | 50S ribosomal protein L30 | -2.2 |
| **DGo_CA2241** | | CDS |  | UDP-N-acetylmuramoylalanine--D-glutamate ligase | -2.2 |
| **DGo_CA1684** | | CDS |  | Hypothetical protein | -2.2 |
| **DGo_CA2683** | | CDS |  | Hypothetical protein | -2.1 |
| **DGo_PA0368** | | CDS | *appD* | oligopeptide/dipeptide ABC transporter, ATPase subunit | -2.1 |
| **DGo_CA1430** | | CDS |  | 2-oxoacid dehydrogenase | -2.1 |
| **DGo_CA0830** | | CDS |  | VanW-like protein | -2.1 |
| **DGo_CA0435** | | CDS |  | 5-formyltetrahydrofolate cyclo-ligase | -2.1 |
| **DGo_CA1933** | | CDS |  | Hypothetical protein | -2.1 |
| **DGo_CA0248** | | CDS | *benE* | Benzoate membrane transport protein, putative | -2.1 |
| **DGo_CA0203** | | CDS |  | Hypothetical protein | -2.1 |
| **DGo_CA2606** | | CDS | *mntA* | ABC-type Mn2+ transport system, ATPase component | -2.1 |
| **DGo_CA2932** | | CDS |  | Dipeptidyl peptidase IV-related protein | -2.1 |
| **DGo_CA2751** | | CDS |  | Hypothetical protein | -2.1 |
| **DGo_CA0614** | | CDS |  | Periplasmic sugar-binding protein of sugar ABC transporter | -2.1 |
| **DGo_PB0439** | | CDS |  | Hypothetical protein | -2.1 |
| **DGo_PA0383** | | CDS |  | Cobyric acid synthase CobQ | -2.1 |
| **DGo_CA1302** | | CDS |  | Hydroxypyruvate reductase TtuD | -2.1 |
| **DGo_PD0015** | | CDS |  | Hypothetical protein | -2.1 |
| **DGo_CA1936** | | CDS |  | Hypothetical protein | -2.1 |
| **DGo_CA0697** | | CDS | *malF* | ABC-type sugar transport system, permease component | -2.1 |
| **DGo_CA1340** | | CDS | *agaL* | Glycoside hydrolase family 4 | -2.1 |
| **DGo_CA0494** | | CDS |  | Hypothetical protein | -2.1 |
| **DGo_CA2823** | | CDS |  | Hypothetical protein | -2.1 |
| **DGo_CA2632** | | CDS |  | Phosphotransferase system, fructose IIC component | -2.1 |
| **DGo_CA2636** | | CDS |  | NIF3 family protein, DUF34 | -2.1 |
| **DGo_CA0448** | | CDS |  | Rhomboid-like protein protein | -2.1 |
| **DGo_CA1721** | | CDS |  | Acetylornithine and succinylornithine aminotransferase | -2.1 |
| **DGo_CA0902** | | CDS |  | 2-phosphoglycerate kinase, putative | -2.1 |
| **DGo_CA2026** | | CDS |  | Transcriptional regulator, AsnC family | -2.1 |
| **DGo_PA0008** | | CDS |  | Hypothetical protein | -2.1 |
| **DGo_PB0049** | | CDS |  | Hypothetical protein | -2.1 |
| **DGo_PC0118** | | CDS |  | Hypothetical protein | -2.1 |
| **DGo_PA0310** | | CDS |  | Allophanate hydrolase subunit 2 | -2.1 |
| **DGo_PA0142** | | CDS | *pheA* | prephenate dehydratase | -2.1 |
| **DGo_CA2216** | | CDS |  | Major facilitator superfamily MFS_1 | -2.1 |
| **DGo_PA0025** | | CDS |  | Putative nitrate ABC transporter | -2.1 |
| **DGo_CA0271** | | CDS |  | ABC-type branched-chain amino acid transport system, periplasmic component | -2.1 |
| **DGo_PE0011** | | CDS |  | Hypothetical protein | -2.1 |
| **DGo_CA1768** | | CDS | *lytB* | 4-hydroxy-3-methylbut-2-enyl diphosphate reductase | -2.1 |
| **DGo_PB0389** | | CDS |  | Hypothetical protein | -2.1 |
| **DGo_CA0787** | | CDS | *hupA2* | Signal transduction histidine kinase with CheB and CheR activity | -2.1 |
| **DGo_CA1020** | | CDS |  | Hypothetical protein | -2.1 |
| **DGo_PC0148** | | CDS |  | Hypothetical protein | -2.1 |
| **DGo_CA1930** | | CDS |  | Oxidoreductase, aldo/keto reductase family protein | -2.1 |
| **DGo_CA1969** | | CDS |  | Hypothetical protein | -2.1 |
| **DGo_CA1544** | | CDS |  | Hypothetical protein | -2.1 |
| **DGo_CA1342** | | CDS |  | Putative ABC-type sugar transport system, permease component | -2.1 |
| **DGo_PE0063** | | CDS |  | Hypothetical protein | -2.1 |
| **DGo_CA0656** | | CDS |  | Imidazolonepropionase | -2.1 |
| **DGo_CA1035** | | CDS |  | putative Glutamate--ammonia ligase | -2.1 |
| **DGo_CA0037** | | CDS |  | Metalloenzyme, phosphoglyceromutase related | -2.1 |
| **DGo_CA2625** | | CDS |  | GTPase, G3E family | -2.1 |
| **DGo_CA0533** | | CDS |  | 5-formyltetrahydrofolate cyclo-ligase | -2.1 |
| **DGo_PA0292** | | CDS |  | Peptidase C39 | -2.1 |
| **DGo_CA0179** | | CDS | *murE* | UDP-N-acetylmuramyl-tripeptide synthetase | -2.1 |
| **DGo_CA1408** | | CDS |  | Hypothetical protein | -2.1 |
| **DGo_PB0308** | | CDS |  | Hypothetical protein | -2.1 |
| **DGo_CA1249** | | CDS | *nuoE* | NADH-quinone oxidoreductase, E subunit | -2.1 |
| **DGo_CA0106** | | CDS |  | Oxidoreductase, putative | -2.1 |
| **DGo_CA0436** | | CDS |  | Acetyltransferase-like protein | -2.1 |
| **DGo_CA1663** | | CDS |  | Predicted Fe-S-cluster containing protein, UPF0153 | -2.1 |
| **DGo_PB0061** | | CDS |  | Hypothetical protein | -2.1 |
| **DGo_CA0965** | | CDS | *glcF* | Glycolate oxidase, iron-sulfur subunit | -2.1 |
| **DGo_CA0808** | | CDS | *cbiJ* | Probable bifunctional: precorrin-3 methyltransferase and precorrin-6x reductase oxidoreductase protein | -2.1 |
| **DGo_CA0810** | | CDS | *yahD* | Putative transcription factor | -2.1 |
| **DGo_CA1751** | | CDS |  | Hypothetical protein | -2.1 |
| **DGo_CA1686** | | CDS | *smpB* | SsrA-binding protein | -2.1 |
| **DGo_CA0600** | | CDS |  | Secreted protein | -2.1 |
| **DGo_CA0417** | | CDS |  | Alpha/beta superfamily hydrolase | -2.1 |
| **DGo_CA1907** | | CDS |  | Hypothetical protein | -2.1 |
| **DGo_CA1107** | | CDS |  | Deoxyguanosinetriphosphate triphosphohydrolase-like protein | -2.1 |
| **DGo_CA2623** | | CDS |  | PaaI-like thioesterase | -2.1 |
| **DGo_CA1231** | | CDS |  | Hypothetical protein | -2.1 |
| **DGo_CA0124** | | CDS |  | Transcriptional regulator, MerR family | -2.1 |
| **DGo_PA0017** | | CDS |  | Methyl-accepting chemotaxis-related protein | -2.1 |
| **DGo_PA0088** | | CDS |  | Hypothetical protein | -2.1 |
| **DGo_CA2056** | | CDS |  | Acetyltransferase-like protein | -2.1 |
| **DGo_PA0320** | | CDS |  | ATPase involved in plasmide/chromosome partitioning, ParA/Soj-like protein | -2.1 |
| **DGo_CA0951** | | CDS |  | Peptide ABC transporter, periplasmic peptide-binding protein, putative | -2.1 |
| **DGo_CA2278** | | CDS |  | Hypothetical protein | -2.1 |
| **DGo_PD0043** | | CDS |  | Superfamily I DNA and RNA helicase | -2.1 |
| **DGo_CA1643** | | CDS |  | GCN5-related N-acetyltransferase | -2.0 |
| **DGo_CA0903** | | CDS | *tetR* | Transcriptional regulator, TetR family | -2.0 |
| **DGo_PC0088** | | CDS |  | Hypothetical protein | -2.0 |
| **DGo_CA1823** | | CDS | *hpt* | Hypoxanthine phosphoribosyltransferase | -2.0 |
| **DGo_PB0218** | | CDS |  | Plasmid replication initiator repA-related protein | -2.0 |
| **DGo_PB0271** | | CDS |  | Hypothetical protein | -2.0 |
| **DGo_CA2787** | | CDS |  | RelA/SpoT domain protein | -2.0 |
| **DGo_CA2403** | | CDS |  | Aminotransferase, putative | -2.0 |
| **DGo_CA1477** | | CDS |  | Hypothetical protein | -2.0 |
| **DGo_PC0008** | | CDS |  | Type I restriction-modification deoxyribonuclease, R subunit | -2.0 |
| **DGo_CA0040** | | CDS |  | Iron dependent repressor, putative | -2.0 |
| **DGo_PB0178** | | CDS |  | Hypothetical protein | -2.0 |
| **DGo_CA2123** | | CDS |  | YciI-like protein | -2.0 |
| **DGo_PB0446** | | CDS |  | Hypothetical protein | -2.0 |
| **DGo_PB0090** | | CDS |  | ABC ribose transporter, permease component | -2.0 |
| **DGo_CA0338** | | CDS |  | Methlytransferase, UbiE/COQ5 family | -2.0 |
| **DGo_CA2252** | | CDS | *pilO* | Type IV pilus assembly protein PilO | -2.0 |
| **DGo_CA1598** | | CDS |  | proline dipeptidase | -2.0 |
| **DGo_PD0041** | | CDS |  | Helicase domain protein | -2.0 |
| **DGo_CA2103** | | CDS | *rarA* | AAA ATPase, central region | -2.0 |
| **DGo_CA1148** | | CDS |  | Sugar ABC transporter, permease protein | -2.0 |
| **DGo_CA0226** | | CDS |  | Hypothetical protein | -2.0 |
| **DGo_CA1777** | | CDS | *pgd* | 6-phosphogluconate dehydrogenase | -2.0 |
| **DGo_PA0018** | | CDS |  | CheA-related protein | -2.0 |
| **DGo_CA2863** | | CDS |  | Hypothetical protein | -2.0 |
| **DGo_CA1685** | | CDS |  | Cell wall hydrolase/autolysin | -2.0 |
| **DGo_CA1395** | | CDS |  | Hypothetical protein | -2.0 |
| **DGo_CA1336** | | CDS |  | Phosphohydrolase | -2.0 |
| **DGo_PB0457** | | CDS |  | Hypothetical protein | -2.0 |
| **DGo_CA0167** | | CDS | *ynfL* | Transcriptional regulator, LysR family | -2.0 |
| **DGo_PB0322** | | CDS |  | Hypothetical protein | -2.0 |
| **DGo_PA0137** | | CDS |  | Hypothetical protein | -2.0 |
| **DGo_CA2124** | | CDS |  | Phosphoprotein phosphatase | -2.0 |
| **DGo_CA0730** | | CDS |  | Two component transcriptional regulator, winged helix family | -2.0 |
| **DGo_CA2423** | | CDS |  | RNA methyltransferase TrmH, group 3 | -2.0 |
| **DGo_PF0019** | | CDS |  | Ankyrin repeat protein, putative | -2.0 |
| **DGo_PC0194** | | CDS | *noxC* | Uncharacterized oxidoreductase czcO-like | -2.0 |
| **DGo_PB0309** | | CDS |  | Hypothetical protein | -2.0 |
