# Supplementary material for: Genome Sequence and Transcriptome Analysis of the Radioresistant Bacterium Deinococcus gobiensis: Insights into the Extreme Environmental Adaptations
Source: PLoS One. 2012 Mar 28;7(3):e34458. doi: 10.1371/journal.pone.0034458 (PMC3314630; doi:10.1371/journal.pone.0034458)
Supplement: Table S6 — Genomic islands in D. gobiensis. (DOC) [file pone.0034458.s008.doc]

**Table S6. Genomic islands in *D. gobiensis***

| **Islands** | **Start** | **Ends** | **G+C** |
| --- | --- | --- | --- |
| GI01 | DGo_CA0924 | DGo_CA0942 | 74.7% |
| GI02 | DGo_CA1052 | DGo_CA1071 | 74.2% |
| GI03 | DGo_CA1339 | DGo_CA1346 | 69.3% |
| GI04 | DGo_CA1416 | DGo_CA1422 | 72.5% |
| GI05 | DGo_CA1495 | DGo_CA1513 | 71.5% |
| GI06 | DGo_CA1551 | DGo_CA1557 | 71.0% |
| GI07 | DGo_CA1582 | DGo_CA1593 | 72.3% |
| GI08 | DGo_CA1792 | DGo_CA1807 | 72.2% |
| GI09 | DGo_CA1874 | DGo_CA1918 | 66.0% |
| GI10 | DGo_CA1943 | DGo_CA2005 | 65.3% |
| GI11 | DGo_CA2018 | DGo_CA2023 | 73.7% |
| GI12 | DGo_CA2286 | DGo_CA2295 | 74.5% |
| GI13 | DGo_CA2573 | DGo_CA2595 | 65.7% |
| GI14 | DGo_CA2706 | DGo_CA2730 | 71.7% |
| GI15 | DGo_CA2748 | DGo_CA2789 | 68.5% |
| GI16 | DGo_CA2865 | DGo_CA2878 | 68.6% |
| GI17 | DGo_CA2930 | DGo_CA2941 | 67.7% |
| GI18 | DGo_CA0140 | DGo_CA0160 | 72.3% |
| GI19 | DGo_CA0232 | DGo_CA0240 | 63.0% |
| GI20 | DGo_CA0367 | DGo_CA0384 | 68.8% |
| GI21 | DGo_CA0477 | DGo_CA0500 | 70.0% |
| GI22 | DGo_CA0658 | DGo_CA0770 | 69.9% |
| GI23 | DGo_PA0240 | DGo_PA0259 | 48.7% |
