# Supplementary material for: Genome Sequence and Transcriptome Analysis of the Radioresistant Bacterium Deinococcus gobiensis: Insights into the Extreme Environmental Adaptations
Source: PLoS One. 2012 Mar 28;7(3):e34458. doi: 10.1371/journal.pone.0034458 (PMC3314630; doi:10.1371/journal.pone.0034458)
Supplement: Table S7 — DNA repair genes (A), stress response-related genes (B) and additional enzymes of possible biotechnological interest genes (C) identified in sequenced deinococci. (DOC) [file pone.0034458.s009.doc]

**Table S7 DNA repair genes (A), stress response-related genes (B) and additional enzymes of possible biotechnological interest genes (C) in four deinococci**

**A. DNA repair genes in four deinococci**

| **1. Main DNA repair genes for Base Excision Repair (BER)** | | | | | | | | | | | | | | | | | | | | | | | | |
| --- | --- | --- | --- | --- | --- | --- | --- | --- | --- | --- | --- | --- | --- | --- | --- | --- | --- | --- | --- | --- | --- | --- | --- | --- |
| **1.1. Monofunctional DNA glycosylases** | | | | | | | | | | | | | | | | | | | | | | | | |
| **Gene name** | | | **Locus_tag** | | | **Product description** | | | | **Log_2_Fold** | | **Counterpart in the other three *Deioncoccus*** | | | | | | | | | | | | |
|  |  |  |  |  |  |  |  |  |  |  |  | ***D. radiodurans*** | | | | | ***D. deserti*** | ***D. geothermailis*** | | | | | | |
| *alkA* | | | DGo_CA2036 | | | 3-methyladenine-DNA glycosylaseII | | | | -2.43 | | DR_2074 | | | | | Deide_08050 | Dgeo_1660 | | | | | | |
|  |  |  | DGo_CA2905 | | | 3-methyladenine-DNA glycosylaseII | | | | -0.41 | | DR_2584 | | | | | Deide_02320 | Dgeo_0107 | | | | | | |
| *mutY* | | | DGo_CA2916 | | | A/8oxoG adenine glycosylase | | | | -0.52 | | DR_2285 | | | | | Deide_01970 | Dgeo_0019 | | | | | | |
| *mug* | | | DGo_CA1116 | | | Removes uracil,thymine or ethenocytosine opposite guanine | | | | 0.69 | | DR_0715 | | | | | Deide_17530 | Dgeo_1718 | | | | | | |
| *ung* | | | DGo_CA0399 | | | Uracil-DNA glycosylase | | | | 0.01 | | DR_0689 | | | | | Deide_00830 | Dgeo_2059 | | | | | | |
|  |  |  | DGo_CA1359 | | |  |  |  |  | -0.83 | | DR_1751 | | | | | - | Dgeo_1556 | | | | | | |
| **1.2. Bifunctional DNA glycosylases (displaying also a AP lyase activity)** | | | | | | | | | | | | | | | | | | | | | | | | |
| **Gene name** | | | **Locus_tag** | | | **Product description** | | | | **Log_2_Fold** | | **Counterpart in the other three *Deioncoccus*** | | | | | | | | | | | | |
|  |  |  |  |  |  |  |  |  |  |  |  | ***D. radiodurans*** | | | | | ***D. deserti*** | | | | | | ***D. geothermailis*** | |
| *mutM* | | | DGo_CA0896 | | | Formamidopyrimidine-DNA glycosylase | | | | -0.09 | | DR_0493 | | | | | Deide_16240 | | | | | | Dgeo_0442 | |
| *nth* | | | - | | | Endonuclease III; removes ring-saturated or fragmented pyrimidines | | | | - | | DR_0289 | | | | | Deide_01790 | | | | | | Dgeo_0248 | |
|  |  |  | DGo_CA0048 | | |  |  |  |  | 0.97 | | DR_2438 | | | | | Deide_23070 | | | | | | Dgeo_2290 | |
|  |  |  | DGo_CA0132 | | |  |  |  |  | -0.18 | | DR_2394 | | | | | Deide_22780 | | | | | | Dgeo_0254 | |
| **1.3. AP endonucleases** | | | | | | | | | | | | | | | | | | | | | | | | |
| **Gene name** | | | **Locus_tag** | | | **Product description** | | | | **Log_2_Fold** | | **Counterpart in the other three *Deioncoccus*** | | | | | | | | | | | | |
|  |  |  |  |  |  |  |  |  |  |  |  | ***D. radiodurans*** | | | ***D. deserti*** | | | | | | | | ***D. geothermailis*** | |
| *nfi* | | | DGo_CA2027 | | | Endonuclease V | | | | -0.29 | | DR_2162 | | | - | | | | | | | | - | |
| *xthA* | | | DGo_CA0439 | | | Exodeoxyribonulease III | | | | -0.38 | | DR_0354 | | | Deide_03250 | | | | | | | | Dgeo_0461 | |
|  |  |  |  |  |  |  |  |  |  |  |  | - | | | Deide_2p01470 | | | | | | | | Dgeo_2484 | |
| **2. Main DNA repair genes for Nucleotide Excision Repair (NER)** | | | | | | | | | | | | | | | | | | | | | | | | |
| **Gene name** | | | **Locus_tag** | | | **Product description** | | | | **Log_2_Fold** | | **Counterpart in the other three *Deioncoccus*** | | | | | | | | | | | | |
|  |  |  |  |  |  |  |  |  |  |  |  | ***D. radiodurans*** | | | ***D. deserti*** | | | | | | | ***D. geothermailis*** | | |
| *mfd* | | | DGo_CA0976 | | | transcription-repair coupling factor; helicase | | | | 0.70 | | DR_1532 | | | Deide_06920 | | | | | | | Dgeo_0545 | | |
| *uvrA* | | | DGo_CA1010 | | | DNA damage recognition protein UvrA; DNA independent ATPase and DNA binding protein | | | | 0.36 | | DR_1771 | | | Deide_12760 | | | | | | | Dgeo_0694 | | |
|  |  |  | DGo_CA0556 | | |  |  |  |  | -0.14 | | DR_A0188 | | | Deide_2p02060 | | | | | | | - | | |
| *uvrB* | | | DGo_CA0847 | | | DNA damage binding protein UvrB ; helicase | | | | 0.44 | | DR_2275 | | | Deide_03120 | | | | | | | Dgeo_1890 | | |
| *uvrC* | | | DGo_CA1577 | | | Excision nuclease | | | | -0.78 | | DR_1354 | | | Deide_11450 | | | | | | | Dgeo_1124 | | |
| *uvrD* | | | DGo_CA1449 | | | DNA helicase II | | | | 0.39 | | DR_1775 | | | Deide_12100 | | | | | | | Dgeo_0868 | | |
| *ywjD* | | | DGo_CA0536 | | | UV DNA damage endonuclease UvsE | | | | -0.05 | | DR_1819 | | | Deide_17800 | | | | | | | Dgeo_1819 | | |
| *yejH* | | | DGo_PA0135 | | | DNA or RNA helicase of superfamily II (COG1061); also predicted nuclease | | | | -0.85 | | DR_A0131 | | | Deide_17320 | | | | | | | - | | |
| **3. Main DNA Repair genes for Mismatch Repair** | | | | | | | | | | | | | | | | | | | | | | | | |
| **Gene name** | | | **Locus_tag** | | | **Product description** | | | | **Log_2_Fold** | | **Counterpart in the other three *Deioncoccus*** | | | | | | | | | | | | |
|  |  |  |  |  |  |  |  |  |  |  |  | ***D. radiodurans*** | | ***D. deserti*** | | | | | | | ***D. geothermailis*** | | | |
| *mutL* | | | DGo_CA0507 | | | DNA mismatch repair protein; ATPase | | | | 0.44 | | DR_1696 | | Deide_15600 | | | | | | | Dgeo_1538 | | | |
| *mutS1* | | | DGo_CA2405 | | | DNA mismatch repair protein; ATPase | | | | 0.05 | | NoAnnotation_610+DR_1039+NoAnnotation_671 | | Deide_15540 | | | | | | | Dgeo_1537 | | | |
| *mutS2* | | | DGo_CA2335 | | | DNA mismatch repair protein; ATPase | | | | 0.32 | | DR_1976 | | Deide_05000 | | | | | | | Dgeo_0899 | | | |
| *xseA* | | | DGo_CA0092 | | | Exonuclease VII, large subunit | | | | -0.85 | | DR_0186 | | Deide_22980 | | | | | | | Dgeo_0148 | | | |
| *xseB* | | | DGo_CA2907 | | | Exonuclease VII, small subunit | | | | -2.41 | | DR_2586 | | Deide_02120 | | | | | | | Dgeo_0027 | | | |
| **4. Main DNA repair genes for Direct Reversal of DNA damage (DR)** | | | | | | | | | | | | | | | | | | | | | | | | |
| **Gene name** | | | **Locus_tag** | | | **Product description** | | | **Log_2_Fold** | | | **Counterpart in the other three *Deioncoccus*** | | | | | | | | | | | | |
|  |  |  |  |  |  |  |  |  |  |  |  | ***D. radiodurans*** | | ***D. deserti*** | | | | | | | ***D. geothermailis*** | | | |
| *ogt/*  *ybaZ* | | | DGo_CA2747 | | | O-6-alkylguanine transferase | | | -0.10 | | | DR_0428 | | Deide_22770 | | | | | | | Dgeo_2101 | | | |
| *phrA* | | | DGo_CA1802 | | | photorepair protein PhrA | | | -0.53 | | | - | | - | | | | | | | - | | | |
| *phrB* | | | DGo_CA0607 | | | DNA photolyase | | | 1.66 | | | - | | - | | | | | | | - | | | |
| *splB* | | | DGo_PA0134 | | | spore photoproduct lyase | | | -1.32 | | | - | | Deide_3p02150 | | | | | | | - | | | |
| *dcd* | | | DGo_CA2281 | | | Deoxycytidine triphosphate deaminase | | | -1.90 | | | - | | Deide_16640 | | | | | | | Dgeo_1880 | | | |
| *yggV* | | | DGo_CA2488 | | | Xanthosine triphosphate pyrophosphatase | | | -0.74 | | | DR_0179 | | Deide_19360 | | | | | | | Dgeo_2209 | | | |
| **5. Main DNA repair genes for recombinational repair (RER)** | | | | | | | | | | | | | | | | | | | | | | | | |
| **Gene name** | | | **Locus_tag** | | **Product description** | | | | **Log_2_Fold** | | | **Counterpart in the other three *Deioncoccus*** | | | | | | | | | | | | |
|  |  |  |  |  |  |  |  |  |  |  |  | ***D. radiodurans*** | | | ***D. deserti*** | | | | | ***D. geothermailis*** | | | | |
| *recA* | | | DGo_CA2833 | | DNA strand exchange and renaturation, DNA-dependent ATPase, DNA-and ATP-dependent coprotease | | | | -0.44 | | | DR_2340 | | | Deide_19450, Deide_1p01260,Deide_3p00210 | | | | | Dgeo_2138 | | | | |
| *recB* | | | DGo_PB0022,  DGo_PC0098 | | DNA helicase, ATP-dependent dsDNA/ssDNA exonuclease V subunit, ss DNA endonuclease | | | | 1.58 | | | - | | | - | | | | | - | | | | |
| *recD* | | | DGo_CA1825 | | DNA helicase, ATP-dependent dsDNA/ssDNA exonuclease V subunit, ss DNA endonuclease | | | | -0.67 | | | DR_1902 | | | Deide_16210 | | | | | Dgeo_0826 | | | | |
| *recD2* | | | DGo_PB0015,  DGo_PC0277 | | Superfamily I DNA and RNA helicases | | | |  | | | - | | | - | | | | | - | | | | |
| *recF* | | | DGo_CA2190 | | Recombinational repair protein | | | | -0.72 | | | DR_1089 | | | Deide_14250 | | | | | Dgeo_1620 | | | | |
| *recG* | | | DGo_CA1320 | | Holliday junction-specific DNA helicase; branch migration inducer | | | | -0.04 | | | DR_1916 | | | Deide_09960 | | | | | Dgeo_1139 | | | | |
|  |  |  | DGo_CA0381 | | Holliday junction-specific DNA helicase; branch migration inducer | | | | 2.58 | | | - | | | - | | | | | - | | | | |
| *recJ* | | | DGo_CA0910 | | ssDNA exonuclease, 5’->3’ specific | | | | -0.01 | | | DR_1126+NoAnnotation_611 | | | Deide_07130 | | | | | Dgeo_1599 | | | | |
| *recN* | | | DGo_CA1486 | | Recombination and repair protein | | | | -0.89 | | | DR_1477 | | | Deide_12310 | | | | | Dgeo_1194 | | | | |
| *recO* | | | DGo_CA2367 | | Bacterial recombinational repair protein | | | | -0.43 | | | DR_0819 | | | Deide_13810 | | | | | Dgeo_0855 | | | | |
| *recQ* | | | DGo_CA1784,DGo_PB0141 | | ATP-dependent DNA helicase RecQ | | | | US | | | DR_1289 | | | Deide_11320 | | | | | NoAnnotation_505+Dgeo_1226 | | | | |
|  |  |  | DGo_CA2913 | | ATP-dependent DNA helicase RecQ | | | | -0.77 | | | DR_2444 | | | Deide_02180 | | | | | Dgeo_0021 | | | | |
| *recR* | | | DGo_CA2078 | | Recombination and repair protein | | | | -0.22 | | | DR_0198 | | | Deide_06340 | | | | | Dgeo_1513 | | | | |
| *recT* | | | - | | Recombinase, DNA renaturation | | | |  | | | - | | | - | | | | | - | | | | |
| *recX* | | | DGo_CA2088 | | Regulatory protein, RecX | | | | -1.97 | | | DR_1310 | | | Deide_12350 | | | | | Dgeo_1433 | | | | |
| *radA* | | | DGo_CA1836 | | DNA repair protein RadA | | | | -0.12 | | | DR_1105 | | | Deide_12660 | | | | | Dgeo_1212 | | | | |
| *ruvA* | | | DGo_CA1508 | | Holliday junction helicase subunit A; branch migration | | | | -0.61 | | | DR_1274 | | | Deide_09360 | | | | | Dgeo_0726 | | | | |
| *ruvB* | | | DGo_CA2516 | | Holliday junction helicase subunit A; branch migration | | | | -0.26 | | | DR_0596 | | | Deide_18350 | | | | | Dgeo_0404 | | | | |
| *ruvC* | | | DGo_CA0294 | | Holliday junction endonuclease | | | | -0.94 | | | DR_0440 | | | Deide_20630 | | | | | Dgeo_0327 | | | | |
| *yqgF* | | | DGo_CA0376 | | Putative Holliday junction resolvase | | | | 1.42 | | | DR_2509 | | | Deide_04280 | | | | | Dgeo_0425 | | | | |
| *sbcC* | | | DGo_CA1827 | | ATP dependent dsDNA exonuclease | | | | -1.40 | | | DR_1922 | | | Deide_16170 | | | | | Dgeo_0823 | | | | |
| *sbcD* | | | DGo_CA1826 | | ATP dependent dsDNA exonuclease | | | | -0.40 | | | DR_1921 | | | Deide_16180 | | | | | Dgeo_0824 | | | | |
| *ssb* | | | DGo_CA0126 | | Single-stranded DNA-binding protein | | | | 0.10 | | | NoAnnotation_542+DR_0099 | | | Deide_00120 | | | | | Dgeo_0165,Dgeo_2964 | | | | |
| **6. Other DNA repair related genes** | | | | | | | | | | | | | | | | | | | | | | | | |
| **Gene name** | | **Locus_tag** | | | **Product description** | | | **Log_2_Fold** | | | | **Counterpart in the other three *Deioncoccus*** | | | | | | | | | | | | |
|  |  |  |  |  |  |  |  |  |  |  |  | ***D. radiodurans*** | ***D. deserti*** | | | | | | ***D. geothermailis*** | | | | | |
| *dnlJ* | | DGo_CA1174 | | | DNA ligase, NAD-dependent | | | -0.07 | | | | DR_2069 | Deide_12290 | | | | | | Dgeo_0696 | | | | | |
| *gyrA* | | DGo_CA1041 | | | DNA gyrase subunit A | | | 0.99 | | | | DR_1913 | Deide_12520 | | | | | | Dgeo_1016 | | | | | |
| *gyrB* | | DGo_CA0873 | | | DNA gyrase subunit B | | | 1.03 | | | | DR_0906 | Deide_15490 | | | | | | Dgeo_0546 | | | | | |
| *topA* | | DGo_CA0471 | | | DNA topoisomerase I | | | -0.28 | | | | DR_1374 | Deide_07410 | | | | | | Dgeo_2001 | | | | | |
|  |  | DGo_CA0400 | | | DNA topoisomerase I | | | -0.56 | | | | DR_0690 | Deide_00840 | | | | | | Dgeo_2058 | | | | | |
| *lexA* | | DGo_PC0001 | | | Transcriptional regulator, repressor of the SOS regulon, autoprotease | | | 0.94 | | | | DR_A0344 | Deide_1p01870 | | | | | | - | | | | | |
|  |  |  | | |  |  |  |  | | | | - | Deide_01180 | | | | | | Dgeo_1366 | | | | | |
| *polA* | | DGo_CA1437 | | | DNA polymerase I | | | 0.01 | | | | DR_1707 | Deide_15130 | | | | | | Dgeo_1666 | | | | | |
| *polB* | | DGo_PC0151 | | | DNA polymerase II | | | 1.00 | | | | - | Deide_1p00180 | | | | | | - | | | | | |
| *dnaE* | | DGo_CA2511 | | | DNA polymerase III alpha subunit | | | -0.32 | | | | DR_0507 | Deide_21950 | | | | | | Dgeo_0255 | | | | | |
| *dnaQ* | | DGo_CA0849 | | | DNA polymerase III epsilon subunit (3'-5' exonuclease subunit) | | | 0.76 | | | | DR_0856 | Deide_17790 | | | | | | Dgeo_1818 | | | | | |
| *dnaN* | | DGo_CA0002 | | | DNA polymerase III beta subunit | | | 1.24 | | | | DR_0001 | Deide_00020 | | | | | | Dgeo_0003 | | | | | |
| *dnaX* | | DGo_CA2827 | | | DNA polymerase III tau/gamma subunit | | | -0.70 | | | | DR_2410+DR_2411 | Deide_01610 | | | | | | Dgeo_2135 | | | | | |
| *holA* | | DGo_CA1455 | | | DNA polymerase III delta subunit | | | -0.67 | | | | DR_1244 | Deide_10170 | | | | | | Dgeo_0745 | | | | | |
| *dinB* | | DGo_CA2228 | | | Pol IV, DNA polymerase type-Y family, contains 1 UmuC domain Related to putative Y-family DNA polymerase | | | 0.23 | | | | - | - | | | | | | - | | | | | |
| *holB* | | DGo_CA0068 | | | DNA polymerase III delta prime subunit | | | -0.20 | | | | DR_2332 | Deide_21710 | | | | | | Dgeo_2262 | | | | | |
| *yshC* | | DGo_CA0274 | | | DNA polymerase, family X | | | -0.35 | | | | DR_0467 | Deide_07030 | | | | | | Dgeo_1609 | | | | | |
| *-* | | DGo_CA2206 | | | UvrD/REP helicase; COG0210, UvrD, Superfamily I DNA and RNA helicases | | | 0.37 | | | | - | Deide_06250 | | | | | | Dgeo_0552 | | | | | |
| *-* | | DGo_PB0015, DGo_PC0025, DGo_PC0277, DGo_PD0049 | | | UvrD/REP helicase | | |  | | | |  |  | | | | | |  | | | | | |
| *ywqA* | | DGo_CA1433 | | | DNA helicase SNF2/Rad54 family | | | 0.92 | | | | DR_1258+DR_1259 | Deide_08980 | | | | | | Dgeo_1491 | | | | | |
| *dnaA* | | DGo_CA0001 | | | Chromosomal replication initiator protein | | | -0.73 | | | | DR_0002 | Deide_00010 | | | | | | Dgeo_0001 | | | | | |
| *dnaB* | | DGo_CA2884 | | | Replicative DNA helicase | | | 0.39 | | | | DR_0549 | Deide_04710 | | | | | | Dgeo_2037 | | | | | |
| *dnaG* | | DGo_CA0378 | | | DNA primase | | | 0.57 | | | | DR_0601 | Deide_04900 | | | | | | Dgeo_1910 | | | | | |
| *priA* | | DGo_CA2791 | | | Primosomal protein N' | | | -0.11 | | | | DR_2606 | Deide_00480 | | | | | | Dgeo_0271 | | | | | |
| *rarA* | | DGo_CA2103 | | | Replication-associated recombination protein | | | -1.02 | | | | DR_1898 | Deide_04980 | | | | | | Dgeo_1401 | | | | | |
| **7. Other radiation tolerance-associated genes in Locus_tag and other *Deinococcus* spp.** | | | | | | | | | | | | | | | | | | | | | | | | |
| **Gene name** | **Locus_tag** | | | **Product description** | | | **Log2Fold** | | | | **Counterpart in the other three *Deioncoccus*** | | | | | | | | | | | | | |
|  |  |  |  |  |  |  |  |  |  |  | ***D. radiodurans*** | | | | | ***D. deserti*** | | | | | | | | ***D. geo-thermailis*** |
| *ddrA* | DGo_CA2046 | | | DNA damage response protein DdrA; Rad52/22 double-strand break repair protein | | | 0.96 | | | | DR_0423 | | | | | Deide_09150 | | | | | | | | Dgeo_0977 |
| *ddrB* | DGo_CA0350 | | | DdrB, radiation induced single-stranded DNA binding protein | | | 1.12 | | | | DR_0070 | | | | | Deide_02990 | | | | | | | | Dgeo_0295 |
| *ddrC* | DGo_CA0071 | | | DdrC | | | 1.09 | | | | DR_0003 | | | | | NoAnnotation_250 | | | | | | | | Dgeo_0047 |
| *ddrD* | DGo_CA2608 | | | DdrD | | | 0.43 | | | | DR_0326 | | | | | Deide_01160 | | | | | | | | Dgeo_2186 |
| *ddrE* | DGo_CA0988 | | | DdrE; Related to zinc metal lopeptidase | | | 0.96 | | | | DR_0194 | | | | | Deide_11220 | | | | | | | | Dgeo_1282 |
| *ddrH* | DGo_CA0299 | | | DdrH | | | 0.16 | | | | DR_0438 | | | | | NoAnnotation_335 | | | | | | | | NoAnnotation_431 |
| *ddrI* | DGo_CA1040 | | | DdrI; Transcriptional regulator, Crp/Fnr family | | | 1.82 | | | | DR_0997 | | | | | Deide_12530 | | | | | | | | Dgeo_1015 |
| *ddrN* | DGo_CA0142 | | | DdrN | | | -0.17 | | | | DR_2441 | | | | | Deide_02930 | | | | | | | | Dgeo_0078 |
| *ddrO* | DGo_CA0310 | | | DdrO, transcriptional regulator, HTH_3 family | | | 0.19 | | | | DR_2574 | | | | | Deide_20570,Deide_3p02170 | | | | | | | | Dgeo_0336 |
| *ddrP* | DGo_CA2239 | | | DdrP | | | 0.93 | | | | DR_B0100 | | | | | - | | | | | | | | - |
| *frnE* | DGo_CA0380 | | | predicted dithiol-disulfide isomerase | | |  | | | | DR_0659 | | | | | Deide_00690 | | | | | | | | Dgeo_2073 |
| *hicB* | - | | | HicB | | |  | | | | DR_B0141 | | | | | - | | | | | | | | - |
| *irrE* | DGo_CA2805 | | | IrrE, regulator of recA expression in D. radiodurans | | | 0.05 | | | | DR_0167 | | | | | Deide_03030 | | | | | | | | Dgeo_0395 |
| *irrI* | - | | | IrrI | | |  | | | | DR_0171 | | | | | - | | | | | | | | - |
| *pprA* | DGo_PA0354 | | | DNA damage repair protein PprA | | | 0.11 | | | | DR_A0346 | | | | | Deide_2p01380 | | | | | | | | Dgeo_2628 |
| *pprM* | DGo_CA1136 | | | PprM | | | 0.66 | | | | DR_0907 | | | | | Deide_2p00490 | | | | | | | | Dgeo_0638 |
|  | DGo_PA0041 | | |  |  |  | 0.20 | | | | - | | | | | Deide_3p00840 | | | | | | | | - |

**B. Stress response-related genes in four deinococci.**

| **Stress** | **Gene name** | **Locus_tag** | **Product description** | **Log_2_Folder)** | **Counterpart in the other three *Deioncoccus*** | | | |
| --- | --- | --- | --- | --- | --- | --- | --- | --- |
|  |  |  |  |  | ***D. deserti*** | ***D. radiodurans*** | ***D. geothermailis*** |  |
| **Desiccation** | *-* | DGo_CA1605 | Desiccation-related protein, LEA 14 family | 0.06 | Deide_09710 | DR_1372 | Dgeo_1551 |  |
|  | *-* | DGo_CA1631 | LEA76 family desiccation resistance protein | 0.46 | Deide_08510 | DR_1172 | Dgeo_1473 |  |
|  | *-* | DGo_CA1742 | Desiccation-related protein | 0.68 | Deide_08080 | - | Dgeo_1323 |  |
|  | *-* | DGo_CA2349 | Desiccation-related protein | 0.06 | Deide_07540 | DR_B0118 | Dgeo_0097 |  |
| **General&Hot** | *ytxJ* | DGo_CA1021 | Thioredoxin-like | 1.74 | Deide_14700 | DR_1832 | Dgeo_1464 |  |
|  | *clpA* | DGo_CA1279 | ATPase subunits of Clp protease | -0.74 | Deide_08490 | DR_0588 | Dgeo_1475 |  |
|  | *clpB* | DGo_CA1752 | ATPase subunits of Clp protease | 0.14 | Deide_12640 | DR_1046 | Dgeo_1210 |  |
|  | *clpC* | DGo_CA1460 | ATPase subunits of Clp protease | -0.34 | Deide_12680 | DR_1117 | Dgeo_1215 |  |
|  | *clpP* | DGo_CA0282 | ATP-dependent protease with chaperone activity | -0.66 | Deide_07080 | DR_0078 | Dgeo_1604 |  |
|  | *clpQ* | - | ATP-dependent protease HslV | - | - | - | - |  |
|  | *clpX* | DGo_CA2328 | ATPase subunit of Clp protease | 0.02 | Deide_19580 | DR_1973 | Dgeo_2152 |  |
|  | *-* | DGo_CA0852 | GTPase, protease modulator | 0.81 | Deide_18500 | DR_0646 | Dgeo_1825 |  |
|  | *dksA* | - | DnaK suppressor protein | - | - | - | - |  |
|  | *dnaJ* | DGo_CA0461 | Hsp70, chaperone cofactor | 0.45 | Deide_07590 | DR_1424 | Dgeo_0451 |  |
|  |  | DGo_CA1008 |  | -0.92 | Deide_12740 | DR_0753 | Dgeo_0692 |  |
|  | *dnaK* | DGo_CA2644 | Hsp70, molecular chaperone | 0.04 | Deide_21970 | DR_0129 | Dgeo_2076 |  |
|  | *ftsH* | - | ATP-dependent Zn protease | - | Deide_01120 | DR_A0290 | Dgeo_2182 |  |
|  |  | DGo_CA2394 |  | 0.18 | Deide_18550 | DR_1020 | Dgeo_1832 |  |
|  |  | DGo_CA2646 |  | -0.24 | Deide_23310 | DR_0583 | Dgeo_2075 |  |
|  | *groL* | DGo_CA2525 | Hsp10, molecular chaperone | 0.20 | Deide_22590 | DR_0607 | Dgeo_2231 |  |
|  | *groS* | DGo_CA2524 | Hsp60, molecular chaperone | -0.17 | Deide_22580 | DR_0606 | Dgeo_2230 |  |
|  | *grpE* | DGo_CA2643 | Hsp20, molecular chaperone | -0.26 | Deide_21980 | DR_0128 | Dgeo_2077 |  |
|  | *hflX* | DGo_CA2820 | GTPase, protease modulator | 0.73 | Deide_19040 | DR_0139 | Dgeo_2006 |  |
|  | *hit* | DGo_CA0393 | Diadenosine tetraphosphate (Ap4A) hydrolase, HIT family, cell cycle regulation | -1.23 | Deide_12650 | - | Dgeo_2064 |  |
|  |  | DGo_CA1744 |  | 0.02 | Deide_15190 | DR_1621 | Dgeo_1329 |  |
|  | *hslJ* | DGo_CA0947 | Related to heat shock protein | 0.54 | Deide_07610 | DR_1940 | - |  |
|  | *htpG* | - | Hsp90, molecular chaperone | - | - | - | - |  |
|  | *htpX* | DGo_CA0938 | Predicted Zn-dependent proteases (possible chaperone) | -0.34 | Deide_21060 | - | - |  |
|  |  | DGo_CA0988 |  | 0.96 | Deide_11220 | DR_0194 | Dgeo_1282 |  |
|  | *htrA* | DGo_CA0185 | Do serine protease, with PDZ domain | 0.43 | Deide_21550 | DR_0300+NoAnnotation_835 | Dgeo_0103 |  |
|  |  | DGo_CA1376 |  | 0.24 | Deide_10420 | DR_1756 | Dgeo_0676 |  |
|  |  | DGo_CA1644 |  | 0.83 | Deide_12870 | DR_1703 | Dgeo_0795 |  |
|  |  | DGo_CA1790 |  | -0.02 | Deide_07680 | DR_2154 | Dgeo_1060 |  |
|  |  | DGo_CA1941 |  | -0.41 | - | DR_1599+DR_1600 | - |  |
|  |  | DGo_CA2420 |  | -1.32 | Deide_04820 | DR_0745 | Dgeo_2025 |  |
|  | *ibpA/ibpB* | DGo_CA1726 | Small heat shock protein | 1.06 | Deide_11290 | DR_1691 | Dgeo_0859 |  |
|  |  | DGo_CA2067 |  | 0.40 | Deide_18090 | DR_1114 | Dgeo_0505 |  |
|  | *lon* | DGo_CA1831 | ATP-dependent Lon Serine protease | -0.58 | - | DR_0394 | Dgeo_2485 |  |
|  | ***lo****nA* | DGo_CA2327 | ATP-dependent Lon Serine protease | -0.08 | Deide_19590 | DR_1974 | Dgeo_2153 |  |
|  | *prc* | DGo_CA1196 | Tail-specific periplasmic serine protease | -0.30 | Deide_10700 | DR_1551 | Dgeo_1479 |  |
|  |  | DGo_CA1459 |  | -0.11 | Deide_12780 | DR_1491 | Dgeo_1216 |  |
|  |  | DGo_CA2561 |  | 0.68 | Deide_21230 | DR_1308 | Dgeo_0277 |  |
|  | *radA* | DGo_CA1836 | ATP-dependent serine protease COG1066 / DNA repair protein | -0.12 | Deide_12660 | DR_1105 | Dgeo_1212 |  |
|  | *sugE* | DGo_CA2538 | Small multidrug resistance membrane protein | 0.56 | - | - | Dgeo_1956 |  |
|  |  | DGo_CA2539 |  | -0.08 | - | - | Dgeo_1957 |  |
|  |  | DGo_CA2790 |  | 0.14 | Deide_20500 | DR_1004,DR_1005 | Dgeo_2170 |  |
|  | *thiJ* | DGo_CA0475 | Protease I, related to general stress protein 18, ThiJ superfamily protein | 0.65 | Deide_04910 | DR_1199 | Dgeo_0863 |  |
|  | *uspA* | - | Universal stress protein, nucleotide-binding | - | Deide_08840 | DR_2132 | Dgeo_1279 |  |
|  |  | DGo_CA0010 |  | -0.11 | Deide_22300 | DR_2363 | Dgeo_0155 |  |
|  | *yaeL* | DGo_CA1846 | Membrane-associated Zn-dependent protease I | -0.77 | Deide_13240 | DR_1507 | Dgeo_1043 |  |
|  | *yebL* | - | Zn-binding (lipo)protein of the ABC type Zn transport system (surface adhesin A) | - | Deide_2p01321,Deide_3p02720 | - | - |  |
|  |  | DGo_CA2604 |  | -0.80 | Deide_07330 | DR_2523+NoAnnotation_871 | Dgeo_0534 |  |
| **Osmotic** | *aqpZ* | - | Major intrinsic protein (aquaporin Z and glycerol uptake facilitator) | - | Deide_1p01582,Deide_3p02450 | - | Dgeo_0516 |  |
|  |  | DGo_CA0704 |  | -1.20 | - | - | Dgeo_1799 |  |
|  | *glpF* | DGo_CA2316 | Major intrinsic protein (aquaporin Z and glycerol uptake facilitator) | -0.51 | Deide_2p00230 | DR_1929 | Dgeo_2523 |  |
|  | *kdpD* | DGo_CA0646 | Osmosensitive K+ channel histidine kinase sensor domain | -1.61 | - | DR_B0088 | Dgeo_2852 |  |
|  | *mscL* | DGo_CA2713 | Large conductance mechano-sensitive channel | 0.45 | Deide_02220 | DR_2422 | Dgeo_0305 |  |
|  | *otsA* | - | Trehalose-6-phosphatase synthase | - | Deide_21370 | - | Dgeo_0059 |  |
|  | *otsB* | - | Trehalose-6-phosphatase  Maltooligosyltrehalose trehalohydrolase TreZ | - | Deide_21360 | - | Dgeo_0060 |  |
|  |  | DGo_CA2461 |  | 0.22 | Deide_2p01290 | DR_0464 | Dgeo_0540 |  |
|  |  | DGo_CA2462 |  | -0.61 | Deide_16570 | DR_0463 | Dgeo_0539 |  |
|  |  | DGo_CA2600 |  | -0.35 | Deide_07350 | DR_2036 | Dgeo_0537 |  |
|  | *proV* | - | Proline/glycine betaine ABC-type transport, ATPase subunit | - | Deide_00170 | DR_A0137 | Dgeo_0172 |  |
|  | *proW* | - | Proline/glycine betaine ABC-type transport, permease subunit | - | Deide_00180 | DR_A0136 | Dgeo_0173 |  |
|  |  | DGo_CA2785 |  | -0.83 | Deide_20480 | DR_0283 | Dgeo_2168 |  |
|  | *trkA* | - | Potassium uptake system, NAD-binding component | - | - | - | - |  |
|  | *trkH/trkG* | - | Potassium uptake component | - | Deide_3p00460 | DR_1668 | Dgeo_1583 |  |
|  |  | DGo_CA1233 |  | 1.83 | Deide_03830 | DR_1667 | - |  |
|  | *yehZ* | DGo_PA0197 | ABC-type proline/glycine/betaine transport system, periplasmic component |  | DR_A0135 | Deide_00190 | Dgeo_0174 |  |
|  | *yehY* | DGo_PA0198 | ABC-type proline/glycine/betaine transport system, permease component |  | DR_A0136 | Deide_00180 | Dgeo_0173 |  |
|  | *yehW2* | DGo_PA0199 | ABC-type proline/glycine/betaine transport system, ATPase component |  | DR_A0137 | Deide_00170 | Dgeo_0172 |  |
|  | *yehW* | DGo_PA0200 | ABC-type proline/glycine/betaine transport system, permease component |  | DR_A0138 | Deide_00160 | Dgeo_0171 |  |
|  | *yggB* | DGo_CA0865 | Membrane protein | -0.29 | Deide_18560 | DR_1995 | Dgeo_1833 |  |
| **Other** | *arsC* | - | Arsenate oxidoreductase (ArsC-like Rodanese protein) | - | Deide_3p02440 | - | Dgeo_2768 |  |
|  |  | - |  | - | - | DR_A0123 | Dgeo_0756,Dgeo_2548 |  |
|  |  | DGo_CA2817 |  | 0.25 | Deide_18400 | DR_0136 | Dgeo_0403 |  |
|  | *BS_yloU/BS_yqhY* | DGo_CA1110 | Alkaline shock protein, function unknown | -0.16 | Deide_17570 | DR_0389 | Dgeo_1721 |  |
|  |  | DGo_CA1175 |  | -0.85 | Deide_12280 | DR_2068 | Dgeo_0697 |  |
|  | *pprM* | DGo_PA0041 | Cold shock protein, OB fold nucleic acid binding protein | - | Deide_09930 | - | Dgeo_1006 |  |
|  |  | DGo_CA1136 |  | 0.66 | Deide_2p00490,Deide_3p00840 | DR_0907 | Dgeo_0638 |  |
|  | *hupA* | DGo_CA0549 | Histone-like nucleoid DNA-binding protein | 0.03 | Deide_2p01940 | DR_A0065 | Dgeo_2501 |  |
|  |  | DGo_CA0787 |  | -1.07 | Deide_1p00200,Deide_2p00460 | - | - |  |
|  | *pspA* | DGo_CA1515 | Phage shock protein A, controls membrane integrity | -0.02 | Deide_09450 | DR_1473 | Dgeo_0996 |  |
| **Oxidative&detoxication** | *-* | - | Fe dependent peroxidase | - | - | - | - |  |
|  | *-* | - | Mn-containing catalase | - | - | - | - |  |
|  | *cyp* | - | Cytochrome P450 | - | - | DR_C0001 | - |  |
|  |  | DGo_PB0312 |  | - | - | - | - |  |
|  |  | DGo_PB0521 |  | - | - | - | - |  |
|  |  | - |  | - | Deide_22920 | DR_2538 | - |  |
|  |  | DGo_CA0998 |  | 0.03 | Deide_01550 | DR_A0186+NoAnnotation_878 | Dgeo_0143 |  |
|  |  | DGo_CA1464 |  | -1.44 | - | DR_1723 | - |  |
|  |  | DGo_CA1830 |  | -2.03 | Deide_08170 | - | Dgeo_0944 |  |
|  |  | DGo_CA2870 |  | -0.02 | - | DR_2473 | - |  |
|  | *ahpC/ahpE-like* | DGo_CA2657 | Thioredoxin reductase/alkyl hydroperoxide reductase | 0.63 | Deide_02430 | DR_2242 | Dgeo_0122 |  |
|  | *ahpf/trxB* | - | Thiol-alkyl hydroperoxide reductase | - | Deide_3p01180 | DR_B0033 | - |  |
|  |  | DGo_CA0078 |  | -0.39 | Deide_23360 | DR_2623 | Dgeo_2331 |  |
|  |  | DGo_CA1038 |  | -0.11 | Deide_12541 | - | Dgeo_1013 |  |
|  |  | DGo_CA2053 |  | 0.63 | Deide_09090 | DR_0412 | Dgeo_0975 |  |
|  |  | DGo_CA2339 |  | -0.21 | Deide_05800 | DR_1982 | Dgeo_1576,Dgeo_2772 |  |
|  | *bcp* | DGo_CA1364 | Peroxiredoxin, bacterioferritin comigratory protein, antioxidant protein | 0.71 | Deide_10900 | DR_0846 | NoAnnotation_481 |  |
|  |  | DGo_CA1403 |  | 0.76 | Deide_09051 | DR_1209 | Dgeo_0990 |  |
|  | *BS_yceH* | DGo_CA1262 | Toxic anion resistance protein, possibly tellurite resistance | -0.52 | Deide_05270 | DR_1127 | Dgeo_0931 |  |
|  | *fur* | - | Ferric uptake regulation protein | - | Deide_2p00340 | - | Dgeo_2727 |  |
|  |  | DGo_CA0886 |  | 0.80 | Deide_16041 | DR_0865 | Dgeo_0519 |  |
|  |  | DGo_CA2836 |  | 0.06 | Deide_19480 | NoAnnotation_640+NoAnnotation_586 | Dgeo_2141 |  |
|  | *grxA* | - | Glutaredoxin | - | - | DR_A0072 | Dgeo_2583 |  |
|  |  | DGo_CA2073 |  | 0.61 | Deide_06390 | DR_2085 | Dgeo_1508 |  |
|  | ***katA*** | DGo_CA2912 | Catalase; Eukaryotic type | 0.25 | - | DR_A0146 | - |  |
|  | *katE* | DGo_CA1736 | Catalase | -0.15 | Deide_2p00330 | DR_1998 | Dgeo_2728 |  |
|  | *katG* | DGo_PA0127 | Catalase (peroxidase) | - | - | - | - |  |
|  | *katX* | DGo_PA0314 | Catalase X |  | DR_1998 | Deide_2p00330 | Dgeo_2728 |  |
|  | *msrA* | DGo_CA1541 | Peptide methionine sulfoxide reductase A | 0.47 | Deide_10980 | DR_1849 | Dgeo_0843 |  |
|  | *msrB* | DGo_CA0919 | Peptide methionine sulfoxide reductase B | 0.64 | Deide_04050 | DR_1378 | Dgeo_2072 |  |
|  | *nrdH* | DGo_CA0407 | Glutaredoxin | -2.21 | Deide_13741 | DR_0057 | Dgeo_0729 |  |
|  | *osmC* | DGo_CA0901 | Protein involved in alkylperoxide and oxidative stress response, osmotically induced protein | 0.53 | - | DR_1857 | Dgeo_0446 |  |
|  |  | DGo_CA1241 |  | 1.10 | Deide_16090 | DR_1538 | Dgeo_0526 |  |
|  | *oxyR* | - | Transcriptional regulator, LysR family | - | Deide_3p01240 | - | Dgeo_2711 |  |
|  |  | DGo_CA0172 |  | 0.00 | Deide_16400 | - | Dgeo_1692 |  |
|  |  | DGo_CA0539 |  | -1.12 | Deide_03130 | DR_0615 | Dgeo_1888 |  |
|  |  | DGo_CA1575 |  | -0.63 | - | - | Dgeo_2840 |  |
|  | sodA | DGo_CA2200 | Superoxide dismutase Mn or Fe dependent | 0.12 | Deide_07760 | DR_1279 | Dgeo_0830 |  |
|  | sodC | - | Superoxide dismutase Cu/Zn dependent | - | Deide_19880 | DR_1546 | - |  |
|  |  | DGo_CA1102 |  | 0.08 | Deide_1p00740 | DR_A0202 | - |  |
|  | soxR | - | Transcriptional regulator, MerR family | - | Deide_3p02530 | DR_2306 | Dgeo_2202 |  |
|  |  | - |  | - | Deide_04091 | DR_1111 | - |  |
|  |  | DGo_CA0538 |  | -0.06 | Deide_2p01800 | DR_2448 | Dgeo_1424 |  |
|  | *tlp* | DGo_CA2017 | Peroxiredoxin, disulfide reductase | 0.68 | Deide_08290 | DR_0189 | Dgeo_1248 |  |
|  | trx | - | Thioredoxin | - | Deide_01140 | DR_A0164 | Dgeo_2518 |  |
|  |  | DGo_CA0861 |  | 0.74 | Deide_18600 | DR_0944 | Dgeo_1837 |  |
|  | yhfA | DGo_CA1763 | Protein involved in alkylperoxide and oxidative stress response, osmotically induced protein | 1.06 | Deide_10790 | DR_1177 | Dgeo_1268 |  |
| Starvation | cstA | - | Carbon starvation-induced protein, membrane | - | - | - | - |  |
|  | dps | DGo_CA0262 | Starvation inducible DNA-binding protein | 0.55 | Deide_21200 | DR_2263 | Dgeo_0281 |  |
|  |  | DGo_CA0741 |  | -0.23 | - | DR_B0092 | - |  |
|  | mazE | - | Regulatory protein, MazF antagonist | - | - | - | - |  |
|  | mazF | - | ppGpp regulated growth inhibitor | - | - | DR_0417 | Dgeo_1937 |  |
|  | ppx | - | Phosphatase of ppGpp | - | Deide_01820 | DR_A0185 | - |  |
|  | spoT | DGo_CA1014 | Guanosine polyphosphate (ppGpp) pyrophosphohydrolase/synthetase | 0.25 | Deide_14760 | DR_1838 | Dgeo_1308 |  |

**Table S6C. Additional enzymes of possible biotechnological interest**

| Class / pathway | Locus tag | function |
| --- | --- | --- |
| DNA / RNA processing enzymes | DGo_CA1437 | DNA polymerase I |
|  | DGo_PC0151 | DNA polymerase II |
|  | DGo_CA0002, DGo_CA0068, DGo_CA0849, DGo_CA1455, DGo_CA2511, DGo_CA2827, DGo_PB0516 | DNA polymerase III |
|  | DGo_CA0274 | DNA polymerase X |
|  | DGo_PB0095 | DNA polymerase |
|  | DGo_CA2228 | Nucleotidyltransferase/DNA polymerase involved in DNA repair |
|  | DGo_CA1174, DGo_PB0078, DGo_PD0007 | DNA ligase |
|  | DGo_CA0381, DGo_CA1320, DGo_CA1433, DGo_CA1449, DGo_CA1508, DGo_CA1784, DGo_CA1885, DGo_CA2516, DGo_CA2884, DGo_PA0147, DGo_PB0141, DGo_PB0482, DGo_PC0263, DGo_PD0043, DGo_PF0017 | DNA helicase |
|  | DGo_CA0400, DGo_CA0471, DGo_CA0873, DGo_CA1041, DGo_CA2574, DGo_PB0093, DGo_PC0276 | DNA topoisomerase |
|  | DGo_CA0710, DGo_CA0792, DGo_CA1063, DGo_PB0079, DGo_PB0343, DGo_PC0210, DGo_PE0056 | DNA gyrase |
|  | DGo_CA0048, DGo_CA1807, DGo_CA2661 | endonuclease III |
|  | DGo_CA0165 | endonuclease IV |
|  | DGo_PA0054 | endonuclease V |
|  | DGo_CA2778, DGo_PC0006 | Restriction endonuclease S-submit |
|  | DGo_CA0536 | UV endonuclease |
|  | DGo_CA0294 | Crossover junction endodeoxyribonuclease ruvC |
|  | DGo_PD0033 | mismatch endonuclease |
|  | DGo_CA2841, DGo_PB0373, DGo_PB0497, DGo_PE0071 | HNH endonuclease |
|  | DGo_CA0439 | Exodeoxyribonuclease III |
|  | DGo_CA0092, DGo_CA0092 | Exodeoxyribonuclease VII |
|  | DGo_PC0008, DGo_CA2781 | Type I restriction-modification deoxyribonuclease |
|  | DGo_CA2027 | Deoxyinosine 3'endonuclease |
|  | DGo_CA0024 | Ribonuclease II |
|  | DGo_CA2497 | Ribonuclease BN |
|  | DGo_CA0972, DGo_CA2741 | Ribonuclease H |
|  | DGo_CAnc18 | Ribonuclease P |
|  | DGo_CA0433 | Ribonuclease PH |
|  | DGo_CA0440 | Ribonuclease R |
|  | DGo_CA0276 | Ribonuclease Z |
|  | DGo_CA2271 | Ribonuclease |
|  | DGo_CA0979 | Guanyl-specific ribonuclease |
| polymerdegrading/modifying enzymes | DGo_CA1058 | putative pullulanases |
|  | DGo_CA1633 | alpha-glucosidases |
|  | DGo_PA0281 | beta-glucosidase |
|  | DGo_CA1180 | maltodextrin glucosidase |
|  | DGo_CA1339 | alpha-galactosidase |
|  | DGo_CA1498 | amylomaltase |
|  | DGo_CA1773 | alpha-glucan phosphorylase |
|  | DGo_CA1724, DGo_CA2610, DGo_CA2433, DGo_CA1210 | glycogen biosynthesis |
|  | DGo_CA0878, DGo_CA2665, DGo_CA2647 | esterases (lipases) |
|  | DGo_CA2548 | polyphosphate kinase |
| Press resistance | DGo_CA2805 | irrE |
|  | DGo_CA1136, DGo_PA0041 | Cold shock proteins |
| antibiotics  resistance | DGo_CA0099, DGo_CA0213, DGo_CA0317, DGo_CA0631, DGo_CA0789, DGo_CA1056, DGo_CA1075, DGo_CA1264, DGo_CA1274, DGo_CA1549, DGo_CA1821, DGo_CA2266, DGo_CA2383, DGo_CA2648, DGo_CA2821, DGo_PB0301, DGo_PB0390, DGo_PC0037 | putative β-lactamases |
|  | DGo_CA0368, DGo_CA0452, DGo_CA0676, DGo_CA0786, DGo_CA0941, DGo_CA1145, DGo_CA1323, DGo_CA1521, DGo_CA2296, DGo_CA2465, DGo_CA2697, DGo_CA2698, DGo_CA2707, DGo_PA0223, DGo_PA0350, DGo_PB0098, DGo_PD0060 | drug efflux proteins |
|  | DGo_CA2225 | putative resistance to florfenicol |
|  | DGo_CA2181 | putative resistance to fosmidomycin |
|  | DGo_CA0978, DGo_PC0199 | putative resistance to bacitracin |
|  | DGo_CA0830 | putative resistance to vancomycin |
| vitamin/cofactor synthesis | DGo_CA2761 | folylpolyglutamate synthase |
|  | DGo_CA2804 | dihydropteroate synthase |
|  | DGo_CA0403, DGo_CA0548 | molybdopterin biosynthesis |
|  | DGo_CA0131, DGo_CA0195, DGo_CA0561, DGo_CA0562, DGo_CA0563, DGo_CA1832, DGo_CA2498, DGo_CA2825, DGo_CA2891, DGo_CA2892, DGo_PA0212 | thiamine biosynthessis |
|  | DGo_CA0162, DGo_CA0164 | riboflavin synthase |
|  | DGo_CA0586, DGo_PA0002, DGo_PA0380, DGo_PA0381, DGo_CA0804, DGo_CA0806, DGo_CA0808, DGo_CA0803, DGo_CA2389, DGo_CA0839, DGo_PA0001, DGo_PA0383 | B12 biosynthesis |
|  | DGo_CA0451, DGo_CA0881, DGo_CA0882, DGo_CA0913, DGo_CA2358, DGo_CA2369, DGo_CA2871, DGo_CA2876, DGo_CA2878 | Carotenoid biosynthesis |
| other | DGo_CA0607, DGo_PA0134, DGo_PB0391 | photolyase |
|  | DGo_CA0998, DGo_CA1464, DGo_CA1830, DGo_CA2870, DGo_PA0179, DGo_PB0312 | Cytochrome P450 |
|  | DGo_CA0240 | Mercuric reductase |
|  | DGo_CA1190 | 2-nitropropane dioxygenase |
|  | DGo_PB0053,DGo_PB0304,DGo_PC0224,DGo_PF0032 | Arsenate reductase |
